# Thermal–Environmental Coupling Wear Mechanism of H13 Steel Disk Cutters in Tunnel Boring Machine Excavation of Composite Strata

**DOI:** 10.3390/ma19173735

**Published:** 2026-09-02

**Authors:** Yungui Pan, Youliang Chen, Jinbo Xie, Xi Du, Tomás Manuel Fernandez-Steeger

**Affiliations:** 1 Department of Civil Engineering, University of Shanghai for Science and Technology, 516 Jungong Road, Shanghai 200093, China; panyungui1997@163.com (Y.P.);; 2 Department of Engineering Geology, Institute of Applied Geosciences, Technical University of Berlin, Ernst-Reuter-Platz 1, 10587 Berlin, Germany

**Keywords:** H13 steel disc cutter, thermal–environmental coupling wear, microstructural damage, composite strata, wear coefficient quantification, multi-factor influence ranking

## Abstract

This study addresses abnormal cutter wear in H13 steel disc cutters during tunnel boring machine excavation of composite strata, where transient frictional shearing temperatures interact with environmental degradation. Taking the Xiangshan Tunnel at 160 m overburden depth as the engineering background, a coupled FLAC3D-PFC3D model was developed to resolve the coupled thermo-mechanical process, and a relative wear model based on the Holm method was formulated to incorporate sub-microscopic microstructural damage and quantify wear evolution. Numerical simulations elucidated the coupled thermal evolution mechanism at the cutter–rock interface. Systematic analyses were performed on single-cutter wear under coupled cutting temperature–chemical corrosion and cutting temperature–freeze–thaw cycling, together with the total wear for single-, double-, and triple-cutter configurations. Under temperature–chemical corrosion coupling, the peak wear coefficient of H13 steel reached 1.45 at 600 °C and pH 6. Under temperature–freeze–thaw cycling coupling, H13 steel peaked at 1.75 at 600 °C with 14 freeze–thaw cycles. Under triple-factor coupling, single-cutter wear was primarily influenced by freeze–thaw damage, double-cutter wear by penetration depth and chemical corrosion, and triple-cutter wear by cutter spacing and chemical corrosion. The chemical corrosion proportion in the double-cutter interface reached 89.4%. At 600 °C, thermal softening drastically amplified environmental degradation abrasion in the primary rock material. Chemical–thermal coupling exhibited greater influence than freeze–thaw cycling, and preferential chemical dissolution in the interface zone compressed the cutter parameter regulation space to its limit. The wear coefficient quantification and factor influence ranking reflect the multi-factor-coupled wear evolution in transportation tunnel composite strata. The parameter sensitivity data provides quantitative references for cutter replacement cycles, advanced parameter selection, and per-kilometer cost variations in tunnel construction management.

## 1. Introduction

TBM cutter wear critically constrains tunneling efficiency and cost control in composite strata. It directly determines cutter replacement cycles, downtime frequency, and per-kilometer advanced cost. This problem intensifies in deep and long tunnels. Disc cutters serve as the core rock-breaking element of the TBM cutterhead. Their wear rate and failure mode govern advanced speed and rock-breaking energy consumption. They also directly influence excavation face stability, support timing, and safety risk management. Relevant wear evolution mechanisms, accurate prediction models, and effective life-extension strategies thus hold significant engineering and theoretical value. They improve TBM construction economy and ensure safe, efficient tunnel advancement. Existing research has systematically explored cutter wear mechanisms and prediction methods from three dimensions: numerical simulation, experimental validation, and field monitoring.

In rock-breaking mechanisms and numerical simulation, Cho et al. [1] validated the effectiveness of explicit dynamic numerical methods in specific energy and fragment mass prediction through linear cutting machine tests. Zhang et al. [2] revealed the eccentric wear mechanism caused by contact area differences between the inner and outer layers of disc cutters and proposed a transitional edge angle design theory. Kun et al. [3] and Zhao et al. [4] revealed rock mass damage evolution laws under complex geological conditions from microscopic numerical simulation and three-dimensional finite element perspectives, respectively. Yang et al. [5] systematically analyzed the effects of penetration depth, rotational speed, and stratum interface inclination on the rock-breaking process. Jiang et al. [6] revealed the dominant mode differences between shear and tensile failure based on the cohesive element method. Wang et al. [7] found, through PFC simulation, that eccentric wear causes sudden cutter force changes, and adjacent cutter height differences reduce rock-breaking efficiency. Yu et al. [8] established a multivariate regression prediction equation for rock-breaking energy with respect to the rock layer’s dip angle and time, indicating minimum energy consumption at a 75° dip angle.

In wear prediction, data-driven methods have become a research hotspot. Mo et al. [9] constructed an IPSO-LSTM hybrid model, achieving an R^2^ = 0.70 prediction accuracy in the Guangzhou Metro Line 18 project. Kwon et al. [10] introduced an equivalent elastic modulus to characterize composite strata properties, employed a SMOTE-enhanced XGB model to achieve 0.94 accuracy, and revealed that plenum pressure exceeding 2 bar is a critical threshold for abnormal wear. Ren et al. [11] established a friction energy-wear prediction model, validated by the Yintao Project and Guangzhou Metro Line 7, and Yang et al. [12] proposed an STFO-KNN dual-modal fusion framework, with an RF fusion model achieving 0.97 binary classification accuracy. Ghorbani and Yagiz [13] found that density and Brazilian tensile strength were optimal input parameters, with their GB-SA model’s R^2^ reaching 0.8274. Wang et al. [14] proposed an MMACNN real-time prediction method, with cumulative wear predictions reaching an average accuracy of 95.75%, and Akhlaghi et al. [15] constructed an XGBoost model through acoustic–vibration signal fusion, with an R^2^ reaching 0.9424.

A cutter’s cutting thermal effects are an important physical mechanism that induces abnormal cutter wear. Wang et al. [16] found that the Cerchar abrasiveness index significantly affects wear, and cutter thrust and stable temperature exhibit an arctangent relationship, such that, under high thrust, a temperature rise causes a hardness decrease, leading to plowing plastic deformation wear. Zhang et al. [17] proposed a distributed laser theoretical model, which suppressed glass glaze formation through an unirradiated zone that buffered heat accumulation, increasing the optimal cutter-groove spacing by 2 mm, while Kang et al. [18] found that, through large-scale physical model tests, increasing the cutter spacing significantly aggravates cutterhead vibration and deformation, and they recommended prioritizing the use of smaller cutter spacing. Deng et al. [19] found that average normal force and rolling force exhibited Boltzmann growth with the cutting depth, and proposed design optimization recommendations for center zone lateral force monitoring and peripheral zone hardness enhancement. Karimi et al. [20] confirmed that UCS and CAI correlate with a cutter’s lifespan better than rock mass parameters, with 19-inch cutters enduring approximately 20–50% longer than 17-inch cutters.

Composite strata heterogeneity significantly affects wear behavior. Zhang and Shen [21] found that under high plenum pressure and low penetration, soft rock section cutters undergo sliding rather than rolling, proposing a semi-empirical wear prediction model considering normal force variation. Liu et al. [22] revealed the coupled influence of joint shear strength on crack patterns and rock-breaking efficiency through DIC systems and PFC2D simulation. Zou et al. [23] found that the wear volume increases approximately linearly with the installation radius and exhibits a first-increase-then-decrease trend with dip angle. They verified the equivalence between particle velocity and transient impact rock-breaking behavior through thermal–hydraulic–mechanical triaxial tests.

To improve wear resistance, Lin et al. [24] employed plasma transferred arc welding to prepare Fe-based amorphous coatings, reducing the wear rate to 20.3% of that of conventional cutters, with the wear mechanism transforming from ploughing–micro-cutting to micro-cutting–microcracking. Existing research has systematically explored cutter wear mechanisms and prediction methods from numerical simulations, experimental validation, and field monitoring dimensions, achieving significant progress in rock-breaking mechanisms, wear prediction, cutting thermal effects, and the influence of composite strata heterogeneity. Most existing studies isolate individual damage factors, addressing either thermal effects, chemical corrosion, or freeze–thaw cycling independently, while the synergistic wear evolution under coupled thermal–environmental loading remains inadequately characterized. Prevailing wear prediction models rely predominantly on macroscopic mechanical responses or data-driven correlations, lacking explicit physical links to mesoscopic mineral deterioration, such as lattice thermal expansion, phase transitions, and chemical dissolution of quartz and feldspar. Consequently, the mesoscopic deterioration mechanisms of mineral components under thermal–chemical or thermal–freeze–thaw coupling, and their quantitative upscaling to macroscopic cutter wear, remain to be elucidated.

To this end, this study addresses abnormal wear of H13 steel disc cutters under coupled thermal–environmental damage in TBM excavation of composite strata. The central objective is to bridge mesoscopic mineral deterioration with macroscopic cutter wear, thereby furnishing a physically grounded wear coefficient framework for tool-material life prediction and replacement cycle optimization. Departing from previous studies that treated thermal effects, chemical corrosion, and freeze–thaw cycling as isolated factors, this work establishes a coupled thermal–chemical–freeze–thaw wear evolution framework. Unlike prevailing macroscopic mechanical or data-driven models, a hardness weakening model was formulated based on Holm wear theory by introducing mineral sub-microscopic damage factors, including lattice thermal expansion, quartz-phase transitions, and feldspar chemical dissolution. This approach quantitatively correlates mesoscopic mineral deterioration with macroscopic wear through the hardness weakening coefficient. Furthermore, differential wear mechanisms across sandstone–granite interfaces were resolved through segmented volumetric wear models. A FLAC3D-PFC3D coupled model was established through the Wall-Zone Structure component, achieving bidirectional force–velocity coupling. Systematic analyses were conducted at 200 °C, 400 °C, and 600 °C on single-cutter wear under temperature–chemical corrosion and temperature–freeze–thaw cycling, and on total wear for single-, double-, and triple-cutter configurations. The temperature range spans from normal cutting equilibrium to extreme malfunction conditions, while pH 4–7 covers groundwater chemistry from acidic to neutral, and freeze–thaw cycles of 5–14 correspond to seasonal damage accumulation over one to three years in subtropical mountainous tunnel projects.

## 2. Establishment of TBM Composite Strata Excavation Model Considering Cutting Thermal Effects

### 2.1. Construction of Sandstone–Granite Composite Strata FLAC3D-PFC3D Coupled Model

The Xiangshan Tunnel in Longyan, Fujian traverses sandstone–granite composite strata at a burial depth of approximately 160 m [25]. TBM cutters face significant wear challenges in this soft–hard composite stratum. The strength difference and mineral heterogeneity between the upper sandstone and lower granite produce complex cutter stress states. Groundwater chemistry changes upon exposure to fault fracture zones. Consequently, cutter wear prediction and advance parameter optimization become the core technical challenges for tunnel construction in this stratum.

**Figure 1:**
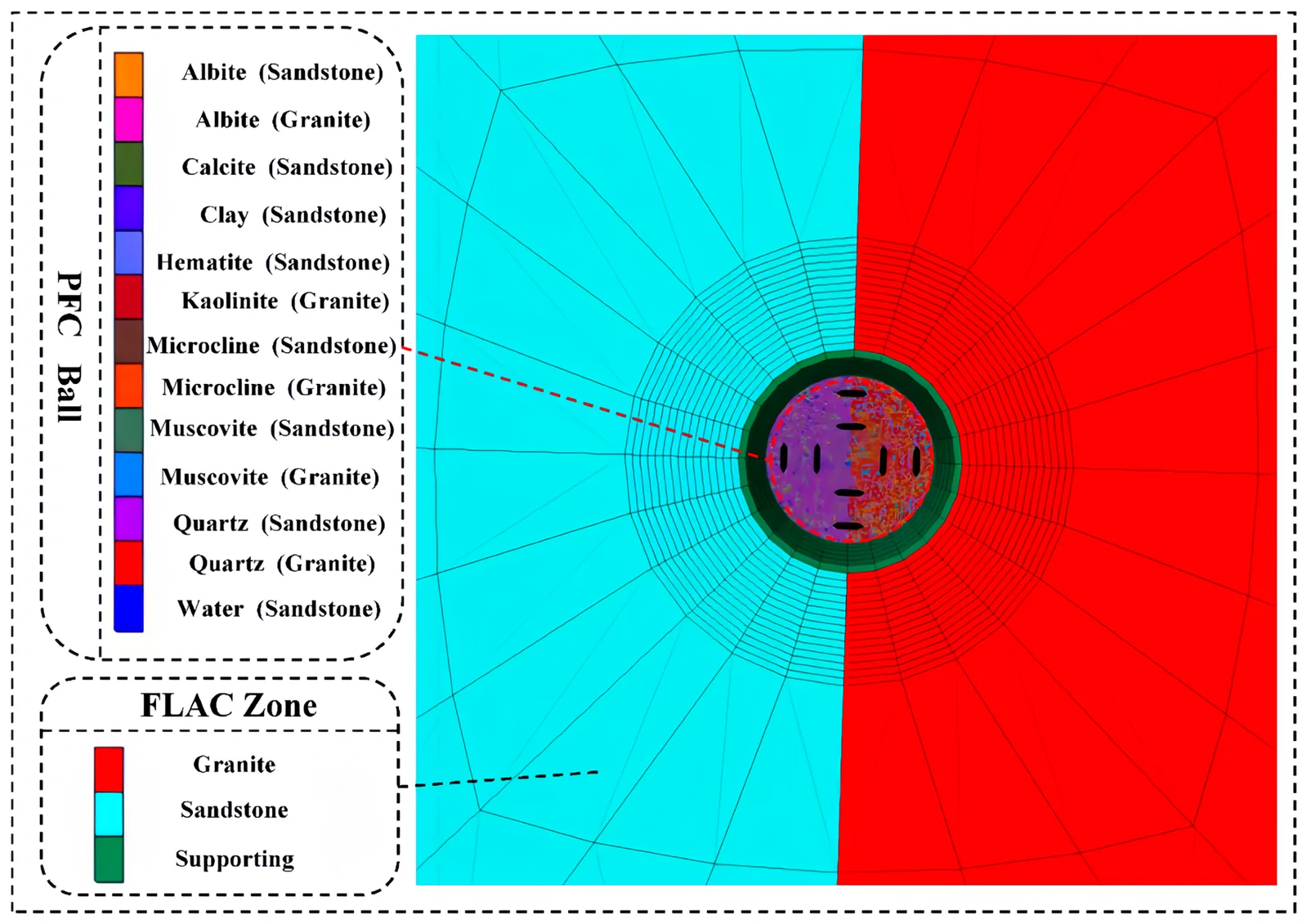
Mineral grouping and geometric configuration of FLAC3D-PFC3D coupled model.

As shown in Figure 1, this study establishes a FLAC3D-PFC3D coupled model with upper sandstone and lower granite. Both PFC3D and FLAC3D software used version 6.0. Mineral components and particle grouping follow the quantitative analysis of Chen et al. [26]. The FLAC3D domain characterizes the macroscopic stress–strain response of surrounding rock, and the central PFC3D domain simulates the dynamic rock-breaking effect of disc cutters through annular cutting. The peripheral coupling boundary of PFC3D transfers the tectonic stress and deformation calculated by FLAC3D to the particle assembly, forming a flexible boundary that accommodates both cutter dynamic characteristics and far-field structural responses. This scheme achieves a balance between computational efficiency and physical fidelity, providing a numerical framework close to engineering practice for TBM excavation in composite strata.

**Figure 2:**
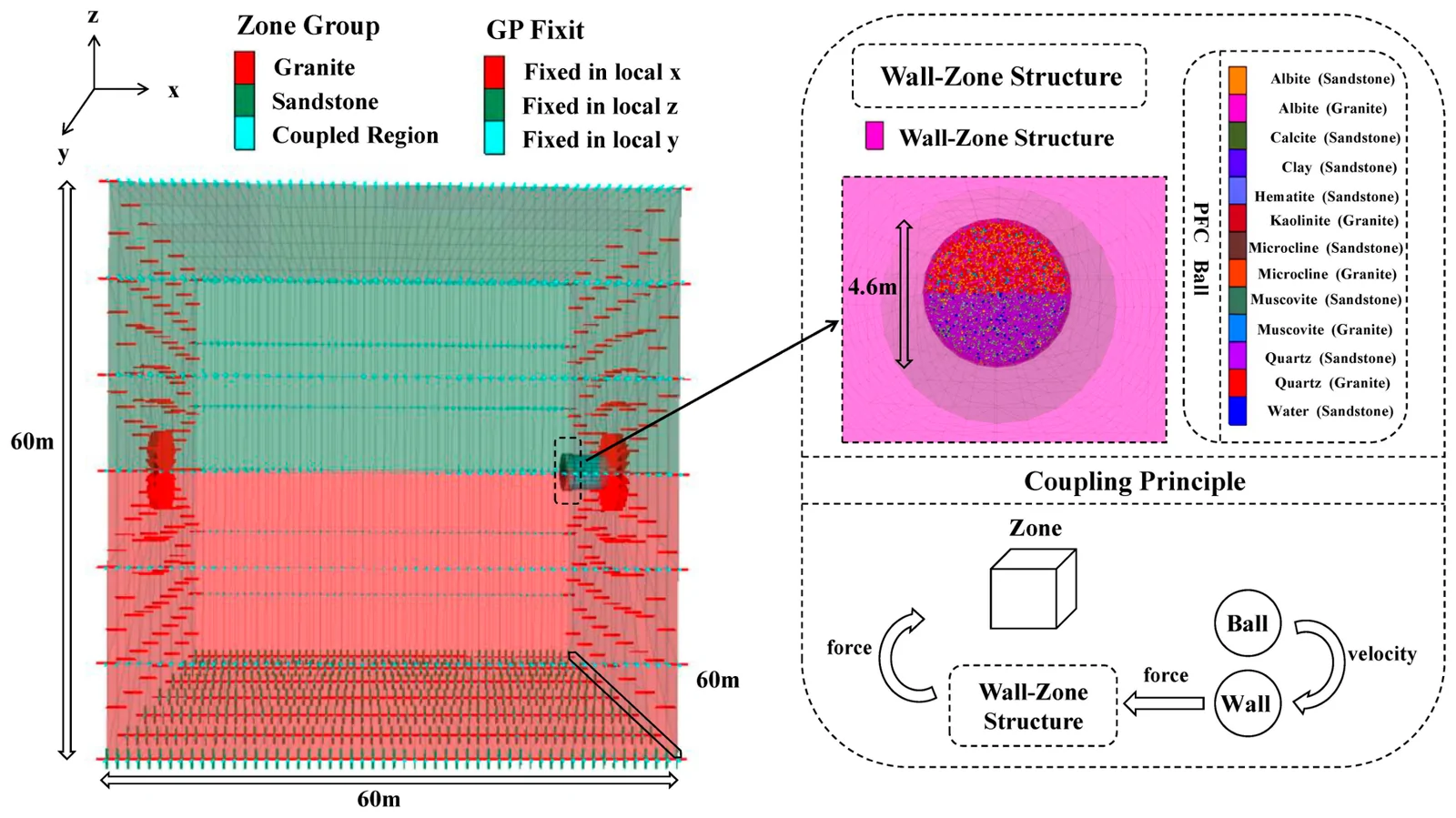
Boundary conditions and continuous-discontinuous coupling mechanism of composite strata TBM excavation model.

As shown in Figure 2, the overall model dimensions are 60 m × 60 m × 60 m, with boundary conditions set as displacement constraints applied to the left, right, front, and rear sides, fixed bottom, and free top to simulate unconstrained surface deformation. The tunnel diameter is 4.6 m, with the horizontal centerline as the boundary, sandstone occurring in the upper section, and granite in the lower section. The PFC3D rock-breaking core zone comprises spherical particles with radii ranging from 28 mm to 46.48 mm, an initial generation porosity of 0.08, and an initial generation density of 2700 kg/m^3^. The wall stiffness in the initial generation is 15 GPa, and the wall-to-ball stiffness ratio is 1.5. A coupling transition zone is arranged around the periphery of the PFC3D rock-breaking core zone, achieving information exchange between continuous and discontinuous media through the Wall-Zone Structure component. Contact forces generated at PFC particle–boundary wall contacts are transferred to FLAC3D zone boundary elements via the coupling component, and the element stress and deformation calculated by FLAC3D react back on the PFC boundary walls, forming a bidirectional force–velocity coupling cycle. This flexible boundary mechanism overcomes the limitation of traditional PFC rigid wall boundaries that cannot deform, enabling PFC3D to receive far-field tectonic stress and displacement boundary effects transmitted from FLAC3D while feeding back dynamic disturbances generated by cutter rock-breaking to the macroscopic continuous domain, achieving coordinated simulation of mesoscopic rock-breaking mechanisms and macroscopic surrounding rock responses in TBM excavation of composite strata.

The disc cutter employs a rolling resistance linear contact model. The contact stiffness is characterized by an effective modulus of 8 × 10^6^ Pa with a normal-to-shear stiffness ratio of 1. A damping ratio of 1 is assigned to suppress non-physical contact oscillations. The interface friction coefficient is set to 0.05, and the rolling resistance friction coefficient is set to 0.05, which together govern the tangential resistance and rotational restraint at the cutter tip. These parameters enable quantitative characterization of cutter tip forces and particle fracture morphology during the cutting process. The rock-cutting domain adopts the Parallel Bond damage contact model with micro-mechanical parameters quantified through the damage factor, as detailed in Table 1. Thermal interaction at the cutter–rock interface is governed by a heat-pipe contact model, where heat conduction through the cutter tip drives the interfacial temperature field and feeds back into the contact force evolution. The mechanical calculation employs a sub-step setting of 5 within each solution cycle. The equilibrium convergence criterion adopts the system default of 1 × 10^−5^.

### 2.2. Inversion of In-Situ Stress Field Based on Engineering Analogy and Determination of Boundary Conditions

As shown in Table 2, the measured in situ stress data in the engineering geological documentation of the Xiangshan Tunnel project are limited, making it difficult to directly determine the initial stress field distribution at the 160 m burial depth. To address this, the ZK11B-6 Haojiagou engineering case [27] was introduced as an analogy basis: this tunnel also traverses sandstone–granite composite strata, with rock mechanical properties highly consistent with those of the Xiangshan Tunnel, a uniaxial compressive strength range of 40–120 MPa covering the 58.7–99.7 MPa of the Xiangshan Tunnel, and a burial depth range of 133.98–212.68 m that completely encompasses the 160 m excavation depth of the Xiangshan Tunnel.

**Figure 3:**
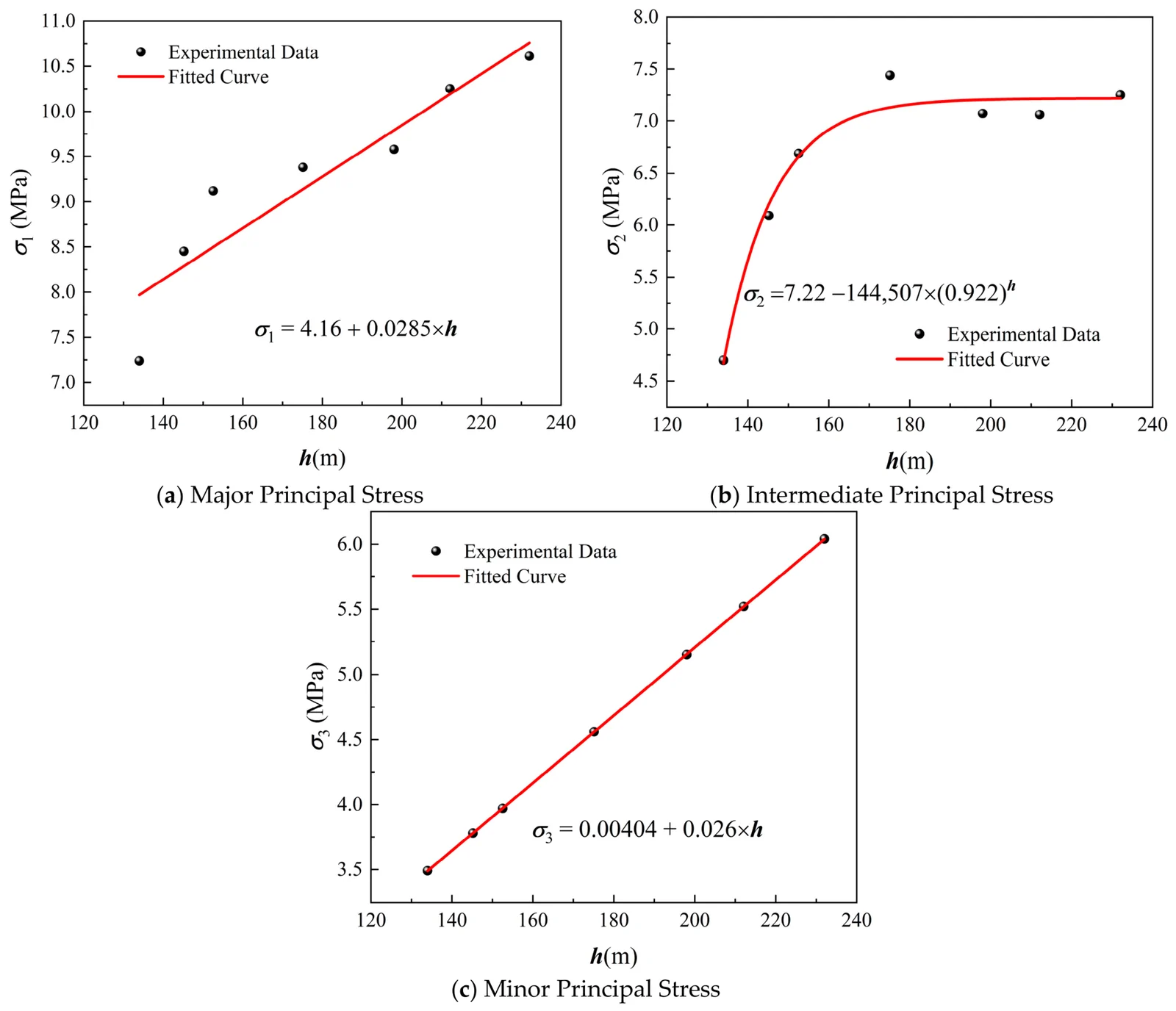
Regression fitting curves of measured in situ stress with burial depth for Haojiagou [27].

As shown in Figure 3, based on geological condition similarities and burial depth coverage, measured in situ stress data from Haojiagou were employed to establish a regression model, fitting the functional distributions of major, intermediate, and minor principal stresses with burial depth, and subsequently extrapolating to calculate the initial stress field at a depth of 160 m in the Xiangshan Tunnel. The results indicate a maximum principal stress of 8.71 MPa, intermediate principal stress of 6.91 MPa, and minimum principal stress of 4.16 MPa, which serve as the far-field stress boundary input for the FLAC3D-PFC3D coupled model, ensuring that the mechanical boundary conditions of the numerical simulation possess geological representativeness analogous to engineering practice.

## 3. Realization of Cutter Cutting Thermal Effect and Multi-Physics Coupling Verification

### 3.1. Calibration and Experimental Verification of Cutter Rock-Breaking Model Without Thermal Effect

**Figure 4:**
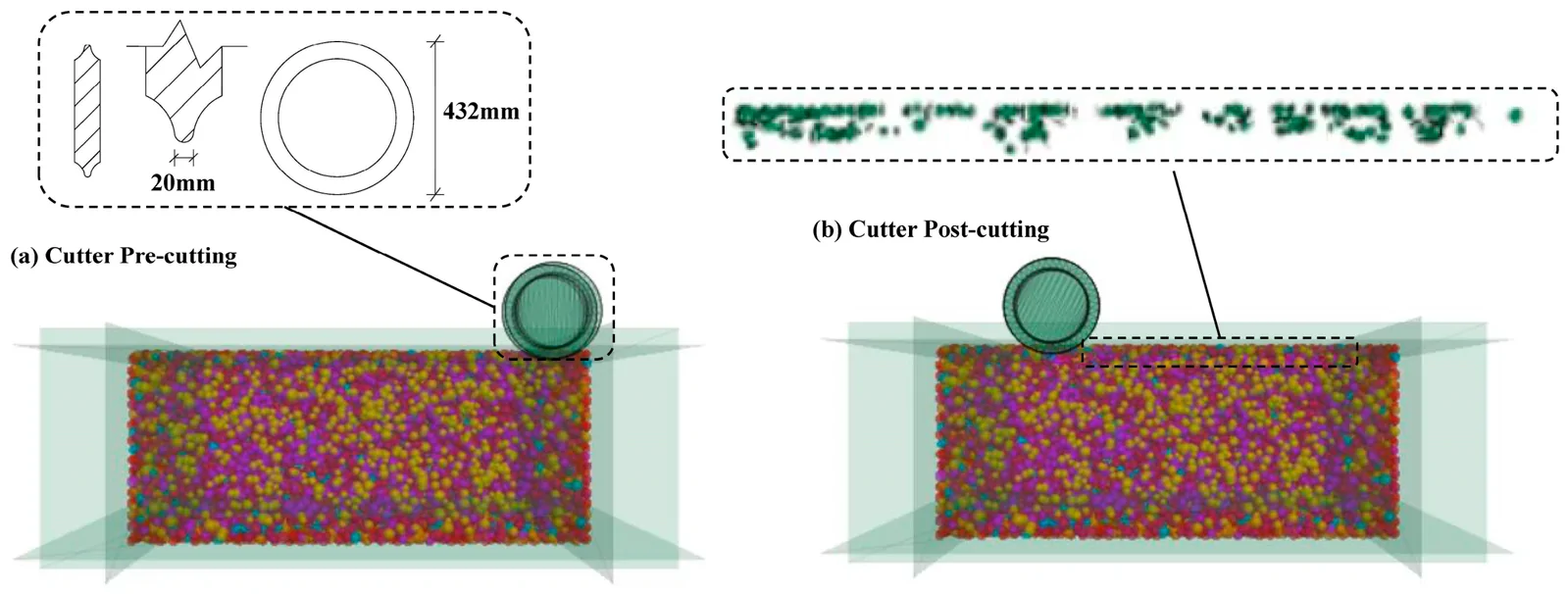
Geometric parameters and pre- and post-cutting configurations of PFC3D cutter linear cutting test.

As shown in Figure 4, to verify the reliability of the PFC3D cutter rock-breaking model, linear cutting numerical simulation calibration tests were conducted, benchmarking against the indoor linear cutting test results of Cho et al. [1]. The grain classification in this figure aligns with the grouping categories shown in Figure 2. Cutter geometric parameters refer to actual TBM cutter specifications: a cutter ring diameter of 432 mm and a cutter tip width of 20 mm. Three cutter spacing conditions of 28 mm, 40 mm, and 48 mm were set in the numerical simulation, with penetration fixed at 4.0 mm/rev, consistent with the test conditions of Cho et al. [1]. Average rolling force and rock-breaking specific energy were selected as dual verification indicators: the former reflects the mechanical intensity of cutter–rock interaction, while the latter characterizes rock-breaking energy efficiency; together they constrain the physical rationality of the model in both force and energy dimensions.

As shown in Table 3, except for the average rolling force error of 9.4% under the 48 mm cutter spacing condition, the errors of average rolling force and rock-breaking specific energy under all other conditions are controlled within 5%, with the minimum specific energy error reaching 0.9%. The relatively large error under the 48 mm condition originates from the complication of crack coalescence paths at large spacing, where the non-local effect of inter-particle contact force chains intensifies, leading to inherent discreteness in the numerical model’s capture of far-field crack propagation; this phenomenon constitutes an acceptable systematic deviation in PFC discrete element simulation. Overall, the multi-condition cross-validation of dual indicators demonstrates that the cutter geometric modeling, contact parameter calibration, and cutting condition settings in this study can reasonably reproduce the mechanical responses and energy dissipation characteristics of indoor tests, providing an experimentally validated modeling benchmark for subsequent TBM excavation simulation in composite strata.

### 3.2. Multi-Physics Coupled Evolution Mechanism of Cutter Cutting Thermal Effect in Composite Strata

**Figure 5:**
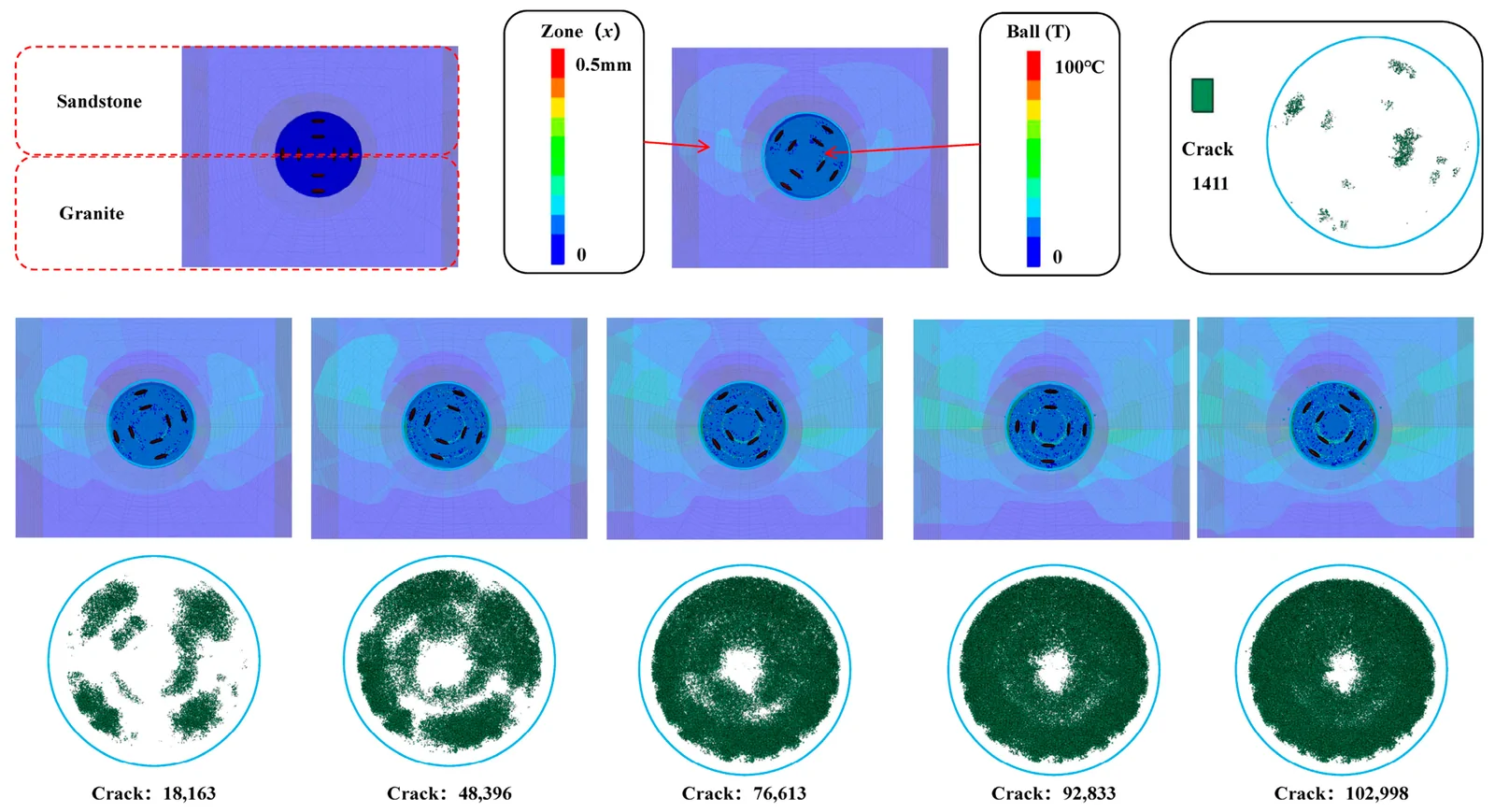
Temperature–displacement–crack field evolution in sandstone–granite composite strata under cutting thermal effect.

Figure 5 illustrates the multi-physics coupled evolution mechanism of cutter cutting thermal effects in sandstone–granite composite strata. Cutting frictional heat is generated at the cutter–rock contact interface and diffuses laterally across the tunnel face from the cutting ring center through inter-particle thermal conduction; the displacement field exhibits inter-layer asymmetric distribution due to the hardness difference between the upper sandstone and lower granite. Crack development lags significantly behind the establishment of the temperature field, initially appearing as discrete clusters distributed on the sandstone side, and gradually connecting circumferentially as the heat transfer range expands, ultimately forming a throughgoing annular damage zone. The crack density in the sandstone region is systematically higher than that in the granite, indicating that the inter-layer heterogeneity of composite strata exerts significant modulation on the thermal–mechanical coupled damage pattern.

**Figure 6:**
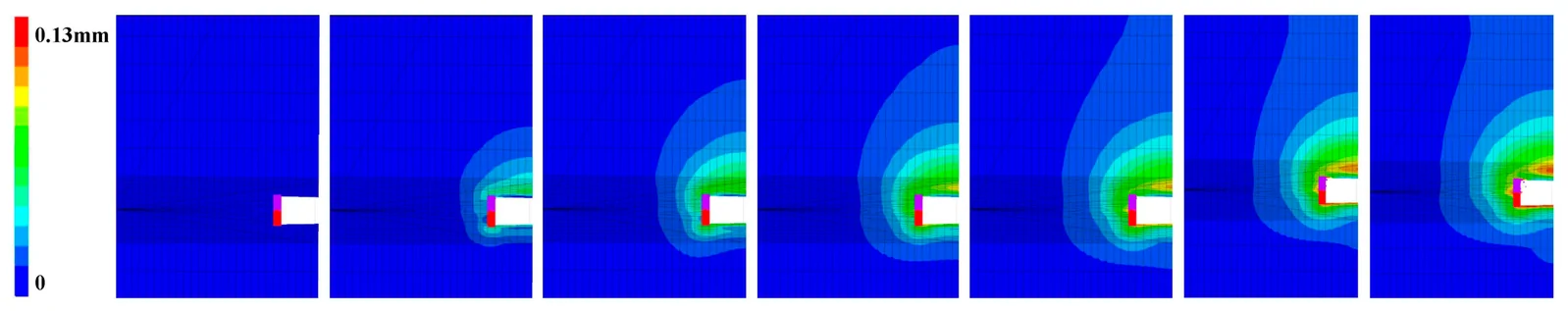
Spatiotemporal extension of surrounding rock displacement field in composite strata driven by cutter cutting thermal effect.

Figure 6 reveals the spatiotemporal evolution law of surrounding rock displacement field under the cutter cutting heat release effect: as the cutting process advances, the temperature thermal effect not only induces local deformation of the tunnel face rock mass, but also drives the lateral surrounding rock displacement field to expand continuously from the cutting front toward deeper regions, with both the influence range and amplitude of displacement increasing significantly with heat accumulation.

## 4. Wear Evolution Analysis of Composite Strata Under Cutter Cutting Thermal–Environmental Damage Coupling

### 4.1. Establishment of Relative Wear Model for Disc Cutters Based on Sub-Microscopic Mineral Damage

Existing cutter wear research predominantly focuses on macroscopic mechanical responses, while the quantitative mapping of mineral component differential evolution to hardness degradation remains insufficient. However, rock is essentially a multiphase aggregate cemented by mineral grains such as quartz, feldspar, and mica, with significant differences in hardness, thermal expansion coefficient, and chemical stability among these minerals. Transient high temperatures from TBM cutter cutting and acidic groundwater chemical erosion act synergistically on mineral lattices, causing thermal expansion distortion and chemical dissolution. This mineral-scale damage accumulation transfers to macroscopic rock mass mechanical behavior through size effects, manifested as reduced abrasiveness and altered fragmentation modes, ultimately modifying the wear dynamics at the cutter–rock interface.

As shown in Equation (1), Holm theory frames volumetric wear as a function of effective friction work and surface hardness, offering distinct advantages for this study. The friction work formulation directly couples with the mechanical energy dissipation calculated from the PFC3D cutter–rock interaction model, enabling seamless integration between numerical simulation and wear quantification. The hardness parameter *H* provides a natural entry point for introducing mineral-specific sub-microscopic damage factors, allowing the model to translate quartz-phase transitions, feldspar chemical dissolution, and cementitious thermal softening into quantitative hardness degradation. This meso-to-macro bridging capability is essential for capturing the thermal–environmental coupling effects central to this investigation. The effective friction work is determined by the rolling friction work done by the vertical contact force along the cutting path, and the rolling friction work is further expressed as the product of the friction coefficient, normal force, and cutting distance. The specific expression is given in Equation (2).

(1) $$V=\frac{KW}{H}$$

In the formulation, K denotes the proportional constant, *W* is the effective friction work, *H* is the surface hardness, and V is the volume wear.

(2) W=μFL

In the formulation, μ denotes the kinetic friction coefficient of the material, *F* is the normal force, and *L* is the cutting distance.

Substituting Equation (2) into Equation (1) yields the volumetric wear expression under varying normal forces, as shown in Equation (3).

(3) $$V=\frac{K\mu FL}{H}$$

As shown in Equation (4), the cutting distance is calculated based on the actual motion trajectory of the cutter; for a circular cutting path, its path length is determined by the cutting radius of the cutter and the rotation angle of the cutting motion.

(4) $$L=\frac{\pi Rn}{180}$$

In the formulation, *R* denotes the annular cutting radius of the cutter, and *n* is the rotation angle of the annular cutting motion.

Liu et al. [28] employed grey relational analysis to screen out the five parameters most sensitive to the wear rate of positive cutters, including uniaxial compressive strength, equivalent quartz content, and cutterhead thrust, and established a positive cutter life prediction model through multivariate stepwise regression. This study directly adopts the above field measurement data reported by Liu et al. [28] to calibrate the cutter–rock friction coefficient, as shown in Equation (5).

(5) μ=−0.00121UCS+0.505

In the formulation, UCS denotes the uniaxial compressive strength of rock.

Substituting Equation (5) into Equation (3) yields the volumetric wear calculation expression considering rock mass strength, as shown in Equation (6).

(6) $$V=\frac{KFL\left[-0.00121UCS+0.505\right]}{H}$$

**Figure 7:**
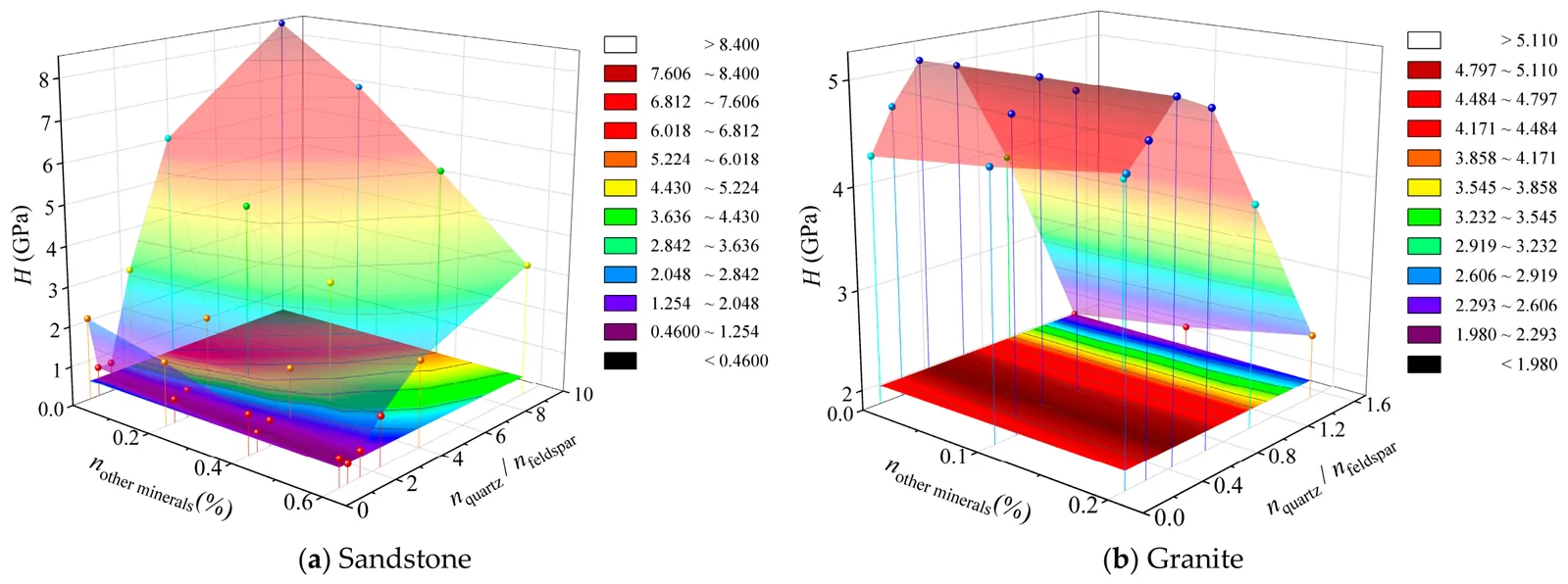
The three-dimensional distribution of cutting hardness for sandstone and granite with respect to the quartz-to-feldspar content ratio and percentages of other minerals [29].

As shown in Figure 7, Li et al. [29] systematically measured the content and grain size of quartz, feldspar, and other minerals based on 96 groups of sandstone and granite samples collected from ten provinces in China and established a prediction model between mineral composition and rock hardness and abrasiveness using BP-ANN. This study directly adopts the above measured data reported by Li et al. [29] to calibrate the cutting hardness of sandstone and granite based on mineral composition dependency.

Based on the controlling effect of the quartz-to-feldspar content ratio on hardness in the model of Li et al. [29], which reflects the evolution of mineral cementation strength and grain contact networks, this ratio, together with the percentages of other minerals, was adopted as independent variables for multivariate linear regression of cutting hardness. The fitted hardness equations for sandstone and granite are established as Equations (7) and (8), respectively.

(7) $$H_{s}=-3.493n_{\mathrm{other}\;\mathrm{minerals}}+0.656\frac{n_{\mathrm{quartz}}}{n_{\mathrm{feldspar}}}+1.473$$

In the formulation, $n_{\mathrm{other}\;\mathrm{minerals}}$ denotes the percentage of minerals other than quartz and feldspar in the rock, $n_{\mathrm{quartz}}$ is the percentage of quartz, $n_{\mathrm{feldspar}}$ is the percentage of feldspar, and $H_{s}$ is the cutting hardness of sandstone.

(8) $$H_{g}=0.821n_{\mathrm{other}\;\mathrm{minerals}}-1.718\frac{n_{\mathrm{quartz}}}{n_{\mathrm{feldspar}}}+5.272$$

In the formulation, $H_{g}$ denotes the cutting hardness of granite.

**Figure 8:**
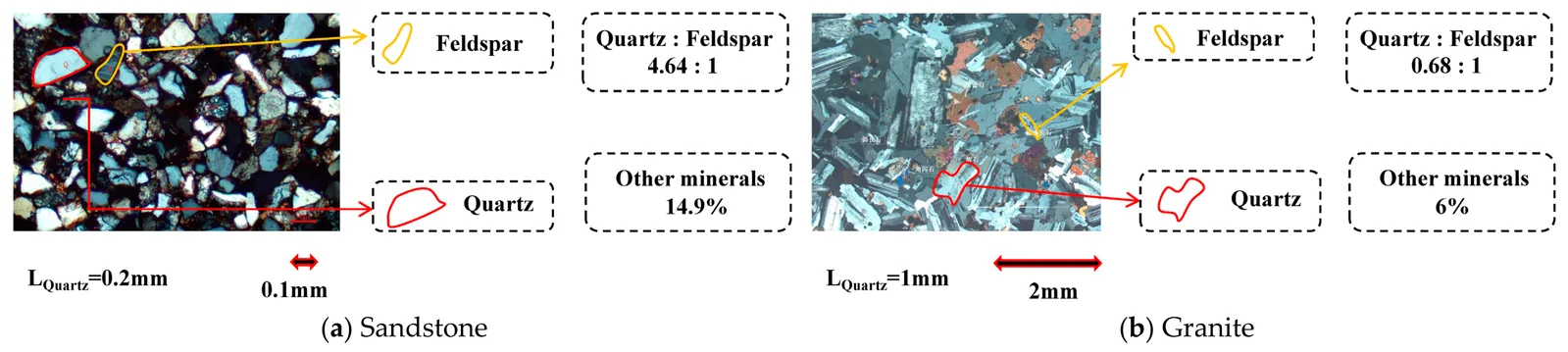
Quantitative characteristics of thin-section microstructure and mineral composition for sandstone and granite [26].

Chen et al. [26] conducted thin-section microscopic analysis on sandstone and granite to quantitatively reveal the mineral compositional differences and spatial distribution characteristics of the two rock types. As shown in Figure 8, the quartz-to-feldspar content ratio in sandstone is 4.64:1, with other minerals accounting for 14.9%; mineral grains exhibit a fine-grained, scattered distribution at a particle size scale of approximately 0.1 mm, and the cementation structure is relatively loose. In granite, the quartz-to-feldspar content ratio is 0.68:1, with other minerals accounting for 6%; mineral grains exhibit a coarse-grained, interlocking distribution at a particle size scale reaching 2 mm, and the crystalline structure is dense and intact.

The mineral percentages of the two rock types govern their distinct meso-contact hardness and macro-mechanical response evolution. Sandstone adopts a loose cementation mode with quartz as the skeleton and feldspar as the filler, making it susceptible to particle debonding and hardness degradation under thermal–chemical coupling. Granite adopts a dense crystalline mode with feldspar as the primary phase and quartz as the auxiliary phase, which endows it with higher initial hardness and damage resistance. The above mineral compositional data provide a direct basis for the parameter values in the hardness equations of this study, enabling the mineral component variables in Equations (7) and (8) to establish quantitative correspondence with the meso-structures of real rock samples.

Fu et al. [30] employed modified H13 steel as the material for the cutter ring, with a surface hardness of 58.7 HRC. This material optimizes the alloy composition and heat treatment process based on conventional H13 steel, significantly enhancing the wear resistance and impact resistance of the cutter ring. Given its extensive application and reliability verification in hard rock TBM engineering, the thermal–physical property parameters of the cutter ring material in this numerical simulation are all referenced from the reported values of H13 steel by Fu et al. [30].

Tian et al. [31] employed a high-temperature indentation and wear test system to systematically investigate the evolution of hardness of H13 steel surfacing layers in the temperature range from room temperature to 400 °C. It was found that the material hardness exhibits a monotonically decreasing trend with increasing temperature, with the hardness at 400 °C dropping by approximately 10 HRC compared to room temperature. Concurrently, the oxidation degree of the worn surface intensifies significantly, with the atomic percentage of oxygen increasing from 7.67% to 50.96%.

This study directly adopts the above high-temperature hardness measurement data of H13 steel reported by Tian et al. [31] and performs linear fitting of the temperature–hardness relationship, as shown in Equation (9). It should be noted that the hardness–temperature relationship of Equation (9) is experimentally calibrated only up to 400 °C [31]. The 600 °C condition therefore represents a linear extrapolation beyond the directly validated range, and the corresponding wear simulations are best interpreted as exploratory extreme-condition scenarios that probe theoretical malfunction boundaries rather than empirically validated operating scenarios. This upper bound is retained to establish the full spectrum of potential thermal softening effects on cutter life prediction, pending future high-temperature experimental verification.

(9) H(T)=9.46517−0.00583474 T

In the formulation, ***T*** denotes the cutter temperature, and H(T) denotes the hardness of H13 steel, considering the cutter temperature effect.

This study extends the temperature–hardness reduction relationship beyond the experimentally validated range based on the above mineral structural evolution law. The underlying mechanism is that increased crystal spacing corresponds to defect proliferation and grain boundary coarsening within minerals, leading to reduced dislocation slip resistance and macroscopic hardness degradation; decreased crystal spacing reflects enhanced crystal preferred orientation and structural densification, which improves the mineral’s resistance to local indentation deformation. By tracking the crystal spacing variation trends of dominant minerals across different temperature intervals, quantitative mapping from microscopic structural deterioration to macroscopic hardness degradation of rock can be realized, providing a mineralogical constitutive basis for wear calculation under the cutter cutting temperature field.

Pawlus and Reizer [32] pointed out that the hardness parameter *H* in the Archard wear equation should take the softer material between the two contacting bodies. Accordingly, this study first calculates the indentation hardness of H13 steel at 600 °C as 5.96 GPa, and the indentation hardness of sandstone and granite at room temperature as 4.0 GPa and 4.15 GPa, respectively. Since the hardness of both rock types is lower than that of the cutter material under high-temperature conditions, the hardness value in the volumetric wear calculation adopts that of the cut rock.

Chen et al. [26] pointed out that temperature elevation induces a differentiated evolution of crystal spacings in quartz, albite, and potassium feldspar: the crystal spacing of quartz decreases overall, whereas albite and potassium feldspar exhibit a staged transition from densification to expansion due to the alternating enhancement and weakening of crystal orientations.

Therefore, the hardness of sandstone and granite, considering sub-microscopic mineral damage, is characterized by Equations (10) and (11), respectively.

(10) $$H_{S}^{D}=\left[-3.493n_{\mathrm{other}\;\mathrm{minerals}}+0.656\frac{n_{\mathrm{quartz}}}{n_{\mathrm{feldspar}}}+1.473\right]\times D_{0}^{S}$$

In the formulation, $H_{S}^{D}$ denotes the hardness considering environmental and cutter cutting thermal damage, and $D_{0}^{S}$ is the sub-microscopic mineral damage factor of sandstone.

(11) $$H_{G}^{D}=\left[0.821n_{\mathrm{other}\;\mathrm{minerals}}-1.718\frac{n_{\mathrm{quartz}}}{n_{\mathrm{feldspar}}}+5.272\right]\times D_{0}^{G}$$

In the formulation, $H_{G}^{D}$ denotes the hardness considering environmental and cutter cutting thermal damage, and $D_{0}^{G}$ is the sub-microscopic mineral damage factor of granite.

Based on the significant differences in mechanical properties and mineral compositions between sandstone and granite, the composite stratum cutting process is divided into sandstone and granite segments for establishing the volumetric wear calculation models. For the sandstone segment, based on the hardness weakening mechanism induced by sub-microscopic mineral damage, its volumetric wear can be expressed as:

(12) $$V_{s}=\frac{\left[-0.00121UCS+0.505\right]\pi KRn_{s}F_{s}}{180\times D_{0}^{S}\left[-3.493n_{\mathrm{other}\;\mathrm{minerals}}+0.656\frac{n_{\mathrm{quartz}}}{n_{\mathrm{feldspar}}}+1.473\right]}$$

In the formulation, $n_{s}$ denotes the cutting angle of the sandstone segment, $F_{s}$ is the cutting normal force of sandstone, and $V_{s}$ is the wear volume of sandstone.

Similarly, for the granite segment, due to its higher quartz content and distinct mineral hardness distribution compared with sandstone, its volumetric wear adopts a differentiated mineral correction coefficient:

(13) $$V_{g}=\frac{\left[-0.00121UCS+0.505\right]\pi KRn_{g}F_{g}}{180D_{0}^{G}\times \left[0.821n_{\mathrm{other}\;\mathrm{minerals}}-1.718\frac{n_{\mathrm{quartz}}}{n_{\mathrm{feldspar}}}+5.272\right]}$$

In the formulation, $n_{g}$ denotes the cutting angle of the granite segment, $F_{g}$ is the cutting normal force of granite, and $V_{g}$ is the wear volume of granite.

Given the gradual transition characteristics of mineral composition at the sandstone–granite composite stratum interface, the mechanical parameters no longer follow the constitutive relationship of a single lithology, necessitating the introduction of an equivalent mean-value treatment strategy. The interface friction coefficient is taken as the arithmetic mean of the friction coefficients of the sandstone and granite segments:

(14) $$u_{\mathrm{sg}}=\frac{u_{s}+u_{g}}{2}$$

Similarly, the equivalent hardness $H_{\mathrm{SG}}^{D}$ at the interface is characterized by the mean of the damage-softened sandstone hardness $H_{S}^{D}$ and granite hardness $H_{G}^{D}$:

(15) $$H_{\mathrm{SG}}^{D}=\frac{H_{G}^{D}+H_{S}^{D}}{2}$$

The total wear volume of the sandstone–granite composite stratum is taken as the sum of the cutting wear volumes of the sandstone segment, granite segment, and interface segment, as shown in Equation (16). This segmented approach reflects the intrinsic lithological heterogeneity of composite strata rather than an arbitrary mathematical partition. The sandstone segment possesses loose quartz-feldspar cementation, the granite segment exhibits dense crystalline interlocking, and the interface segment undergoes stress concentration and preferential chemical dissolution along the lithological mutation zone. The summation therefore preserves the physical fidelity of segment-specific damage mechanisms while furnishing a computationally tractable framework for engineering wear prediction.

(16) $$V=V_{sg}+V_{s}+V_{g}$$

The selection of 600 °C as the extreme temperature boundary and the freeze–thaw cycle settings require explicit engineering justification. Regarding the thermal upper bound, Lyu et al. [33] conducted theoretical and numerical analyses of thermal stresses and temperature variations in disc cutter cutting composite strata under thermal–force multi-fields, revealing that localized frictional heating at the cutter–rock interface can generate extreme temperature anomalies far exceeding the conventional 100–150 °C equilibrium range under abnormal thrust or cooling failure conditions. Tian et al. [31] further confirmed that H13 steel hardness undergoes monotonic degradation up to 400 °C, and the linear temperature–hardness relationship established in this study enables physically grounded extrapolation to 600 °C to capture the full spectrum of thermal softening effects. Regarding freeze–thaw damage, the cycle counts of 5, 8, 11, and 14 correspond to annual damage accumulation over one to three years in seasonally frozen regions under a single-cycle mode from −8 °C to 25 °C, a range representative of the seasonal temperature fluctuations encountered in the Xiangshan Tunnel engineering area and similar transportation tunnel projects in subtropical mountainous zones. The cutter cutting temperature of sandstone and granite under conventional TBM tunneling conditions generally falls within the range of 100–150 °C, reflecting the thermal equilibrium level between the cutter and rock mass under normal construction states. Accordingly, this study selects 100 °C as the baseline temperature for the control group to characterize the standard wear state without abnormal thermal disturbances. On this basis, the wear evolution law at elevated cutter cutting temperatures of 200 °C and 400 °C is systematically investigated, and the aggravating effect of an extreme abnormal high temperature at 600 °C on cutter wear characteristics is further examined [33]. The wear coefficient $k_{v}$ is a dimensionless relative wear index rather than an absolute measure of actual cutter wear volume. It quantifies the proportional amplification or reduction ratio of volumetric wear at different cutting temperatures relative to the 100 °C baseline state. Consequently, statements regarding wear reduction or wear amplification throughout this study refer to relative changes in this normalized index, not to absolute variations in physical wear volume. This dimensionless formulation provides an evaluation index for abnormal cutter temperature early warning and wear control during TBM tunneling.

(17) $$k_{v}=\frac{V}{V_{0}}$$

In the formulation, $V_{0}$ denotes the volumetric wear at 100 °C, V is the volumetric wear at an arbitrary cutting temperature, and $k_{v}$ is the cutter wear coefficient at an arbitrary temperature. A $k_{v}$ greater than 1 indicates wear exceeding that under a normal cutting temperature, while a $k_{v}$ less than 1 indicates wear below that under a normal cutting temperature.

The wear coefficient defined in Equation (17) intrinsically reduces parametric uncertainty by normalizing volumetric wear against the 100 °C baseline state. Because it expresses a ratio rather than an absolute quantity, it functions as a numerical wear index that isolates the relative influence of temperature and environmental damage from field-dependent absolute wear rates. This relative formulation eliminates the absolute dependence on the proportional constant K, the kinetic friction coefficient μ, and the cutting normal force F, all of which exhibit substantial variability across field conditions. The dominant uncertainty source thus reduces to the sub-microscopic mineral damage factor *D*, which is directly constrained by the mineral composition data of Chen et al. [26] and the temperature–hardness fitting error reported by Tian et al. [31]. Sensitivity analysis indicates that the weakening coefficient propagates linearly into the wear coefficient, meaning that a 10% uncertainty in mineral damage factor *D* induces an equivalent 10% uncertainty in the final wear coefficient. This transparent uncertainty propagation path ensures that the model predictions remain traceable and physically bounded.

### 4.2. Wear Characteristics of Composite Strata Under Cutter Cutting Thermal–Chemical Damage Coupling

**Figure 9:**
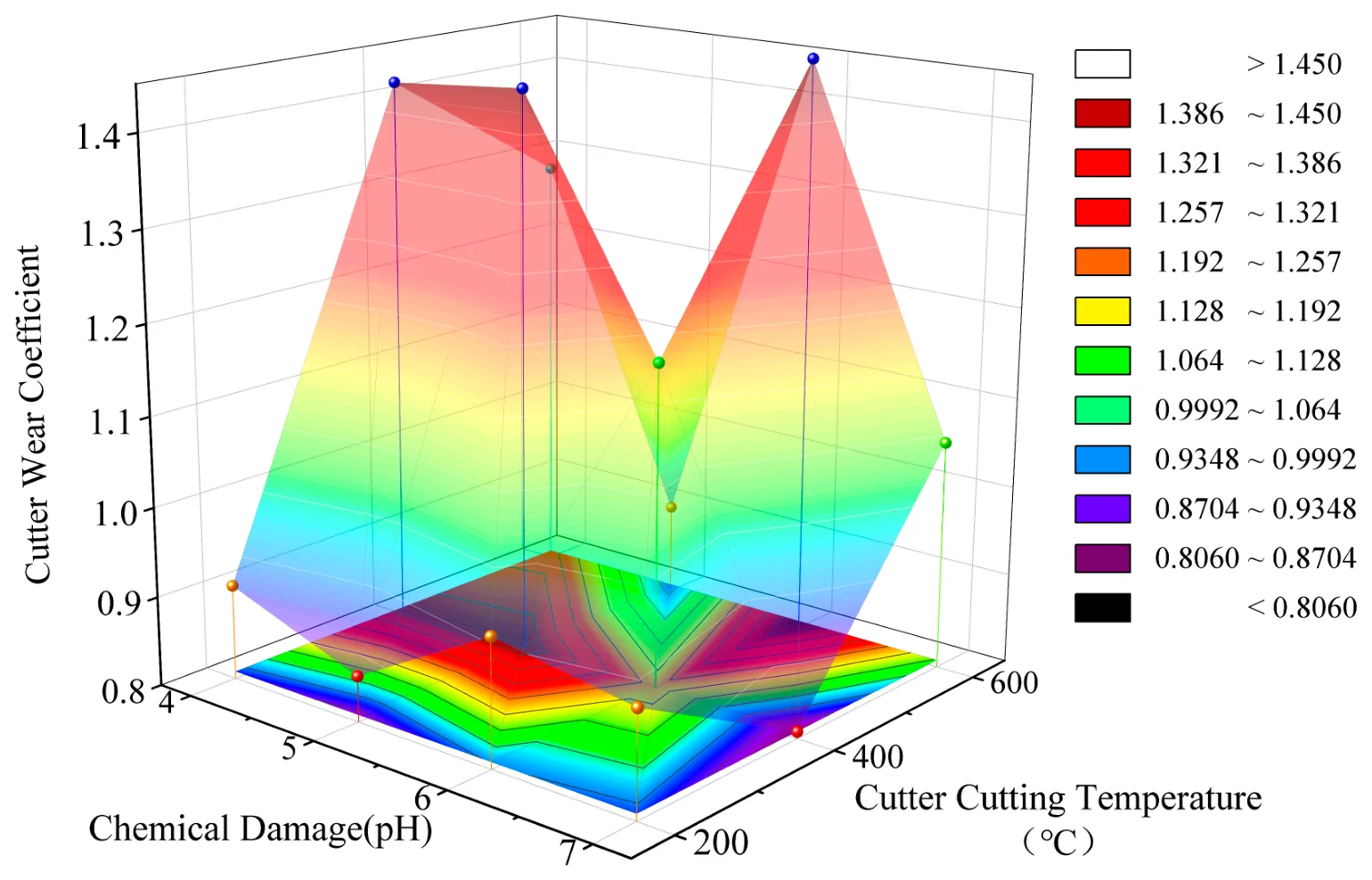
Wear coefficient response surface of single-cutter sandstone section under thermal–chemical coupling with respect to pH value and cutting temperature.

Figure 9 illustrates the wear coefficient response of the single-cutter sandstone section under thermal–chemical coupling. At 200 °C, all wear coefficients range from 0.85 to 0.94, below unity, indicating that mild cementitious softening reduces cutting resistance relative to the baseline state. At 400 °C, coefficients under pH 4 to 6 conditions span 1.17 to 1.43, all exceeding unity, while the pH 7 condition yields 0.81 as the minimum across all test conditions. At 600 °C, the pH 6 condition produces a coefficient of 1.45 as the maximum across all conditions, whereas the pH 5 condition drops abruptly to 0.90.

Under neutral pH 7, chemical attack is effectively suppressed, yielding the minimum wear coefficient at both 400 °C and 600 °C. The temperature–chemical dual-field coupling constitutes the governing mechanism for cutter life control in sandstone sections, with wear amplification peaking at 45% under 600 °C and pH 6.

**Figure 10:**
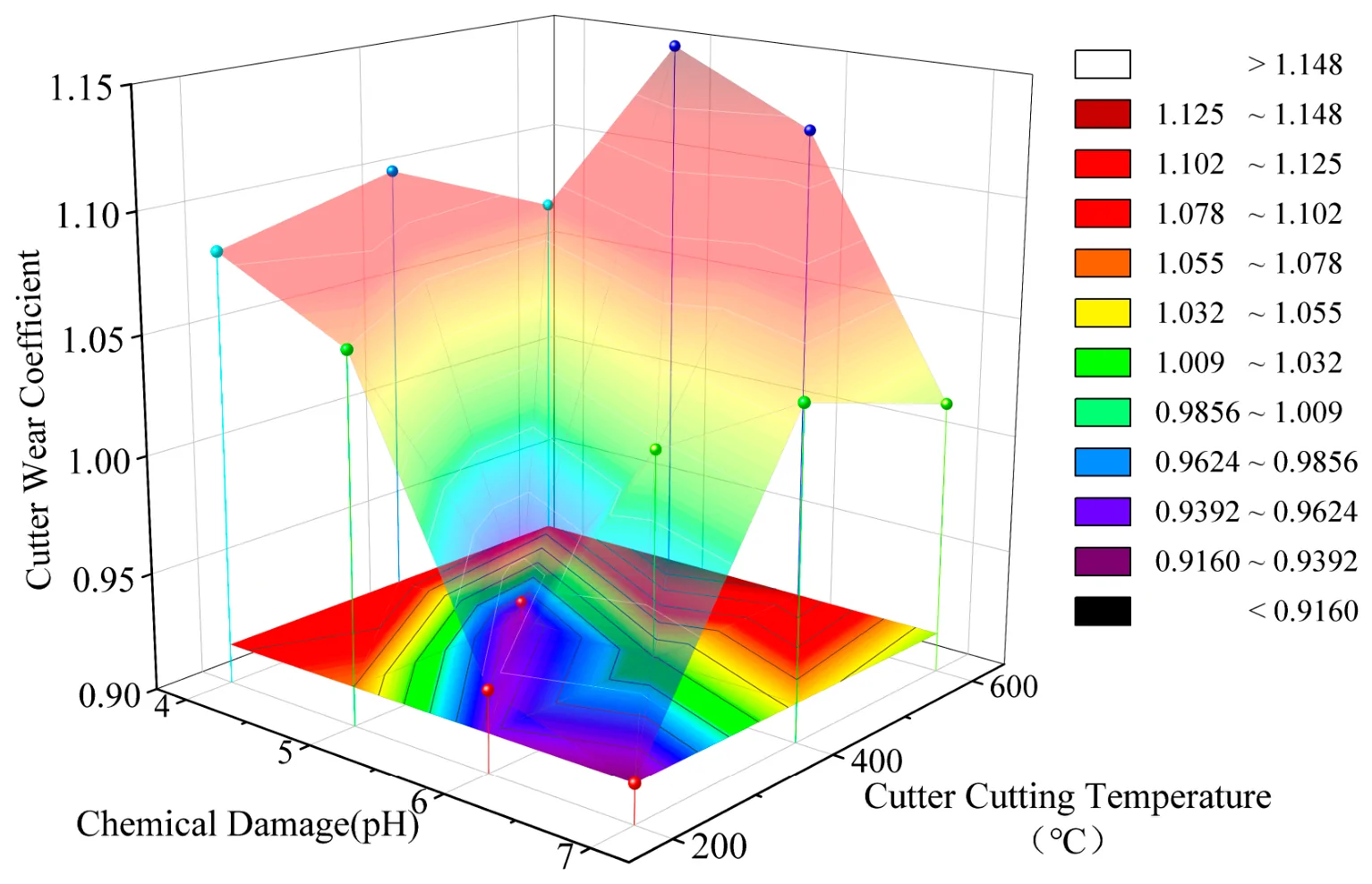
Wear coefficient response surface of single-cutter granite section under thermal–chemical coupling with respect to pH value and cutting temperature.

Figure 10 presents the corresponding response for the granite section. At 200 °C, wear coefficients range from 0.93 to 1.08, near or slightly above unity. At 400 °C, coefficients under pH 4 to 6 fluctuate between 0.92 and 1.10 with limited variation, and the pH 5 condition reaches 0.92 as the minimum. At 600 °C, coefficients range from 1.02 to 1.15, all marginally above unity.

At pH 5 and 400 °C, moderate acidity promotes infilling of altered products within microfractures, thereby reducing surface roughness and producing the minimum coefficient. The quartz α–β-phase transition in granite is constrained by the lattice structures of feldspar and mica, with thermal expansion stresses buffered through multi-mineral cooperative deformation, restricting microcrack development and resulting in a gradual abrasiveness increment. The high mineral hardness and dense lattice structure of granite effectively suppress temperature–chemical dual-field coupling damage, ensuring superior service stability of cutters in granite sections compared to sandstone sections.

**Figure 11:**
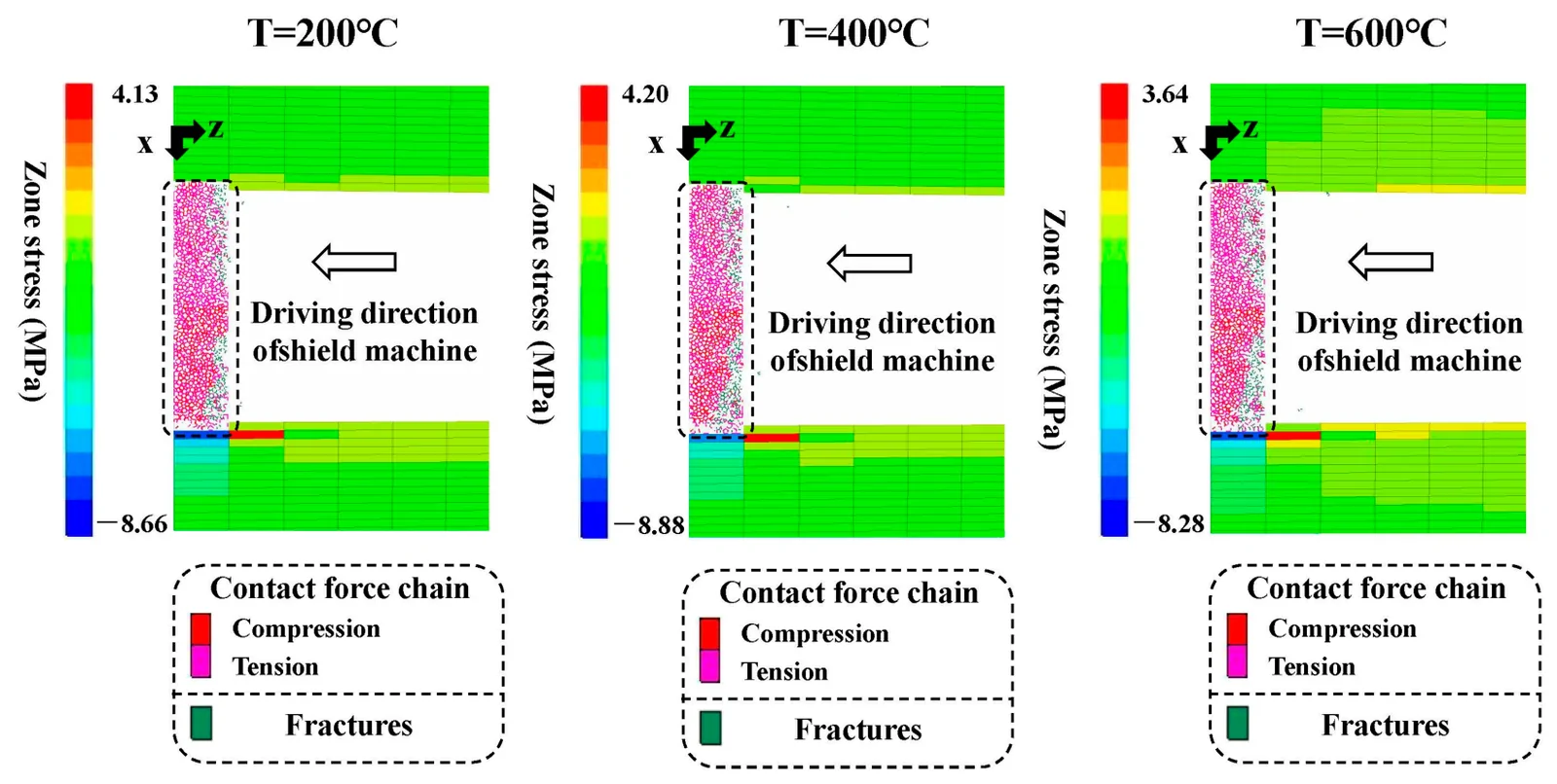
Contact force chain and crack distribution in composite strata under different cutting temperatures at pH 4.

**Figure 12:**
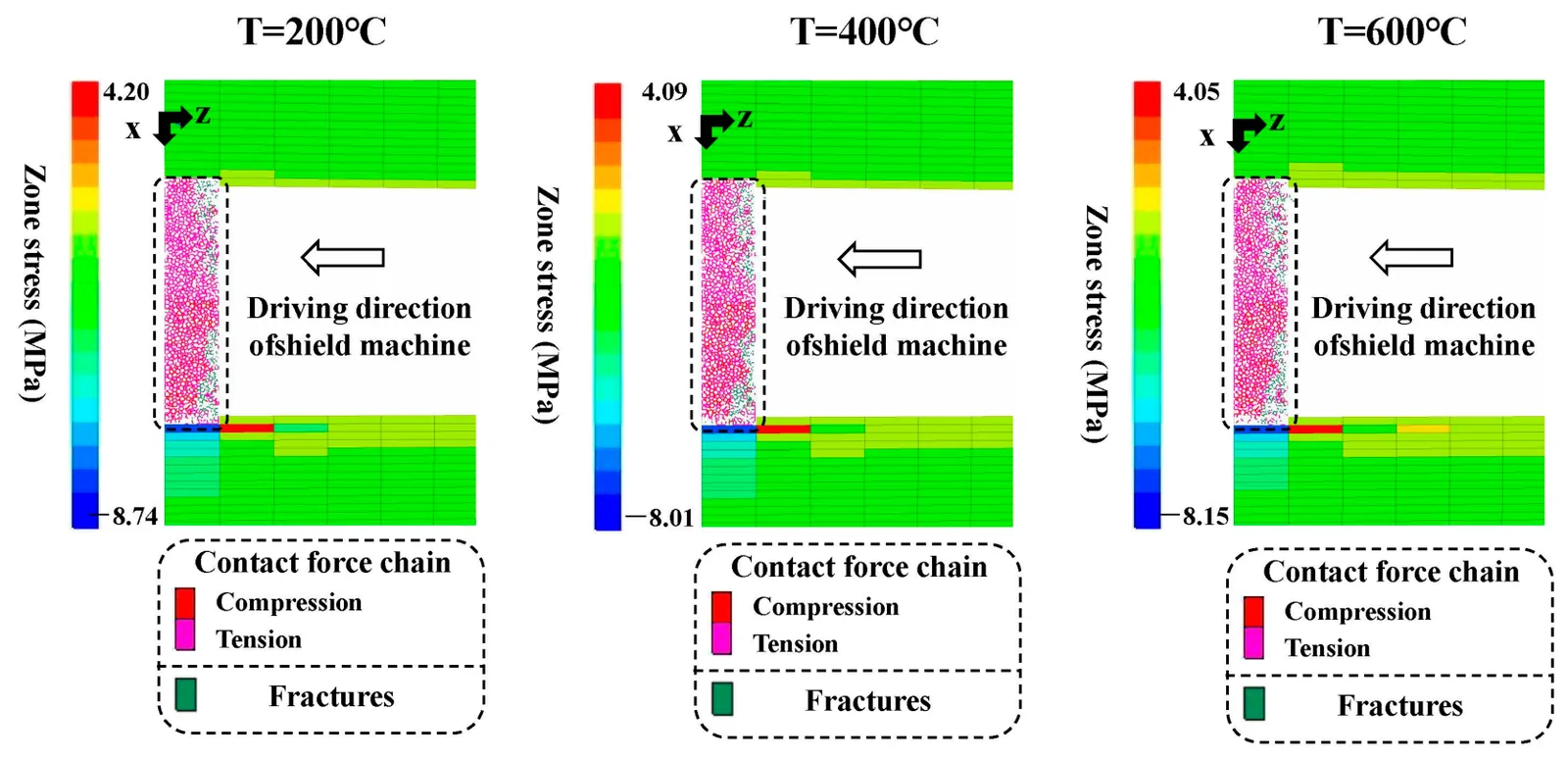
Contact force chain and crack distribution in composite strata under different cutting temperatures at pH 7.

As shown in Figure 11 and Figure 12, at pH 4, the upper sandstone section exhibits dense tensile components in contact force chains with well-developed microcracks, leading to fine-grained fragmentation and elevated wear coefficients ranging from 0.91 to 1.42; the lower granite section shows sparse contact force chains with large-scale rock spalling, yielding wear coefficients of 1.07 to 1.10. At pH 7, tensile components in the upper sandstone section weaken, fragmentation grain size increases, and wear coefficients decrease to 0.81–1.06; the lower granite section maintains stable contact force chain distribution with minimal wear coefficient fluctuation between 0.92 and 1.04. In the upper sandstone, acidic environments induce dense tensile contact force chains and well-developed microcracks, where fine-grained fragmentation intensifies cutter wear; neutral environments weaken tensile components and enlarge fragmentation grain sizes, thereby reducing wear. In the lower granite, sparse and stable contact force chains with large-block spalling render wear characteristics weakly susceptible to chemical environmental perturbation. The temperature–chemical coupling effect manifests more prominently in the upper sandstone section.

**Figure 13:**
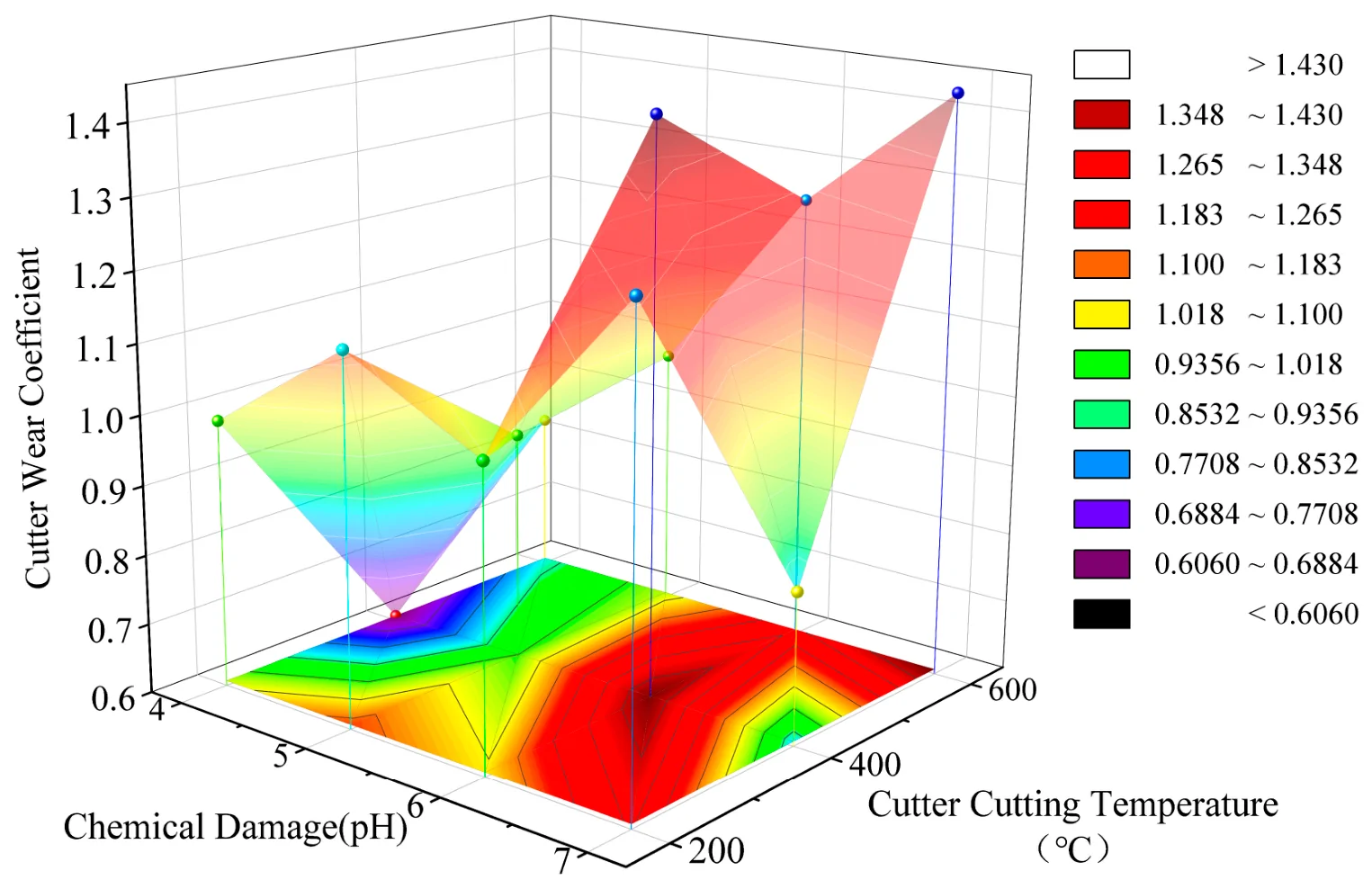
Wear coefficient response surface of single-cutter interface section under thermal–chemical coupling with respect to pH value and cutting temperature.

As shown in Figure 13, at 200 °C, interface wear is primarily attributed to weakly acidic chemical dissolution, with thermal–chemical coupling under pH 7 amplifying wear by 12.8%. At 400 °C, thermal expansion mismatch in neutral environments induces microfracture propagation, with pH 6 conditions amplifying wear by 41.8%. At 600 °C, cementitious dissolution at the interface intensifies, with pH 7 conditions exhibiting the most severe thermal–chemical coupling damage and wear coefficients approaching 1.43. Overall, interface wear shifts from chemical influence toward thermal–chemical coupling with increasing temperature, and neutral to weakly alkaline high-temperature conditions constitute the most unfavorable wear scenarios due to mineral thermal expansion differential and interfacial cementitious deterioration.

**Figure 14:**
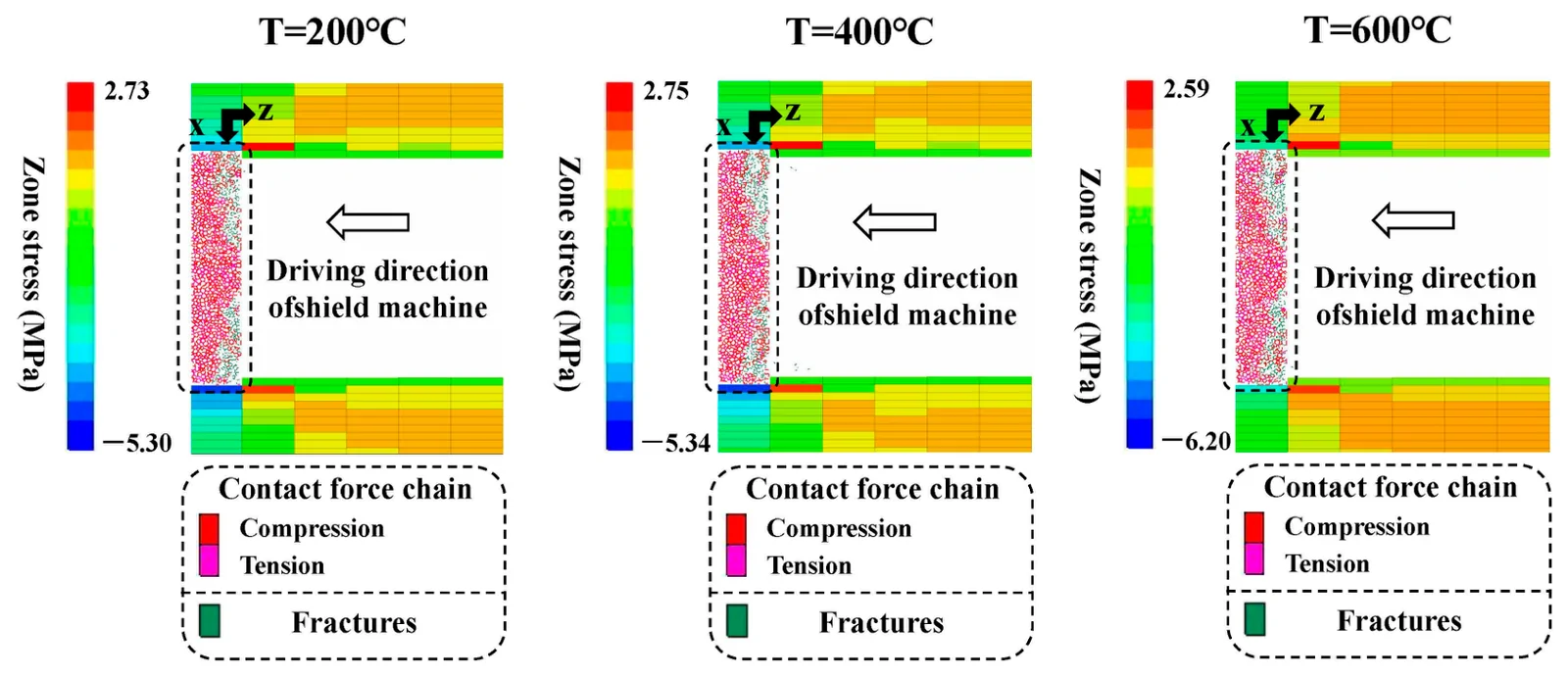
Contact force chain and crack distribution at interface section under different cutting temperatures at pH 4.

**Figure 15:**
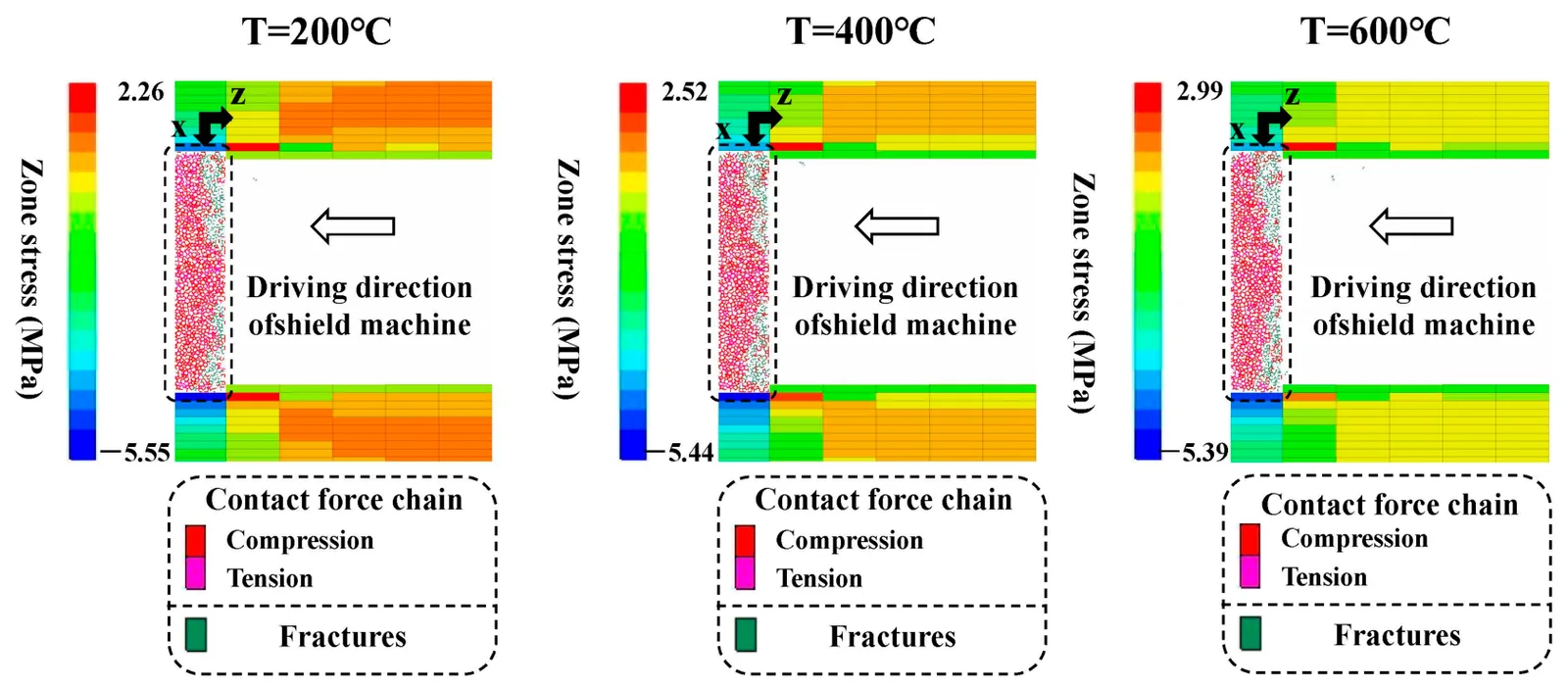
Contact force chain and crack distribution at interface section under different cutting temperatures at pH 7.

As shown in Figure 14 and Figure 15, at pH 4, chemical dissolution exerts a significant influence on interfacial cementitious weakening, and particle damage evolves from local spalling to throughgoing collapse with increasing temperature. At pH 7, chemical attack is suppressed; at 600 °C, mineral thermal expansion mismatch is superimposed on interfacial cementitious deterioration, interparticle engagement is progressively weakened, damage severity increases markedly, and wear coefficients approach 1.43. Neutral high-temperature conditions exhibit particularly severe particle damage due to significant thermal–chemical coupling effects.

Based on the preceding analysis of sandstone, granite, and interface sections, the coupled model suggests that differentiated temperature thresholds may reduce wear under the prescribed assumptions. Within the numerical framework, sandstone sections show wear coefficients below unity at 200 °C, yet exceeding unity at 400 °C and 600 °C, suggesting that active cooling systems could be considered to maintain the temperature below 200 °C for this lithology. Interface sections exhibit amplification, reaching 41.8% at 400 °C and coefficients approaching 1.43 at 600 °C, suggesting that cooling parameters might be optimized to reduce the high-temperature residence duration during penetration. Granite sections demonstrate coefficients near or marginally above unity across all tested temperatures, suggesting that conventional cooling may suffice without additional temperature control investment. This zoned strategy of strict control for sandstone, early warning for interface, and a relaxed standard for granite represents theoretical guidance derived from the model assumptions, and its field application requires validation through linear cutting machine tests or TBM field monitoring.

**Figure 16:**
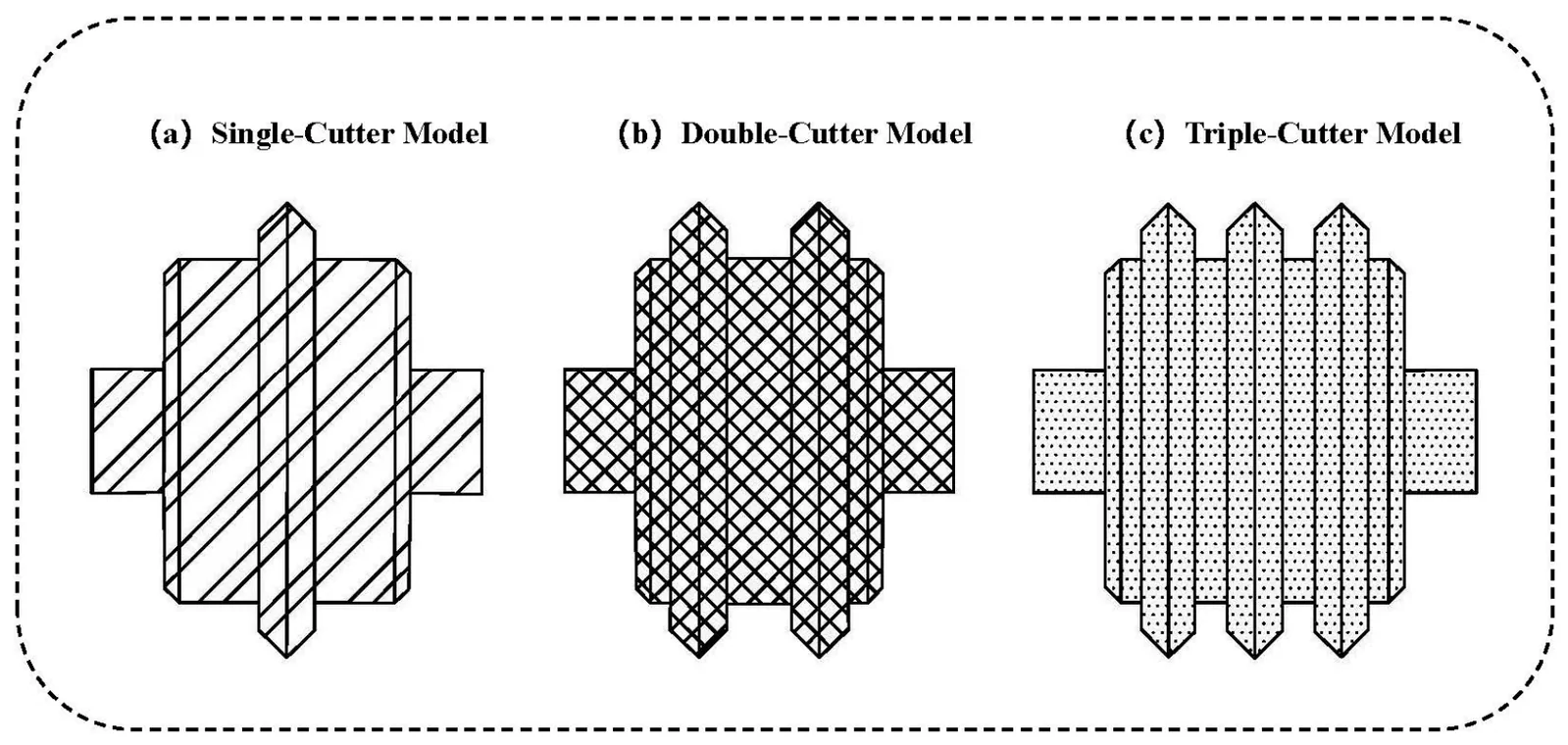
Schematic plan layout of single-, double-, and triple-cutter rock-breaking configurations.

Based on the single-cutter thermal–chemical coupling wear characteristics analysis, single-, double-, and triple-cutter rock-breaking models are further established, as shown in Figure 16, to systematically investigate total wear patterns under varying cutter numbers and arrangements, thereby exploring the feasibility of wear suppression in composite strata through cutter configuration optimization.

**Figure 17:**
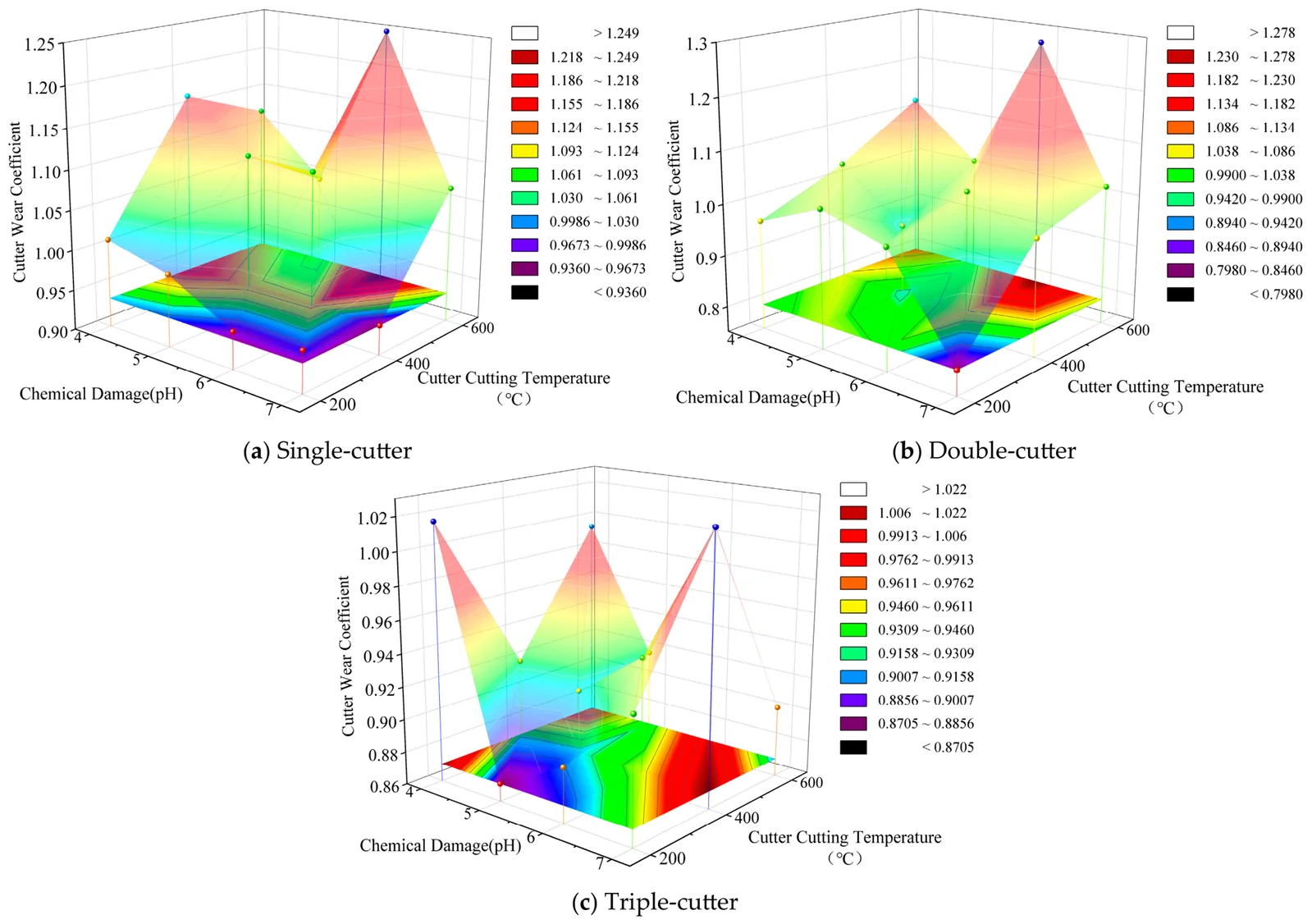
Comparison of wear coefficient response surfaces for single-, double-, and triple-cutter total wear under thermal–chemical coupling with respect to pH value and cutting temperature.

As shown in Figure 17, at 200 °C, wear coefficients of single-, double-, and triple-cutter configurations predominantly remain below unity. The double-cutter configuration under pH 7 achieves 0.798. This is the minimum across all conditions. It demonstrates a significant reduction through cooperative load sharing during rock breaking. The triple-cutter configuration exhibits wear that is comparable to single-cutter levels. Rock-breaking interference reduces individual cutter penetration depth and offsets the expected wear reduction benefit. At 400 °C, the single-cutter configuration under pH 4 reaches 1.16, the maximum across all conditions. Double- and triple-cutter configurations at 400 °C yield wear coefficients near unity because rock ridge secondary cutting and rock-breaking interference effects have not yet generated significant amplification. At 600 °C, the double-cutter configuration under pH 6 jumps to 1.28, the maximum across all conditions, as the grinding effect of rock ridge secondary cutting is sharply amplified under high temperatures; the single-cutter configuration under pH 6 reaches 1.25, and the triple-cutter configuration under pH 6 achieves 1.00, with triple-cutter interference dispersing stress concentration and returning wear patterns to single-cutter levels.

It follows that at 200 °C, the arrangement’s form has a pronounced influence on wear, with the double-cutter configuration yielding a significant reduction in wear. At 400 °C, single-cutter acidic conditions constitute the sensitive zone for abrupt wear increase, while double- and triple-cutter configurations exhibit relatively moderated wear due to cooperative effects. At 600 °C, the double-cutter configuration becomes dominant in wear due to rock-ridge secondary cutting, the triple-cutter arrangement achieves relative moderation through interference effects, and the single-cutter configuration remains at intermediate levels. Within the investigated parameter range, engineering practice may consider intensifying thermal management for double-cutter configurations above 600 °C and prioritizing temperature threshold monitoring for single-cutter configurations in acidic strata at 400 °C.

**Figure 18:**
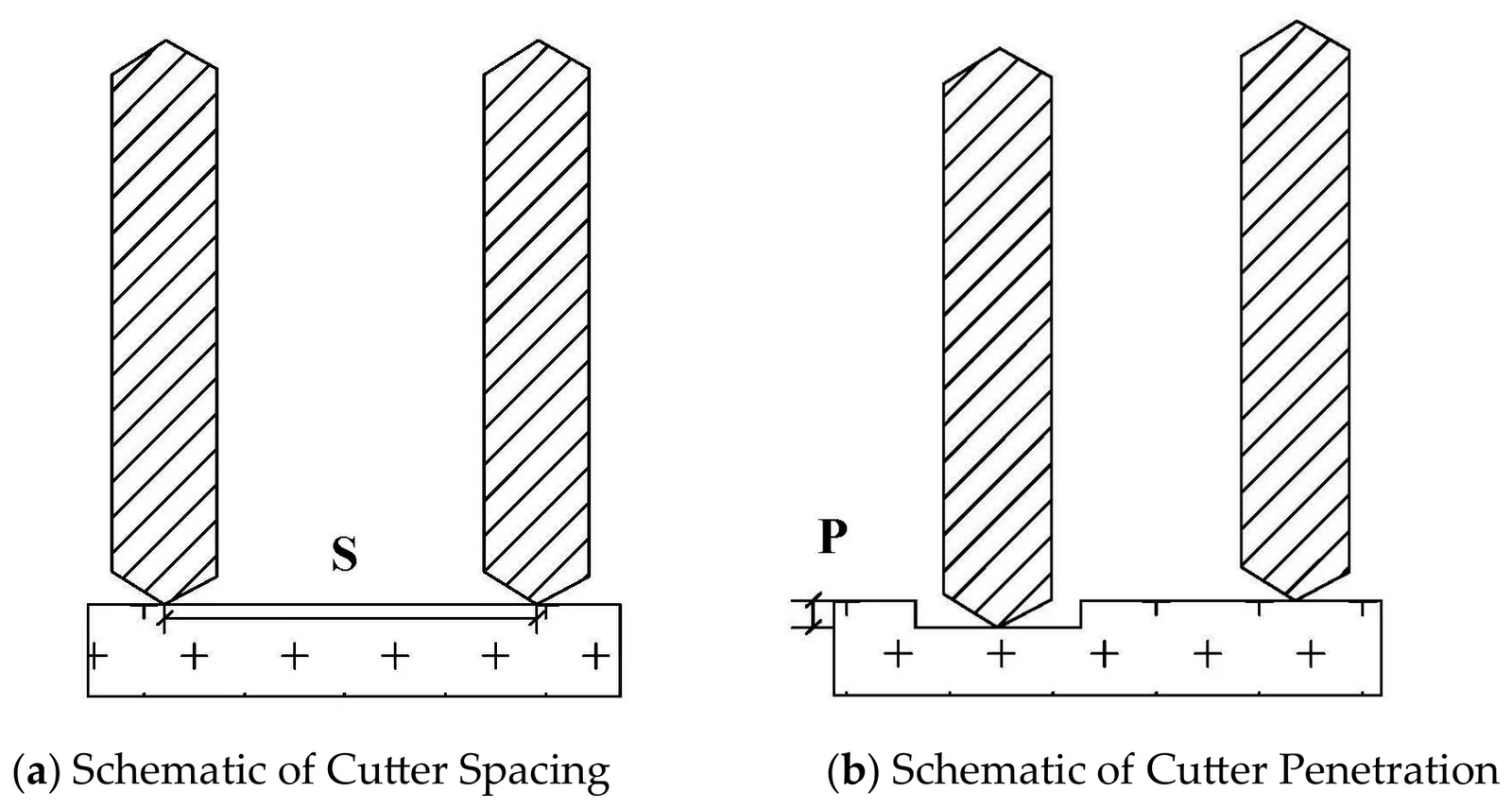
Geometric definition schematic of cutter spacing and penetration.

As shown in Figure 18, the numerical simulation represents different cutter spacings through the offset distance between cutters, and the penetration depth per revolution of a single cutter ring is defined as the cutter penetration. As shown in Table 4, to investigate the most unfavorable working conditions for cutter cutting in composite strata under 600 °C abnormal temperature and pH 6 weakly acidic environments, this study selects cutter penetrations of 5–7 mm/rev and cutter spacings of 60–90 mm for a full factorial combination design.

**Figure 19:**
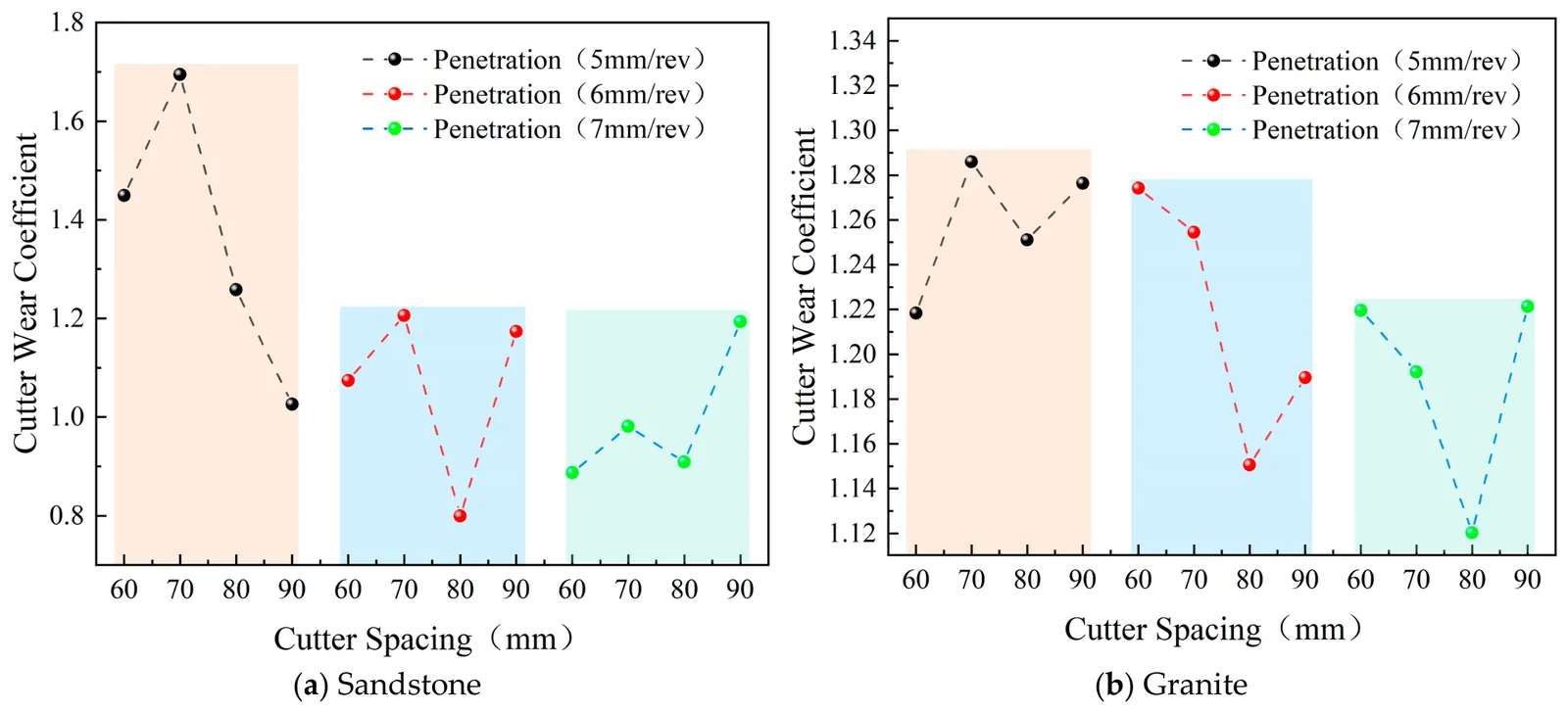
Variation of wear coefficients with cutter spacing and penetration for sandstone and granite sections under 600 °C and pH 6 conditions.

As shown in Figure 19, a wear peak occurs at a 70 mm cutter spacing across all penetration levels in the granite section. At 5 mm/rev penetration, the coefficient of 1.29 remains below the sandstone value of 1.69, as brittle fragmentation of hard rock produces fewer rock ridge remnants, resulting in weaker wear amplification than sandstone cementitious softening. At 6 mm/rev penetration, the value of 1.25 exceeds the sandstone value of 1.21, as elastic recovery of hard rock intensifies rock ridge rebound and aggravates secondary cutting. At 7 mm/rev penetration, the value of 1.22 still exceeds the sandstone value of 1.20, as sandstone achieves comprehensive wear reduction through volumetric fragmentation, while granite maintains stable rock ridges due to high hardness, preventing stress interference dilution by penetration depth.

The deep penetration response mechanisms of the two lithologies are fundamentally distinct: sandstone cementitious softening promotes volumetric fragmentation, while granite mineral hardness maintains rock ridge stability. Within the investigated parameter range, engineering practice may consider raising penetration to 7 mm/rev for comprehensive wear reduction in both sandstone and granite sections. The granite section should avoid the 70 mm cutter spacing critical interference zone under the current simulation conditions, and composite strata should implement zoned parameter optimization according to the lithological differences between the upper and lower sections.

**Figure 20:**
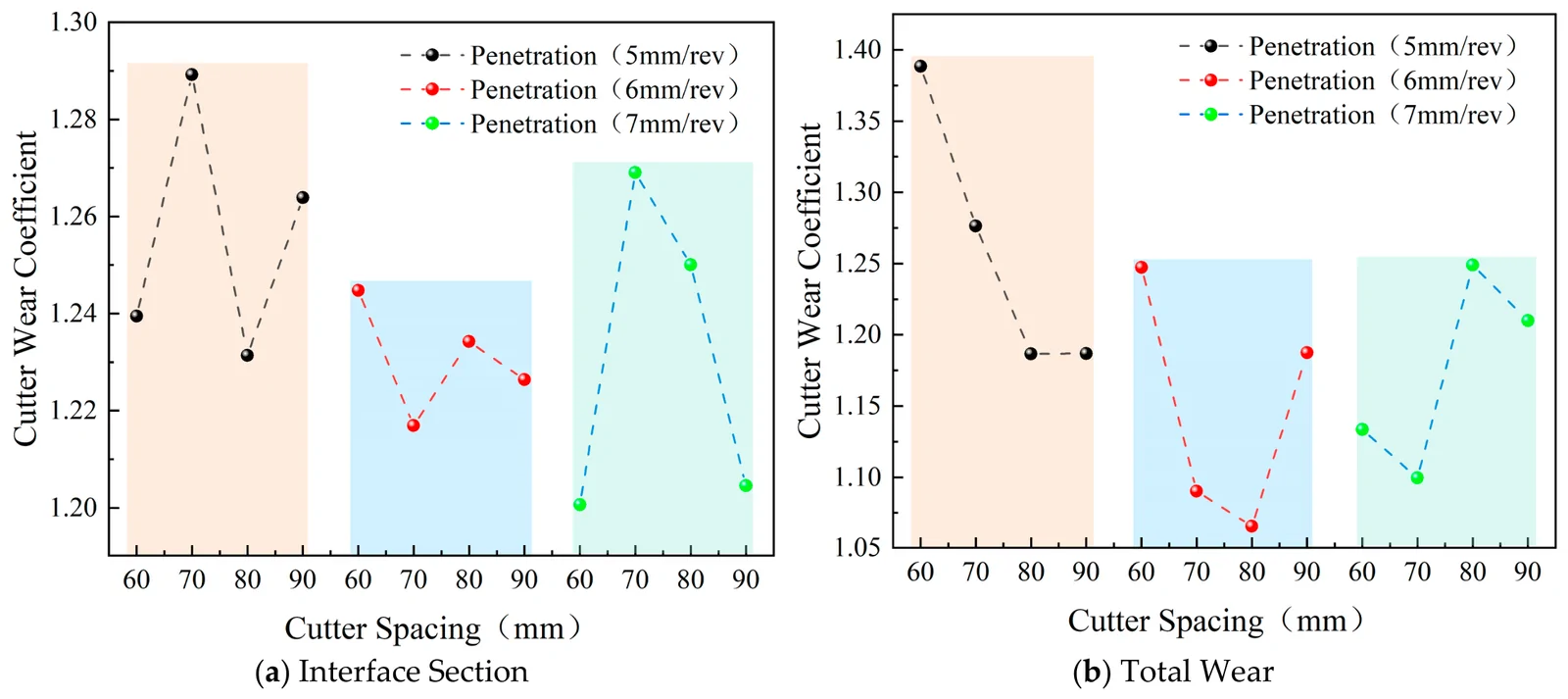
Variation of wear coefficients with cutter spacing and penetration for interface section and total wear under 600 °C and pH 6 conditions.

As shown in Figure 20, a wear peak is observed at a cutter spacing of 70 mm for most penetration levels at the interface section. At a penetration of 5 mm/rev, the coefficient reaches a maximum value of 1.29 across all conditions. This is attributed to the hardness differential between sandstone and granite, which induces interfacial stress concentration superimposed by rock ridge rebound, thereby rendering the critical interference effect more pronounced than that in monolithic rock. For total wear, the minimum of 1.07 occurs at a 6 mm/rev penetration and 80 mm cutter spacing, where multi-cutter cooperation and moderate penetration depth achieve balanced wear optimization across all three sections. At a penetration of 5 mm/rev, the total wear coefficient of 1.39 is significantly elevated by the abrupt increase in interface wear; however, the relatively lower wear in the sandstone and granite sections partially mitigates this amplification. Within the investigated parameter range and under the prescribed numerical assumptions, it is suggested that engineering practice should control the interface cutter spacing to avoid the critical interference zone at 70 mm. Furthermore, a penetration of 6 mm/rev combined with a cutter spacing of 70–80 mm is recommended as a favorable configuration to minimize total wear, with interface stability as the primary consideration.

### 4.3. Wear Characteristics of Composite Strata Under Cutter Cutting Thermal–Freeze–Thaw Damage Coupling

**Figure 21:**
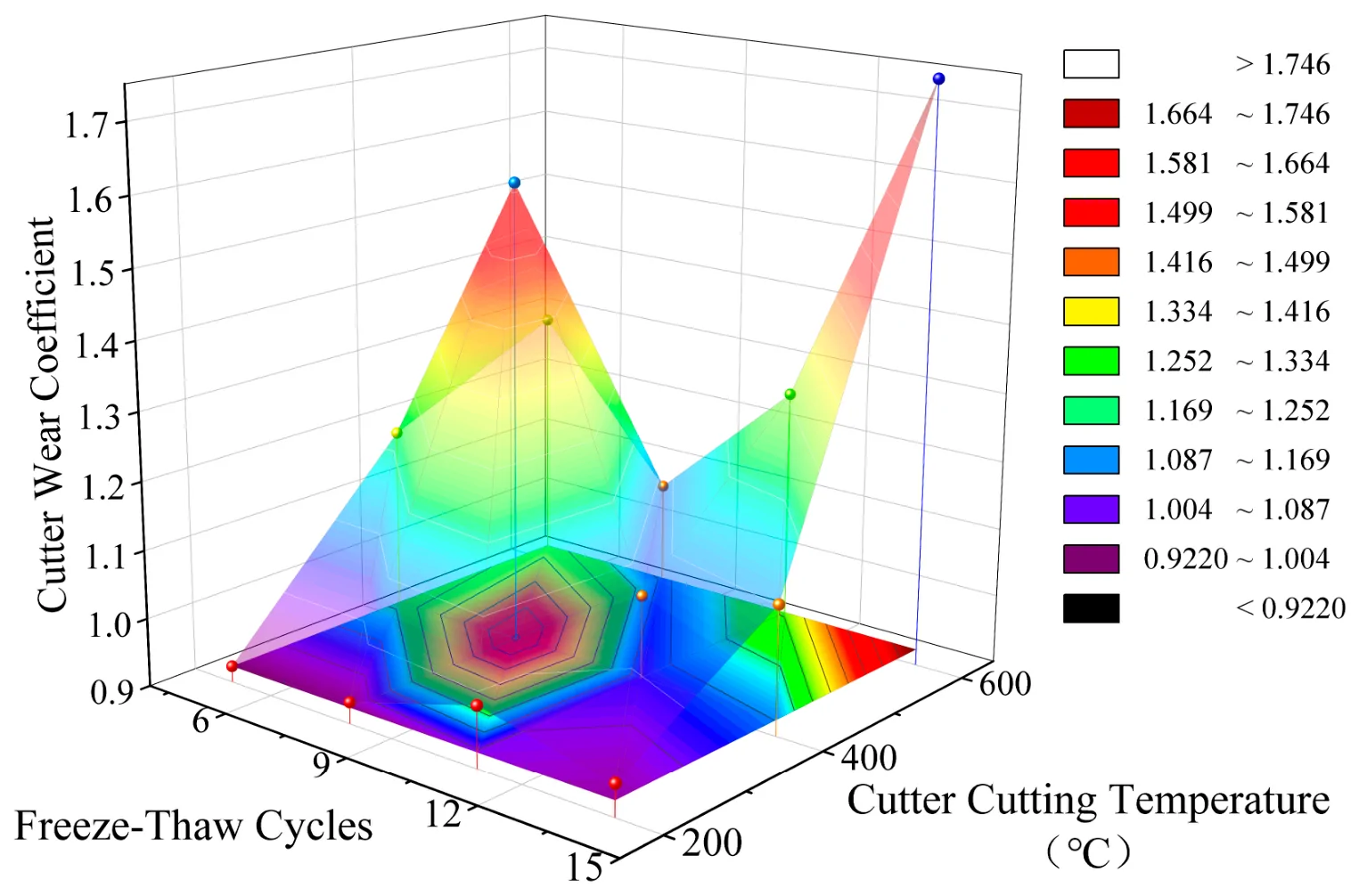
Wear coefficient response surface of single-cutter sandstone section under freeze–thaw–thermal coupling with respect to freeze–thaw cycles and cutting temperature.

As shown in Figure 21, at 200 °C, wear coefficients range from 0.92 to 0.99, all of which are below the baseline, indicating that freeze–thaw cycles do not significantly amplify sandstone wear and that mild thermal softening substantially reduces cutting resistance. At 400 °C, wear coefficients exhibit non-monotonic fluctuation, peaking at 1.59 under eight freeze–thaw cycles. This is attributed to thermal stress superimposed by frost heave stress, which induces throughgoing microcracks at cementitious interfaces. Consequently, the wear mechanism transitions from abrasive wear to debris spalling due to the weakening of rock mass structural planes. At 600 °C, wear coefficients monotonically increase from 1.29 to 1.75, reaching a maximum value of 1.75 under 14 freeze–thaw cycles across all tested conditions. The coupled temperature–freeze–thaw field significantly alters sandstone abrasiveness characteristics, with wear amplification reaching 59% at 400 °C under 8 freeze–thaw cycles and peaking at 75% at 600 °C under 14 freeze–thaw cycles.

**Figure 22:**
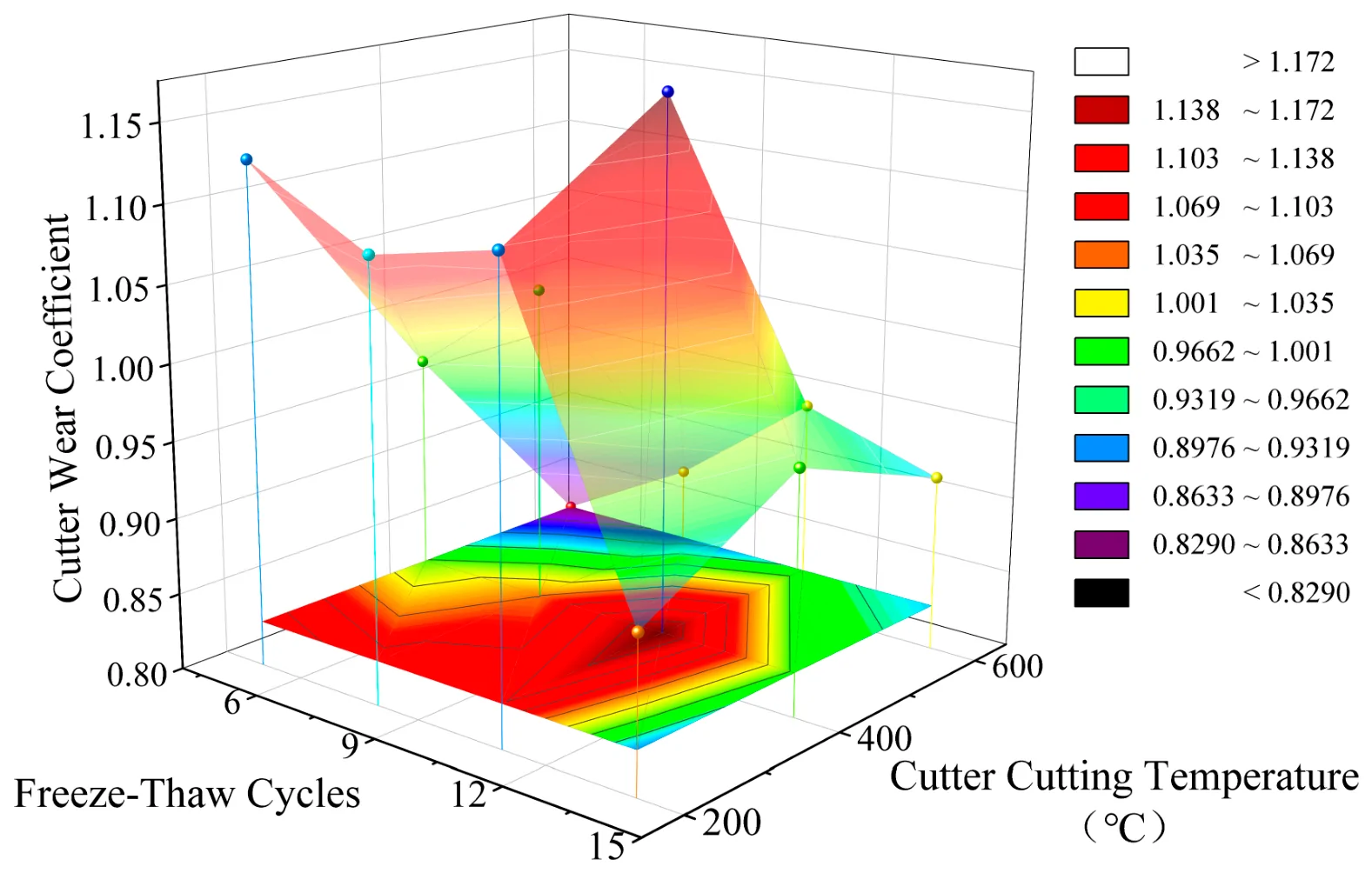
Wear coefficient response surface of single-cutter granite section under freeze–thaw–thermal coupling with respect to freeze–thaw cycles and cutting temperature.

As shown in Figure 22, at 200 °C, granite coefficients range from 1.08 to 1.13, all exceeding the baseline because the differential thermal expansion between quartz and feldspar induces intergranular cracking, thereby demonstrating diametrically opposite thermal response mechanisms between the two rock types. At 400 °C, sandstone wear peaks at 1.59, corresponding to a 59% amplification because throughgoing microcracks at cementitious interfaces trigger a wear mechanism transition. Granite wear peaks at 1.17, corresponding to a 17% amplification because either insufficient or excessive freeze–thaw cycles induce brittle rock mass disintegration. The sensitivity of sandstone markedly exceeds that of granite. At 600 °C, sandstone reaches the maximum of 1.75 across all conditions under 14 freeze–thaw cycles, where quartz-phase transition synergizes with freeze–thaw splitting; granite coefficients range from 0.83 to 0.94, all below the baseline, as high-temperature densification causes cutting to be significantly influenced by brittle fractures, exhibiting inconsistent high-temperature evolutionary wear characteristics between the two rock types. Sandstone wear monotonically amplifies with temperature and freeze–thaw cycles, peaking at 75% under 600 °C and 14 freeze–thaw cycles; granite exhibits non-monotonic fluctuation, with a local peak of 17% at 400 °C and values below the baseline at 600 °C, indicating that the fundamental difference between cemented and crystalline structures leads to significant thermal–freeze–thaw response divergence, rendering cutter life in sandstone sections more severely constrained by coupling control.

**Figure 23:**
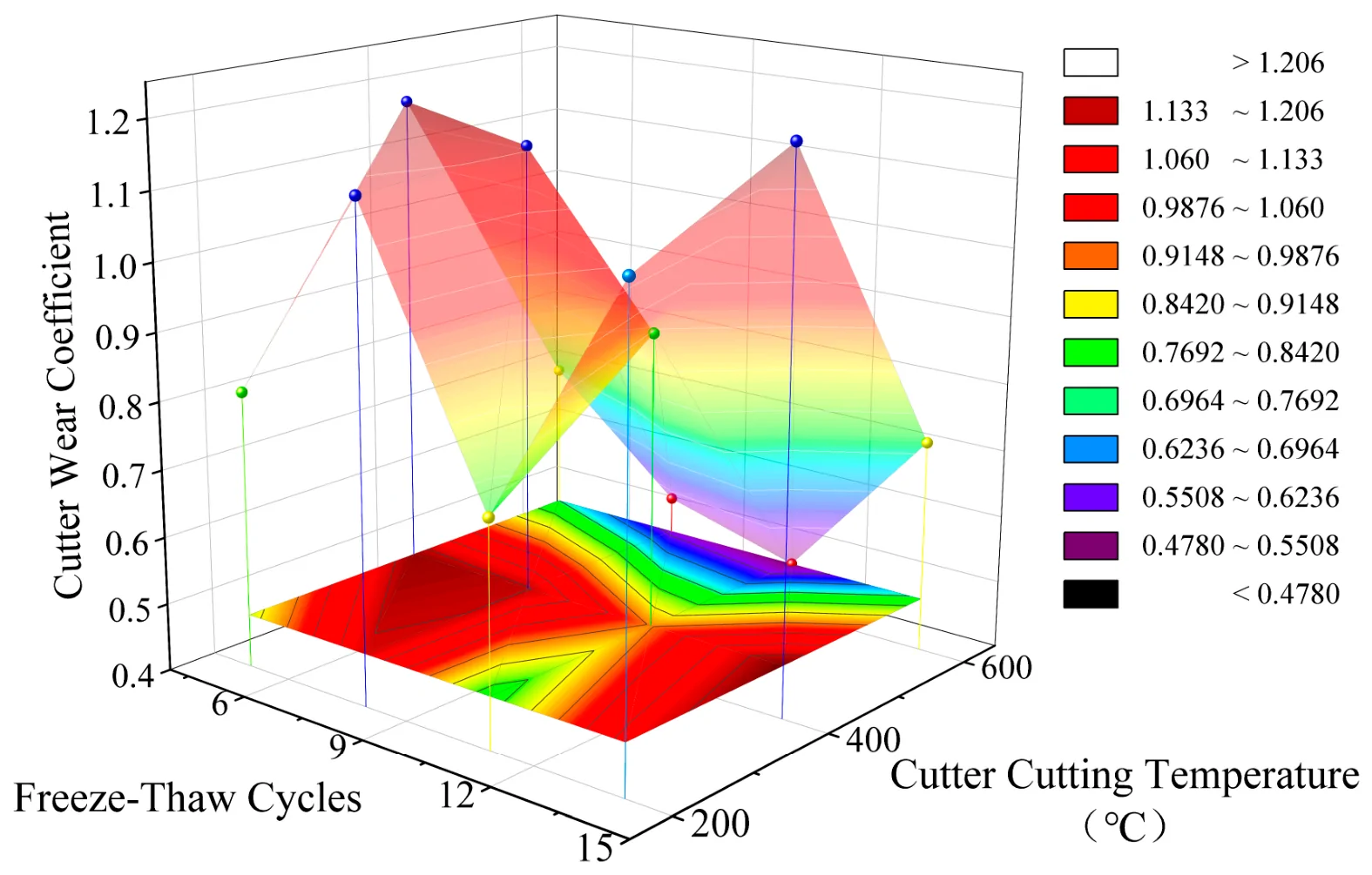
Wear coefficient response surface of single-cutter interface section under freeze–thaw–thermal coupling with respect to freeze–thaw cycles and cutting temperature.

As shown in Figure 23, at 200 °C, the wear coefficients range from 0.81 to 1.12; the value of 0.81 under 5 freeze–thaw cycles represents the minimum across all tested conditions, being 19% below the baseline, whereas the values of 1.12 and 1.08 under 8 and 14 freeze–thaw cycles, respectively, exceed the baseline. At low freeze–thaw cycle counts, interfacial fractures remain undeveloped and cutting resistance is low; as freeze–thaw cycles increase, the differential thermal expansion induces shear dislocation, freeze–thaw pore water propagates along fractures, and wear is amplified by 8–12%. At 400 °C, wear coefficients fluctuate between 0.91 and 1.20, peaking at 1.20 under 14 freeze–thaw cycles with 20% amplification; only under 11 freeze–thaw cycles does the value of 0.91 fall marginally below the baseline due to the transition of the rock mass disintegration mechanism. Interface wear generally exceeds the baseline across the 400 °C interval. At 600 °C, wear coefficients range from 0.48 to 0.72, all of which are significantly below the baseline. The minimum value of 0.48 under 11 freeze–thaw cycles corresponds to a 52% wear reduction.

In strata with freeze–thaw damage, cutter temperature regulation should be implemented through lithology-segmented strategies. Sandstone sections require strict low-temperature control, with 200 °C serving as the safety threshold. For granite and interface sections, either low-temperature excavation or high-temperature densification modes may be adopted, thereby avoiding the intermediate 400 °C high-wear interval. Temperature monitoring points should be deployed at the cutterhead center and peripheral cutter positions to provide real-time cutting temperature feedback to form closed-loop control linked with the cutterhead rotational speed and the cooling water flow rate, thereby ensuring that all lithology segments remain within temperature intervals where wear coefficients remain below unity or within controllable amplification ranges.

**Figure 24:**
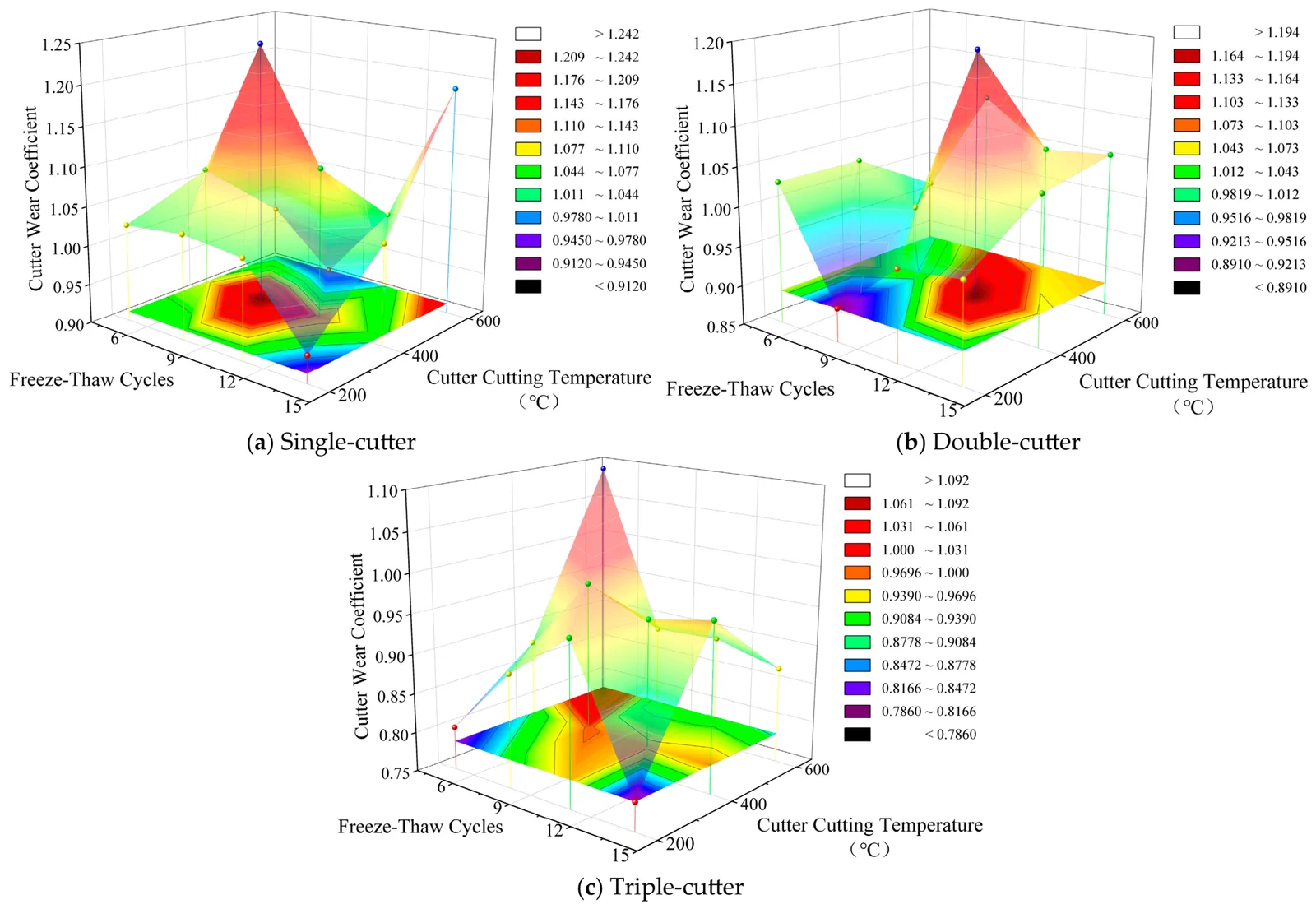
Comparison of wear coefficient response surfaces for single-, double-, and triple-cutter total wear under freeze–thaw–thermal coupling with respect to freeze–thaw cycles and cutting temperature.

As shown in Figure 24, within the 200 °C and 400 °C intervals, the wear of all three cutter configurations remains below the baseline, rendering single-, double-, and triple-cutter arrangements all applicable, with priority given to multi-cutter configurations for higher excavation efficiency. Within the 600 °C interval, wear control effects diverge significantly: the single-cutter amplifies wear by 30% under 11 freeze–thaw cycles, the double-cutter by 20% under 8 freeze–thaw cycles, and all four working conditions of the triple-cutter remain near the baseline. Triple-cutter dense arrangement suppresses thermal–freeze–thaw synergistic deterioration through dual mechanisms of load reduction and heat dissipation, constituting the optimal cutter configuration among the tested arrangements for high-temperature freeze–thaw strata under the current simulation conditions. Overall, in strata with freeze–thaw damage, cutter configuration selection within low-temperature intervals should be based on excavation efficiency, whereas the triple-cutter configuration should be prioritized within the 600 °C high-temperature intervals to avoid wear amplification through the load-reduction and heat-dissipation effects of dense arrangements.

**Figure 25:**
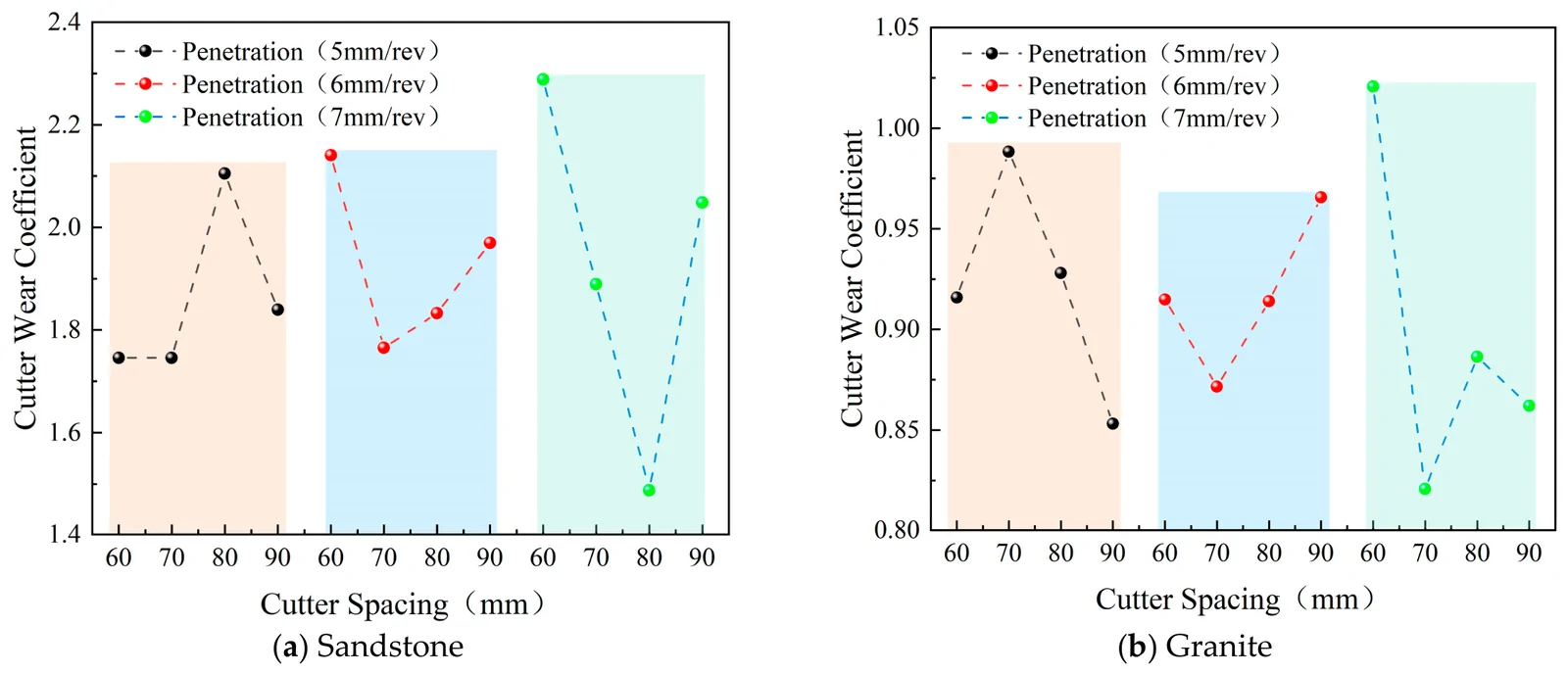
Variation in wear coefficients with cutter spacing and penetration for sandstone and granite sections under 600 °C and 14 freeze–thaw cycles.

As shown in Figure 25, sandstone wear coefficients range from 1.49 to 2.29, with a minimum value of 1.49 at a penetration of 7 mm/rev and a cutter spacing of 80 mm; granite coefficients range from 0.82 to 1.02, with a minimum value of 0.82 at a penetration of 7 mm/rev and a cutter spacing of 70 mm. For sandstone, large penetration with 80 mm cutter spacing achieves optimal load dispersion, while small penetration with 80 mm cutter spacing induces chip retention and conversely produces wear peaks. For granite, wear generally decreases with increasing penetration, and a cutter spacing of 70 mm optimally balances the degree of fracture zone overlap.

**Figure 26:**
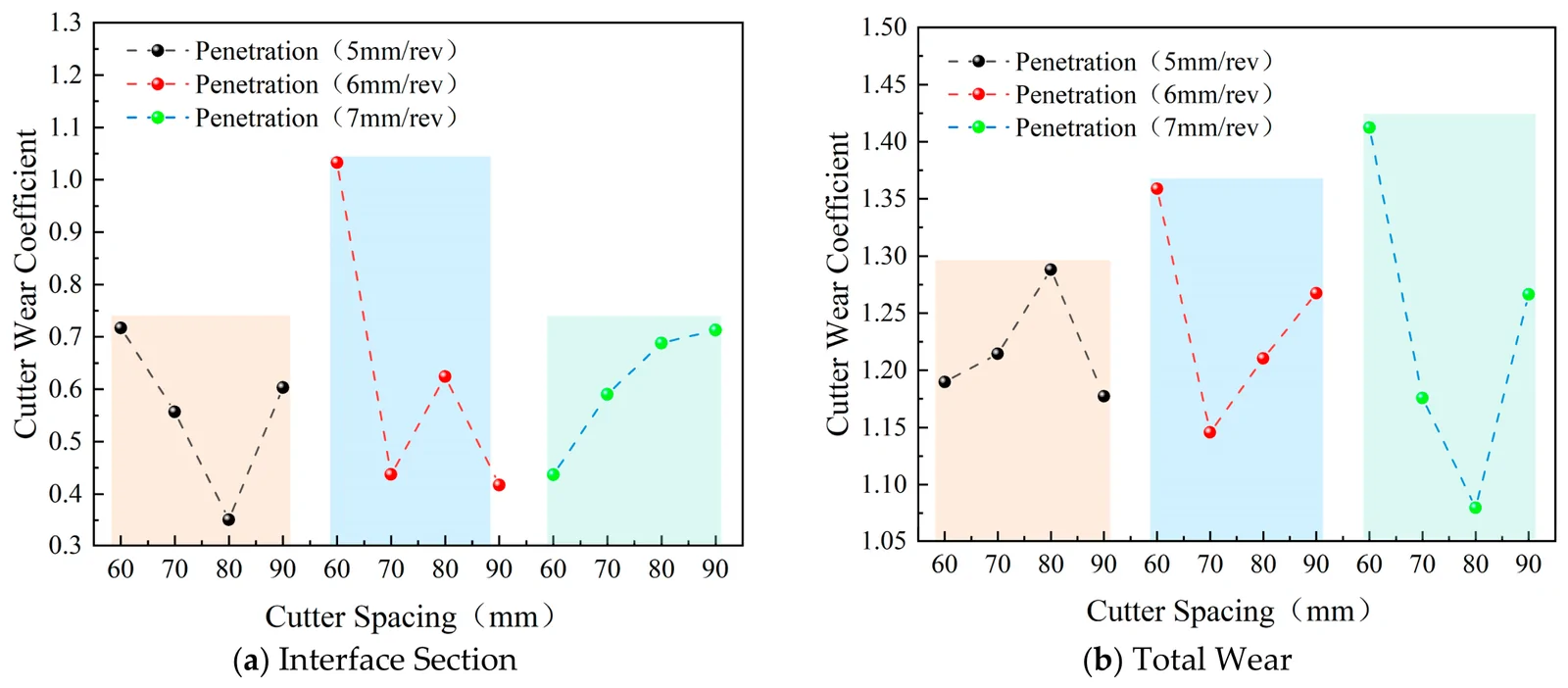
Variation in wear coefficients with cutter spacing and penetration for interface section and total wear under 600 °C and 14 freeze–thaw cycles.

As shown in Figure 26, interface wear ranges from 0.35 to 1.03, with a minimum value of 0.35 at a penetration of 5 mm/rev and a cutter spacing of 80 mm, and a maximum value of 1.03 at a penetration of 6 mm/rev and a cutter spacing of 60 mm. Total wear ranges from 1.08 to 1.41, with the minimum of 1.08 at 7 mm/rev penetration and 80 mm cutter spacing, and the maximum of 1.41 at 7 mm/rev penetration and 60 mm cutter spacing. Small penetration with large cutter spacing induces fracture angles spanning across the interface, causing an abrupt specific cutting energy reduction; small cutter spacing with simultaneous engagement of both hard and soft rock layers induces shear dislocation and abrupt wear increase. At 80 mm cutter spacing with large penetration, cooperative expansion of fracture zones across all three rock sections achieves optimal configuration, while small penetration conversely elevates wear due to chip retention in cutting blind zones.

## 5. Discussion

### 5.1. Sensitivity Analysis of Cutter Parameters Under Cutter Cutting Thermal–Chemical–Freeze–Thaw Multi-Damage Coupling

This study systematically investigates the coupling mechanism between environmental damage external factors and cutter parameters on wear under a 600 °C abnormal cutting temperature. Chemical erosion factors select pH values of 4, 5, 6, and 7, covering the chemical corrosion gradient from acidic groundwater environments to neutral water bodies. Freeze–thaw damage factors set cycle counts of 5, 8, 11, and 14, corresponding to annual damage accumulation effects over 1–3 years in seasonally frozen regions under a single-cycle mode of −8 °C to 25 °C. Cutter spacing selects 60, 70, 80, and 90 mm, encompassing the commonly used TBM cutter spacing range while accounting for stress field superposition and geometric optimization intervals for rock ridge fragmentation. Cutter penetration selects 5, 6, 7, and 8 mm/rev, matching typical hard rock excavation advance rates and covering the transition interval from low-penetration grinding to high-penetration fracture rock-breaking modes.

This orthogonal scheme reduces the test matrix to 16 groups through balanced dispersion and uniform comparability design principles, thereby ensuring statistical significance. The four-factor level settings derive from engineering actual monitoring data and existing literature value ranges, with boundary calibration conducted through preliminary numerical simulation pre-studies to ensure parameter combinations maintain physical realizability and engineering representativeness under abnormal high-temperature working conditions, providing an experimental framework for revealing primary–secondary effects and interaction mechanisms of multi-factor coupling on cutter wear. Table 5, Table 6 and Table 7 present wear values at different cutting locations under various cutter and combination configurations.

### 5.2. Sensitivity Correlation Analysis of Cutter Parameters Under Cutter Cutting Thermal–Chemical–Freeze–Thaw Multi-Damage Coupling

**Figure 27:**
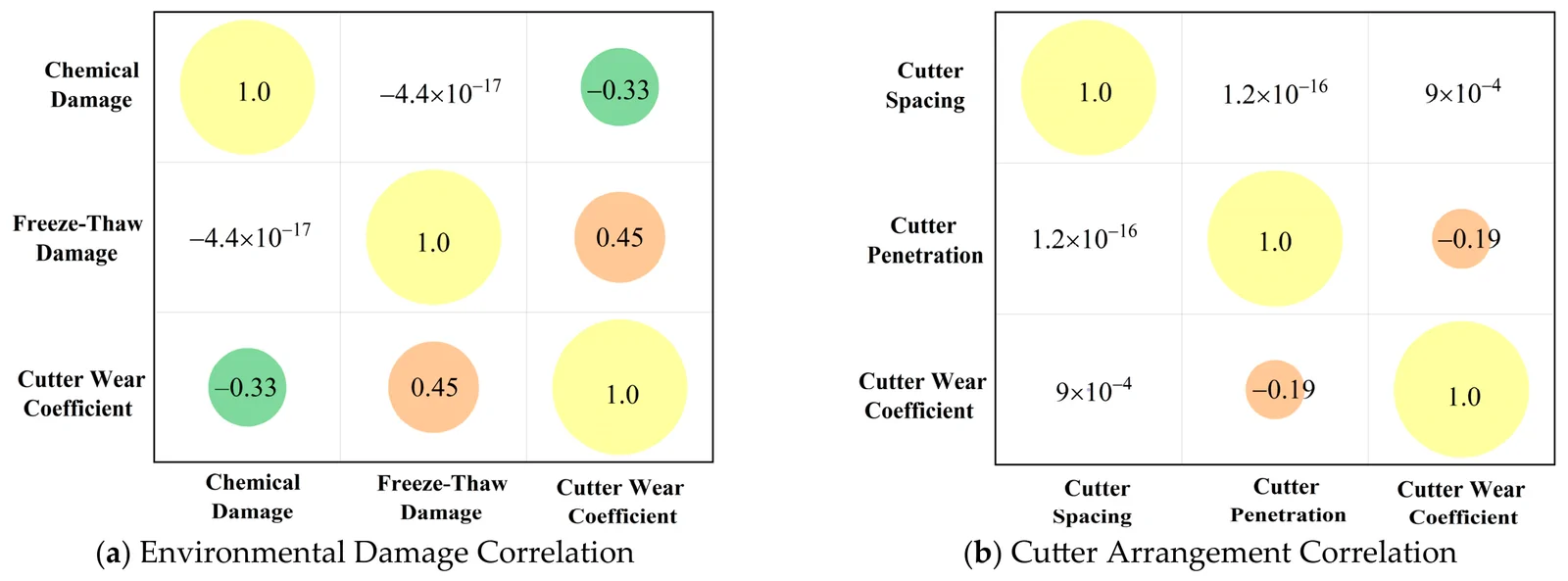
Pearson correlation matrix of single-cutter FT–T–C coupling sandstone section wear coefficient with environmental damage and cutter parameters.

As shown in Figure 27, the correlation analysis is presented as a bubble chart, where circle size indicates the absolute correlation value and different colors are used solely to distinguish between factors. For single-cutter sandstone section cutting, freeze–thaw damage shows the highest correlation coefficient at 0.45 among all factors. The combined environmental damage external factors amount to 0.78, substantially exceeding the cutter configuration internal factors at 0.19. At this stage, rock chip secondary crushing effects have not yet developed. Chemical damage at −0.33 simultaneously weakens rock mass strength. Environmental deterioration thus emerges as the primary wear influence.

**Figure 28:**
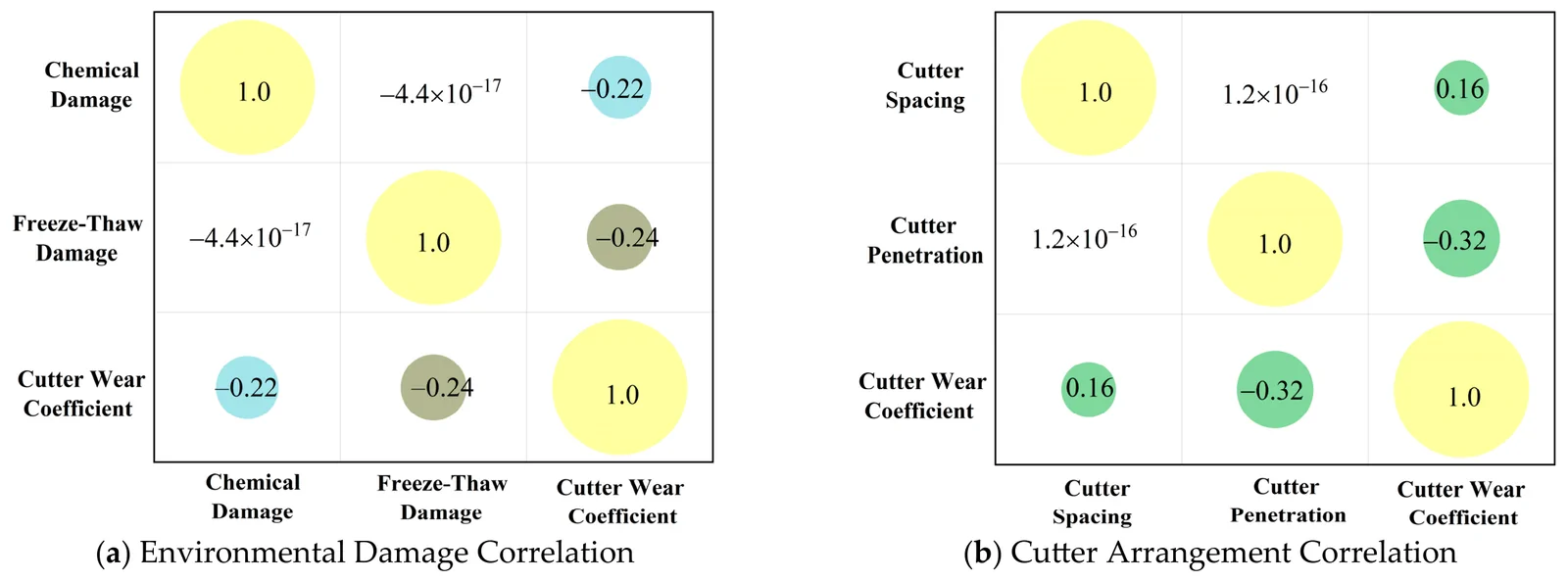
Pearson correlation matrix of double-cutter FT–T–C coupling sandstone section wear coefficient with environmental damage and cutter parameters.

As shown in Figure 28, for double-cutter sandstone section cutting, the correlation coefficient of cutter penetration reaches −0.32, the largest absolute value among the four factors, while the cutter configuration internal factors total 0.48, approaching the environmental damage external factors at 0.46. The internal and external factor groups exhibit comparable influence magnitudes. Double-tooth cooperation introduces inter-tooth stress field coupling, where rock ridge fragmentation complexity reduces the linear explanatory power of environmental deterioration. Large penetration drives rock breaking transformation from grinding to fracture, and the penetration regulation effect exceeds that of cutter spacing by approximately a factor of two.

**Figure 29:**
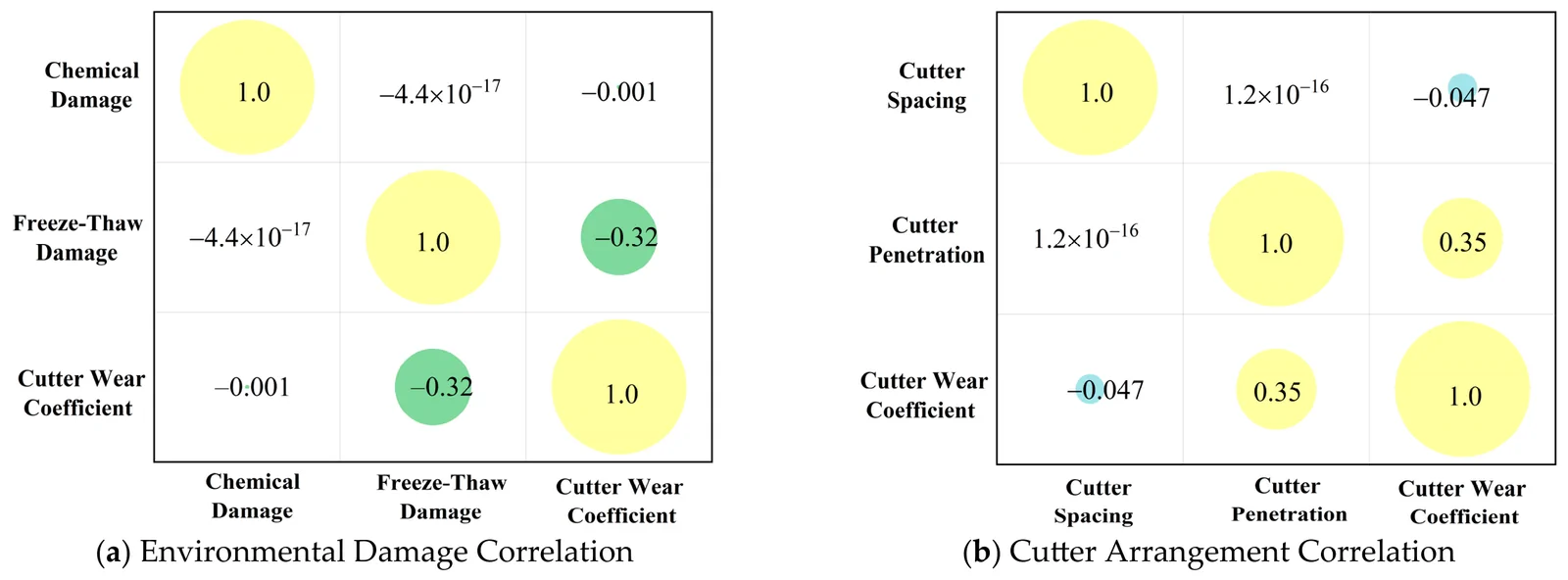
Pearson correlation matrix of triple-cutter FT–T–C coupling sandstone section wear coefficient with environmental damage and cutter parameters.

As shown in Figure 29, for the triple-cutter sandstone section cutting, cutter penetration at 0.35 further amplifies to the highest among all factors, followed by freeze–thaw damage at 0.32, while chemical damage at −0.001 and cutter spacing at −0.047 are both approximately irrelevant. Triple-tooth cooperation induces multiple rock chip crushing between cutters with extended retention duration, where fine particle repeated grinding causes cumulative wear amplification; multi-tooth stress field coupling completely overrides cutter spacing regulation of fracture zone overlap, and sharply increased rock ridge fragmentation complexity renders penetration as the primary factor while the chemical effects are masked by rock chip secondary crushing.

**Figure 30:**
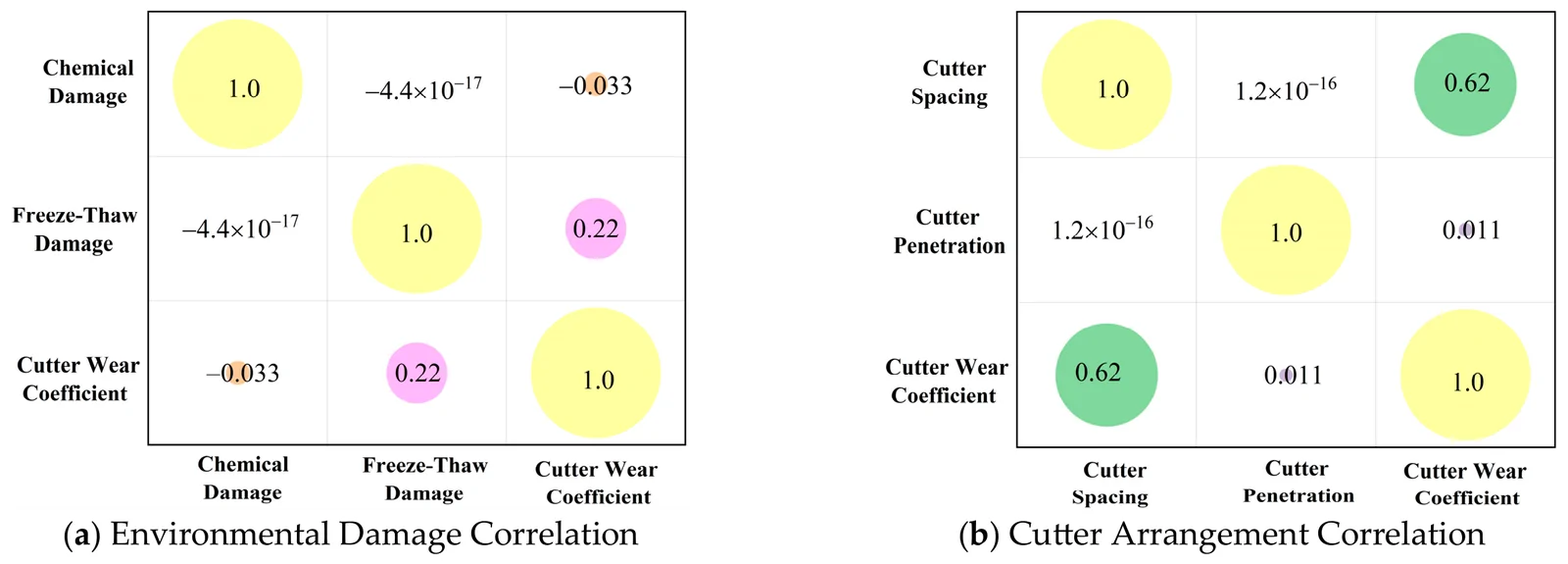
Pearson correlation matrix of single-cutter FT–T–C coupling granite section wear coefficient with environmental damage and cutter parameters.

As shown in Figure 30, for single-cutter granite section cutting, cutter spacing at 0.62 ranks highest among all factors, with cutter configuration internal factors totaling 0.63, far exceeding the environmental damage external factors totaling 0.25. The dense structure of granite restricts acidic dissolution propagation along grain boundaries, rendering chemical damage at −0.033 approximately irrelevant; low porosity limits freeze–thaw deterioration, yielding freeze–thaw damage at 0.22 as weakly positively correlated. Large cutter spacing increases rock ridge remnants and surges cutter–rock contact frequency, where high-hardness granite abrasiveness exerts a substantial influence on wear through cutting. Large penetration deep-cutting fracture effects are offset by stable brittle fracture characteristics of high-hardness rock mass, rendering penetration at 0.011 approximately irrelevant.

**Figure 31:**
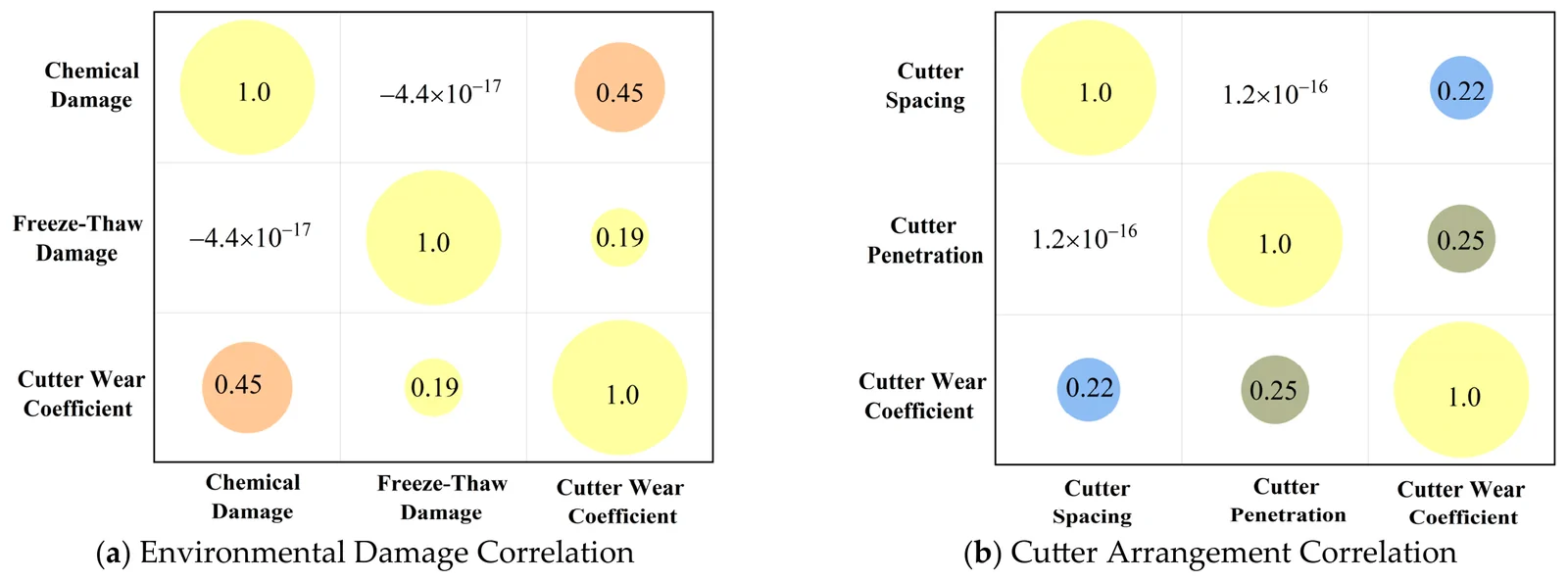
Pearson correlation matrix of double-cutter FT–T–C coupling granite section wear coefficient with environmental damage and cutter parameters.

As shown in Figure 31, for double-cutter granite section cutting, chemical damage at 0.45 exhibits the highest value among all factors, with the positive–negative correlation completely reversed from the −0.22 in sandstone sections. The environmental damage external factors total 0.64 and exceed the cutter configuration internal factors at 0.47. High quartz content in granite enables acidic dissolution propagation along grain boundaries, where mineral dissociation and surface roughening enhance abrasive grain wear; freeze–thaw effects weaken to 0.19 due to low porosity. Cutter spacing at 0.22 and penetration at 0.25 are both positively correlated, as large penetration under high hardness induces high-frequency collisions, and brittle-fracture rock chip sharp edges aggravate cutter ring abrasion.

**Figure 32:**
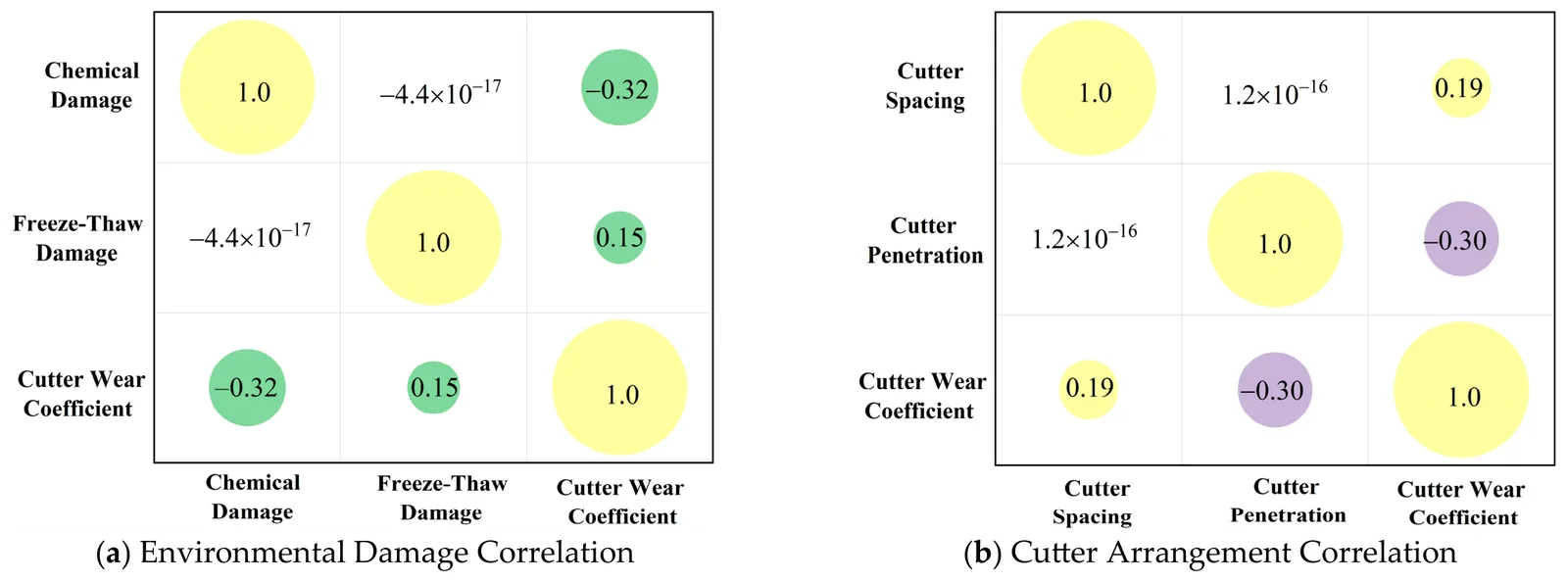
Pearson correlation matrix of triple-cutter FT–T–C coupling granite section wear coefficient with environmental damage and cutter parameters.

As shown in Figure 32, for triple-cutter granite section cutting, chemical damage at −0.32 and penetration at −0.30 constitute dual primary controls, with the environmental damage external factors totaling 0.47, approaching the cutter configuration internal factors that total 0.49 in magnitude. Under triple-tooth cooperation, enhanced acidity weakens the granite grain boundaries and induces mineral dissociation, yet high-hardness rock mass brittle-fracture characteristics partially mask the dissolution effects through inter-tooth stress field coupling; large-penetration deep-cutting drives rock breaking toward fracture transformation, with fracture-specific energy remaining higher than sandstone and partial wear reduction effects being retained. Freeze–thaw damage at 0.15 is weakly positively correlated, as low-porosity granite exhibits strong resistance to freeze–thaw deterioration.

**Figure 33:**
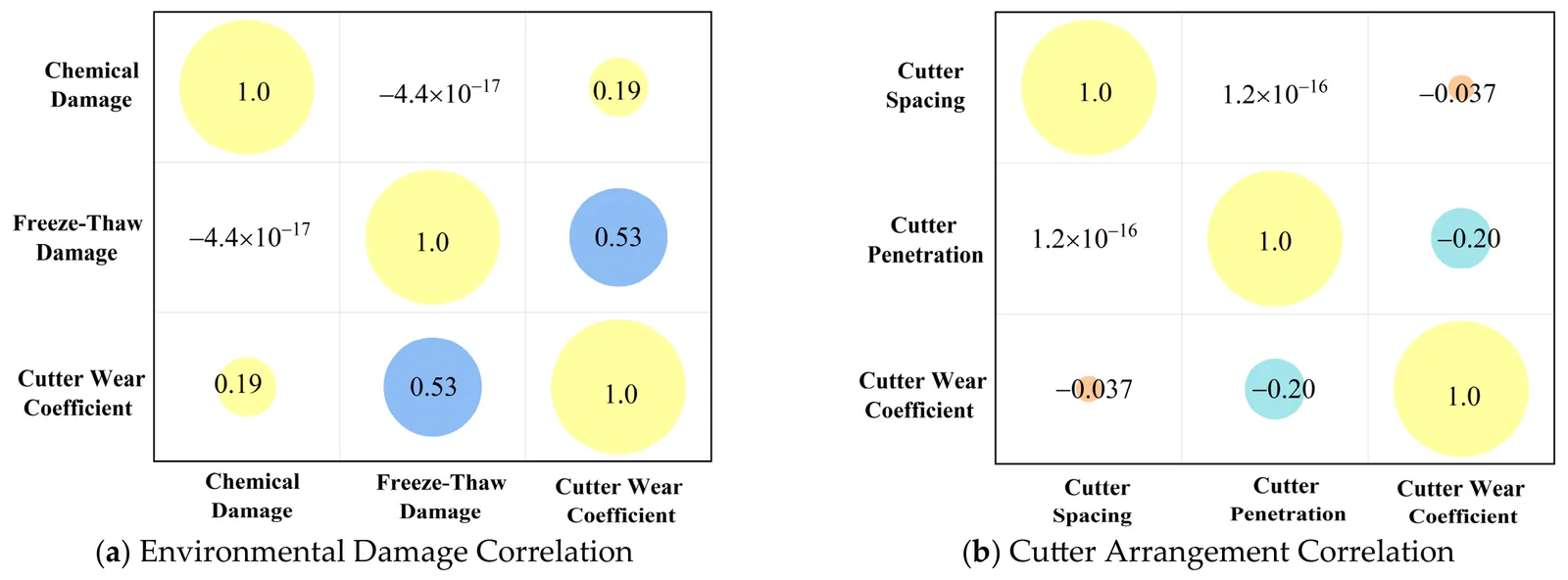
Pearson correlation matrix of single-cutter FT–T–C coupling interface section wear coefficient with environmental damage and cutter parameters.

As shown in Figure 33, for single-cutter interface section cutting, freeze–thaw damage at 0.53 ranks highest among all factors, with the environmental damage external factors totaling 0.72 far exceeding the cutter configuration internal factors totaling 0.24. Chemical damage at 0.19 is weakly positively correlated, as acidic dissolution propagates along sandstone–granite cementation weak zones, yet its effect remains inferior to freeze–thaw physical damage. Cutter spacing at −0.037 is approximately irrelevant, as lithological mutations render the regulation of fracture-zone geometric morphology by cutter spacing overridden by interface stress fields.

**Figure 34:**
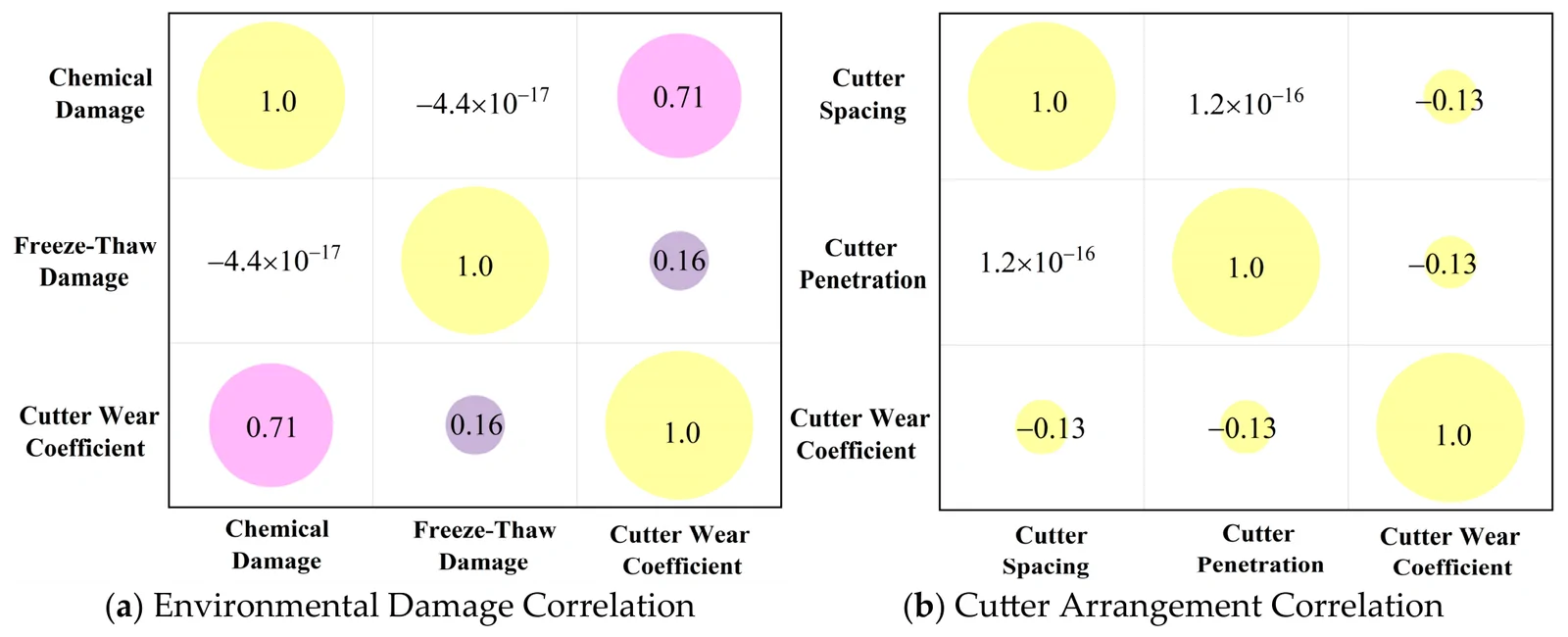
Pearson correlation matrix of double-cutter FT–T–C coupling interface section wear coefficient with environmental damage and cutter parameters.

As shown in Figure 34, for double-cutter interface section cutting, chemical damage at 0.71 exhibits the highest value across all three sections. The environmental damage external factors total 0.87 and exceed the cutter internal factors at 0.26. Freeze–thaw effects weaken to 0.16 as interface stress concentration restricts crack propagation. Cutter spacing at −0.13 and penetration at −0.13 share identical absolute values and rank lowest among the four factors, as lithological mutations at the interface significantly weaken the cutter’s parameter regulation capacity.

**Figure 35:**
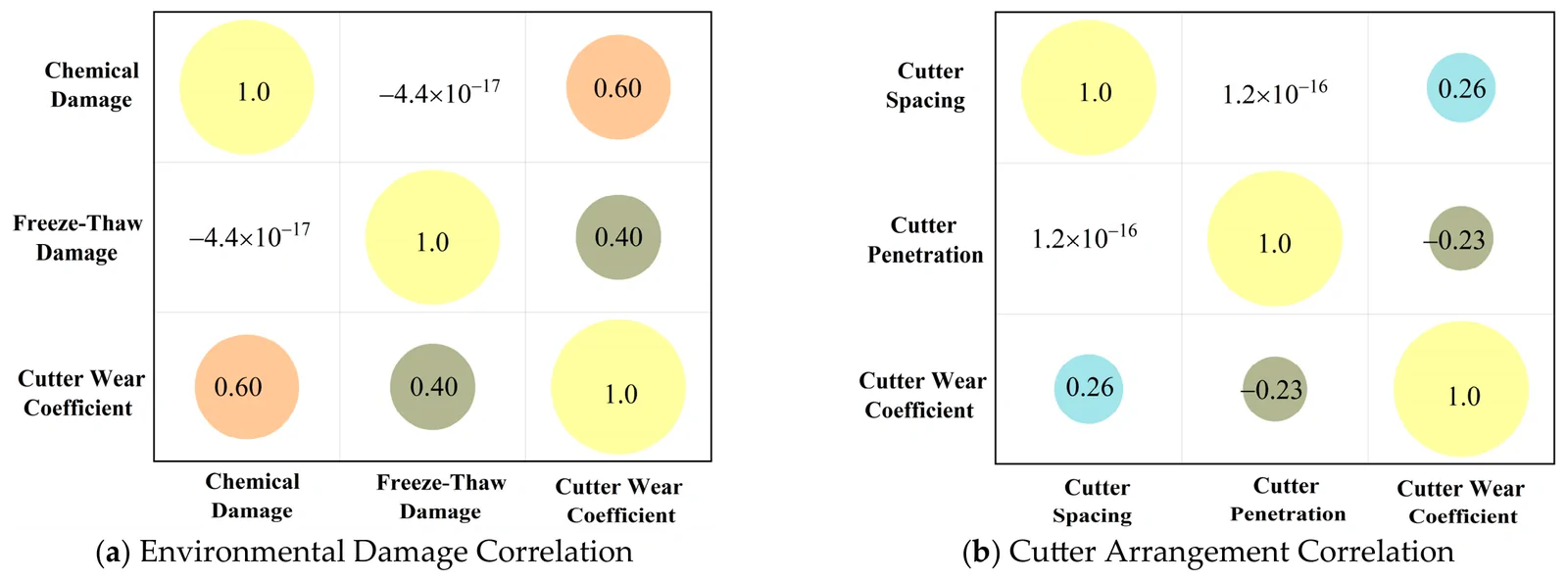
Pearson correlation matrix of triple-cutter FT–T–C coupling interface section wear coefficient with environmental damage and cutter parameters.

As shown in Figure 35, for triple-cutter interface section cutting, chemical damage at 0.60 ranks highest among all factors, followed by freeze–thaw damage at 0.40. The environmental damage external factors total 1.00 and exceed the cutter configuration internal factors at 0.49. Under triple-tooth cooperation, acidic dissolution preferentially propagates along cementation weak zones, where throughgoing interface dissolution channels sharply amplify shear dislocation and enhance cumulative wear. Freeze–thaw effects are partially suppressed as the interface stress concentration restricts microcrack propagation. Cutter spacing at 0.26 is weakly positively correlated, and penetration is at −0.23; large penetration improves crack coalescence efficiency, yet soft–hard rock layer differences partially offset fracture effects.

**Figure 36:**
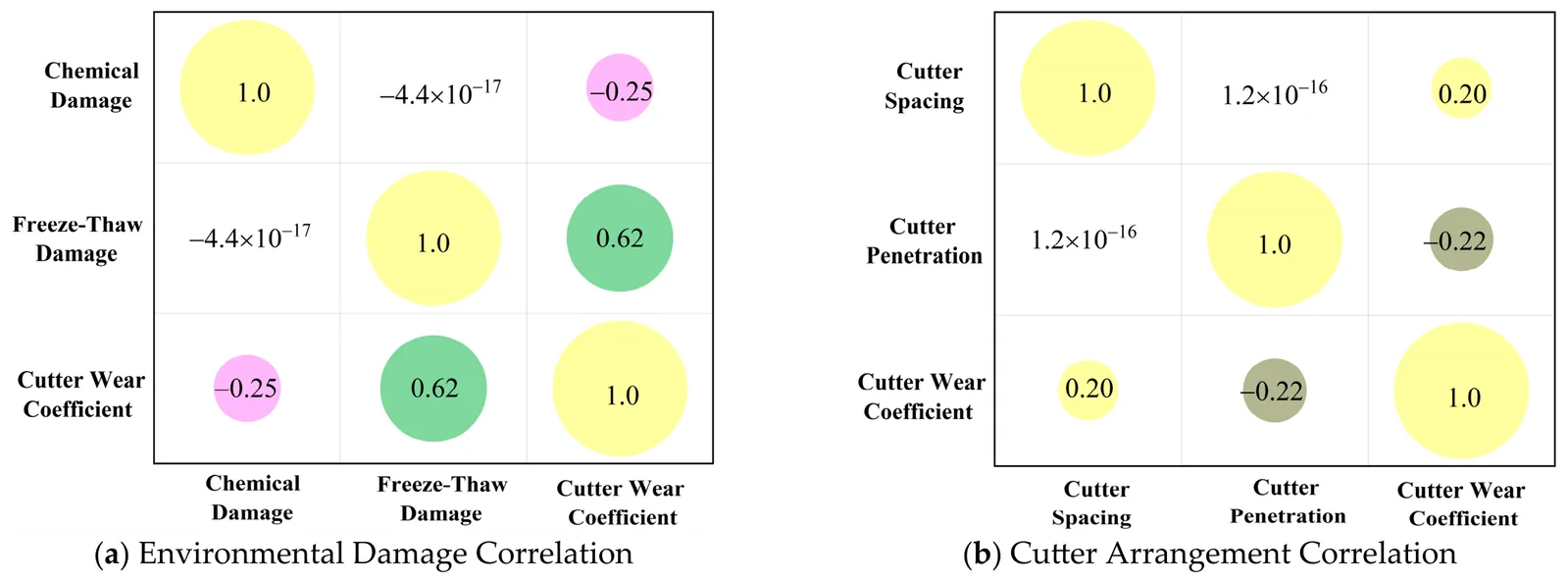
Pearson correlation matrix of single-cutter FT–T–C coupling total wear coefficient with environmental damage and cutter parameters.

As shown in Figure 36, for single-cutter total wear, freeze–thaw damage at 0.62 ranks highest among all factors, with the environmental damage external factors totaling 0.87, far exceeding the cutter configuration internal factors totaling 0.42. Freeze–thaw cycles reduce rock mass strength and enhance cuttability, yet abrasive effects of deteriorated rock mass-generated fine particles accumulate and amplify in total wear; chemical damage at −0.25 indicates that enhanced acidity softens sandstone cementitious materials and reduces cutting resistance, with lithological differences across three sections partially diluting the chemical effects. Cutter spacing at 0.20 is weakly positively correlated, as large cutter spacing increases rock ridge remnants and contact frequency, yet lithological differences across three sections disperse the cutter spacing effects in total wear. Penetration at −0.22 is weakly negatively correlated, as large penetration drives rock breaking toward fracture transformation; however, the fracture effects are partially offset by soft–hard rock layer differences under three-section coordination.

**Figure 37:**
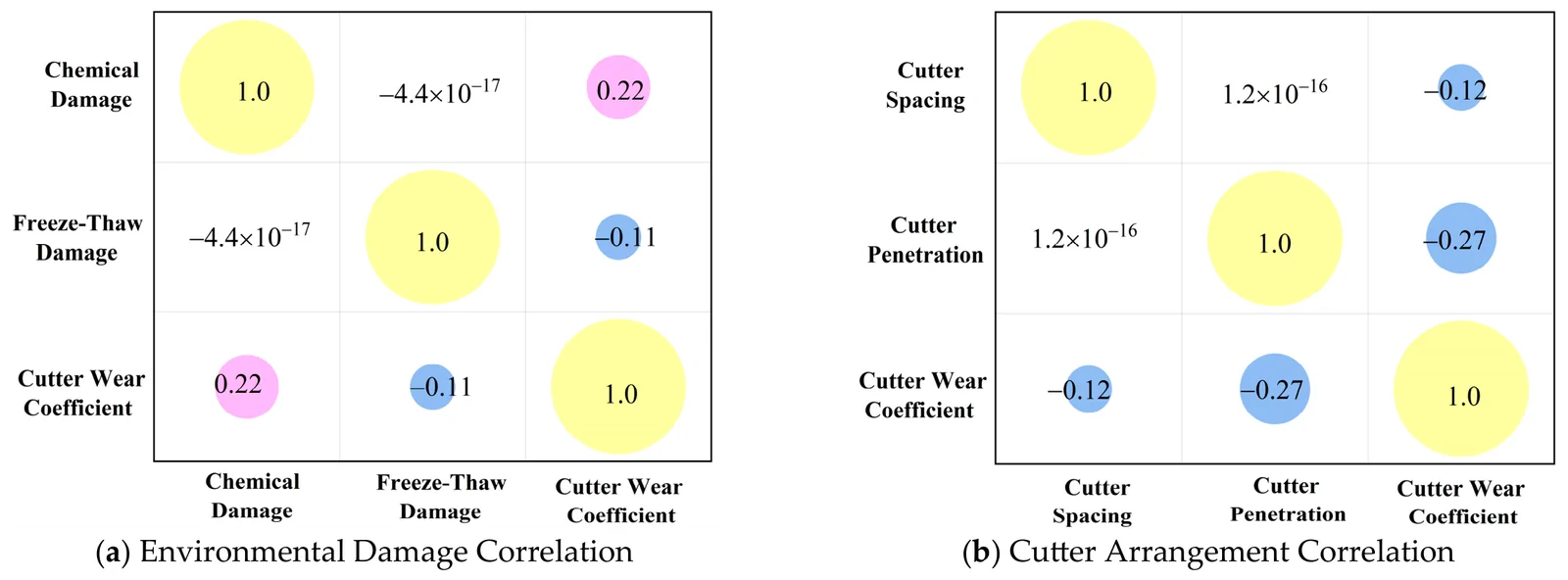
Pearson correlation matrix of double-cutter FT–T–C coupling total wear coefficient with environmental damage and cutter parameters.

As shown in Figure 37, for double-cutter total wear, cutter penetration at −0.27 jumps to the highest among all factors, with the cutter configuration internal factors totaling 0.39, surpassing the environmental damage external factors, totaling 0.33 for the first time. Chemical damage at 0.22 equals one-third of the interface value of 0.71, with lithological differences across three sections diluting its effect; freeze–thaw damage at −0.11 shows a weak negative correlation, with strength-reduction effects partially retained. Cutter spacing at −0.12 and penetration at −0.27 yield an absolute value of penetration that is double that of the cutter spacing, as large penetration drives rock breaking toward fracture transformation and reduces total cutting specific energy under three-section coordination.

**Figure 38:**
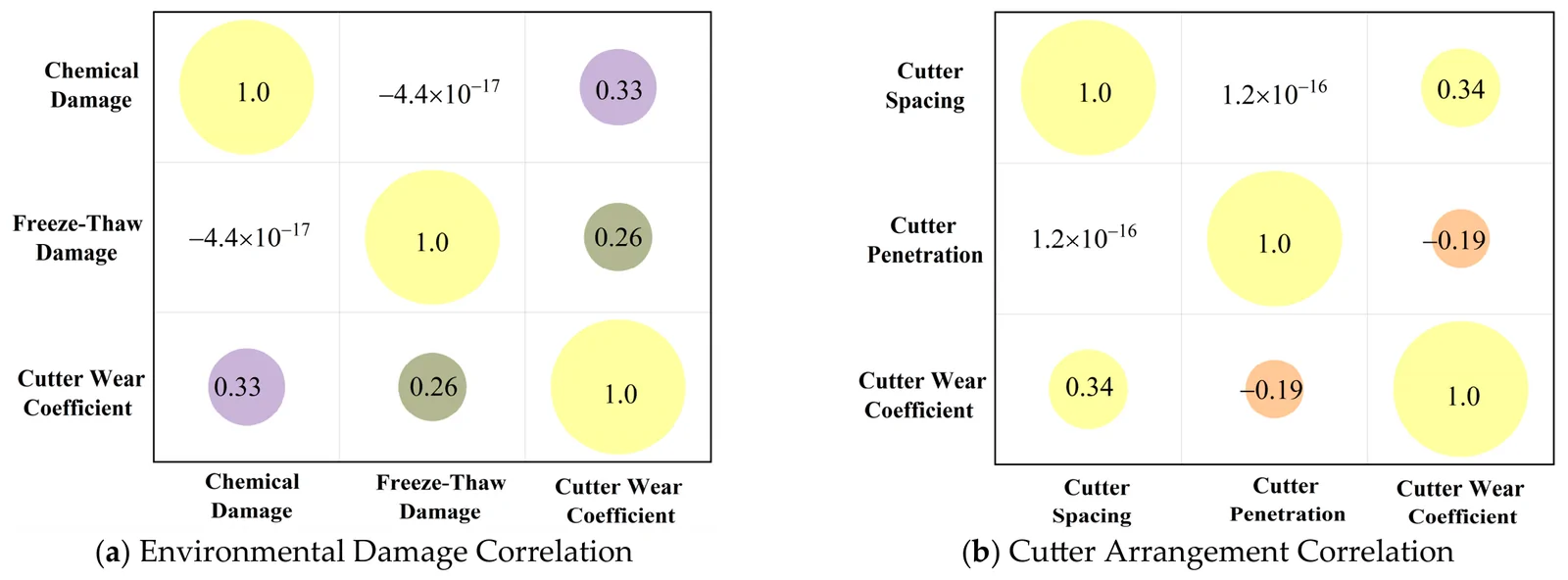
Pearson correlation matrix of total wear coefficient with environmental damage and cutter parameters under three-cutter freeze–thaw–thermal–chemical coupling.

Based on Figure 38, the total wear under the three-cutter configuration reaches its maximum correlation coefficient of 0.34 for cutter spacing. The combined internal factors of cutter configuration total 0.53, while the combined external environmental damage factors total 0.59, yielding comparable magnitudes. Chemical damage at 0.33 and freeze–thaw damage at 0.26 operate approximately independently; the intensification of acidity softens sandstone cementing materials and weakens granite grain boundaries, while the lithological variation across the three segments partially dilutes the chemical effect. Freeze–thaw deterioration reduces rock mass strength and enhances cuttability, yet the repeated crushing of rock fragments under three-cutter synergy generates fewer fine abrasive particles for recurrent compaction compared with the sandstone segment. Large cutter spacing increases residual rock ridges and contact frequency, and the three-cutter synergy complicates the geometric morphology of the crushing zone, thereby amplifying total wear. Large penetration depth drives rock breaking toward disintegration; however, the increased fragment volume between cutters obstructs discharge, and the repeated crushing effect partially counteracts the wear-reduction benefit of disintegration.

As the cutter numbers increase, the primary wear influence shifts in each segment. In the sandstone segment, it shifts from freeze–thaw environment control to penetration regulation. In the granite segment, it transitions from cutter spacing as the leading factor to dual influence by chemical damage and penetration. The interface segment remains strongly influenced by chemical damage, where cutter parameters prove nearly ineffective. At the total wear level, the primary influence transitions from freeze–thaw toward dual penetration-chemical control and ultimately toward cutter spacing prominence. The corresponding engineering strategies shift from environmental damage control to cutter configuration optimization. Chemical environment control remains an essential auxiliary measure across the entire composite stratum.

### 5.3. Sensitivity Significance Analysis of Cutter Parameters Under Multi-Damage Coupling of Cutter Cutting Thermal, Chemical, and Freeze–Thaw Effects

**Figure 39:**
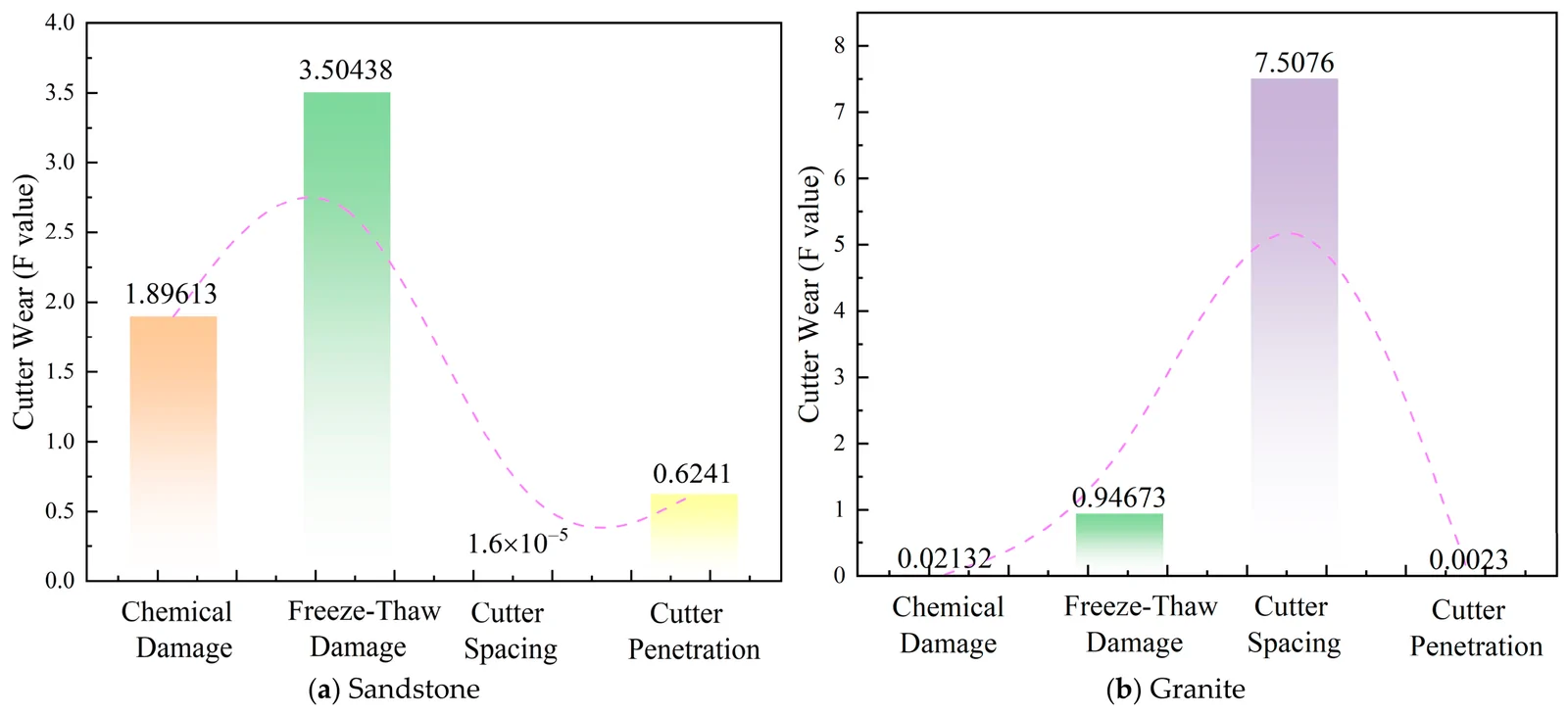

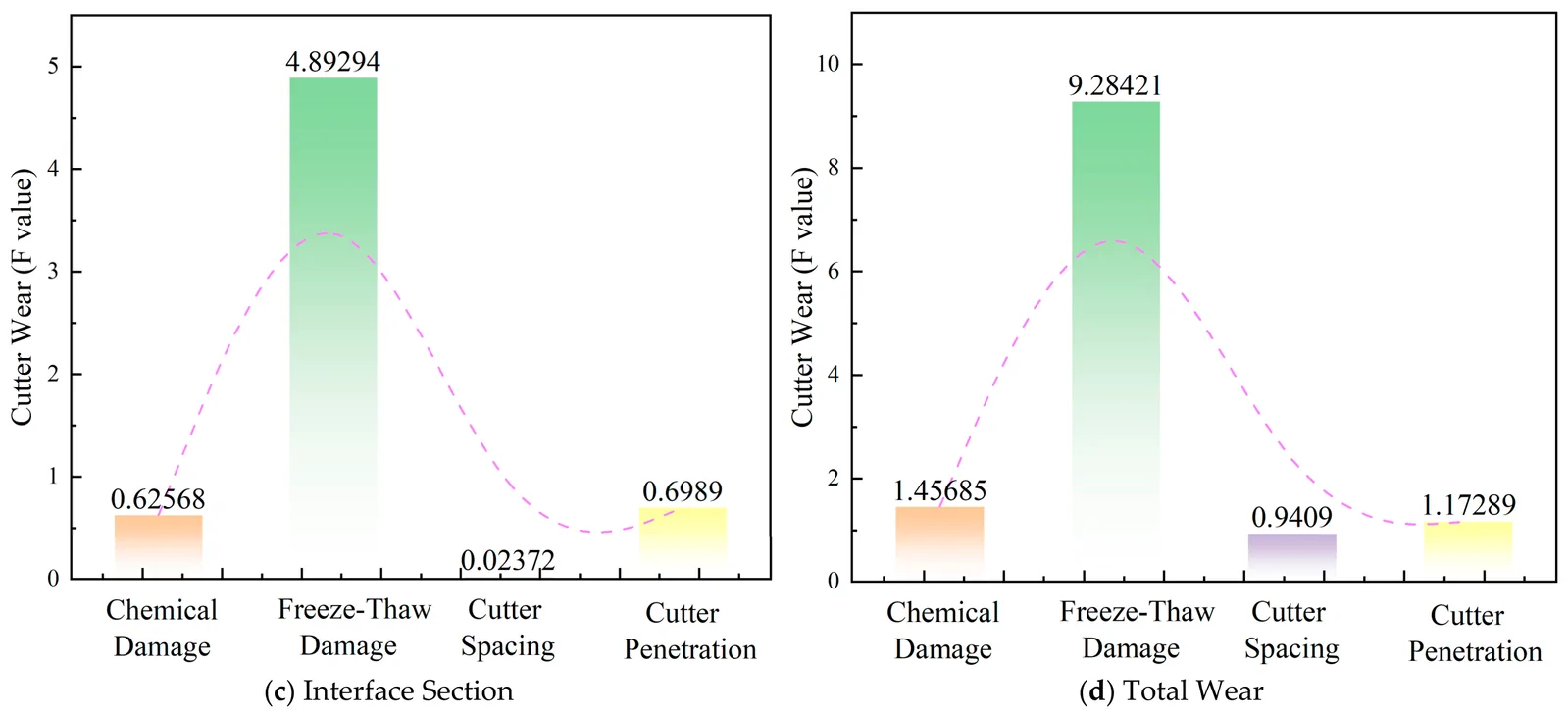
F-test significance of four factors on sandstone–granite–interface–total wear under single-cutter freeze–thaw–thermal–chemical coupling.

Based on Figure 39, under a single-cutter configuration, freeze–thaw damage registers the highest F-value of 3.50 in sandstone wear, marginally exceeding the *p* < 0.05 critical threshold of 3.49, followed by chemical damage at 1.90, while cutter spacing remains nearly ineffective. The purple trend line in the figure indicates the relative magnitude relationships among different factors. This pattern indicates that 600 °C thermal–freeze–thaw coupling intensifies cementing material degradation in sandstone. In the granite segment, cutter spacing registers the highest F-value of 7.51, statistically significant at *p* < 0.05, with freeze–thaw damage at 0.95 as secondary. The high quartz content in hard rock directly governs crack coalescence efficiency through cutter spacing control. At the interface segment, freeze–thaw damage reaches the highest F-value of 4.89, significant at *p* < 0.05, where the hardness contrast between soft and hard rocks induces shear slippage under thermal–freeze–thaw coupling. At the total wear level, freeze–thaw damage achieves the highest F-value of 9.28, significant at *p* < 0.05, while cutter spacing at 0.94 and penetration at 1.17 contribute only limitedly. The freeze–thaw–thermal coupling damage amplified in the sandstone segment propagates toward the interface, governing the total wear evolution.

**Figure 40:**
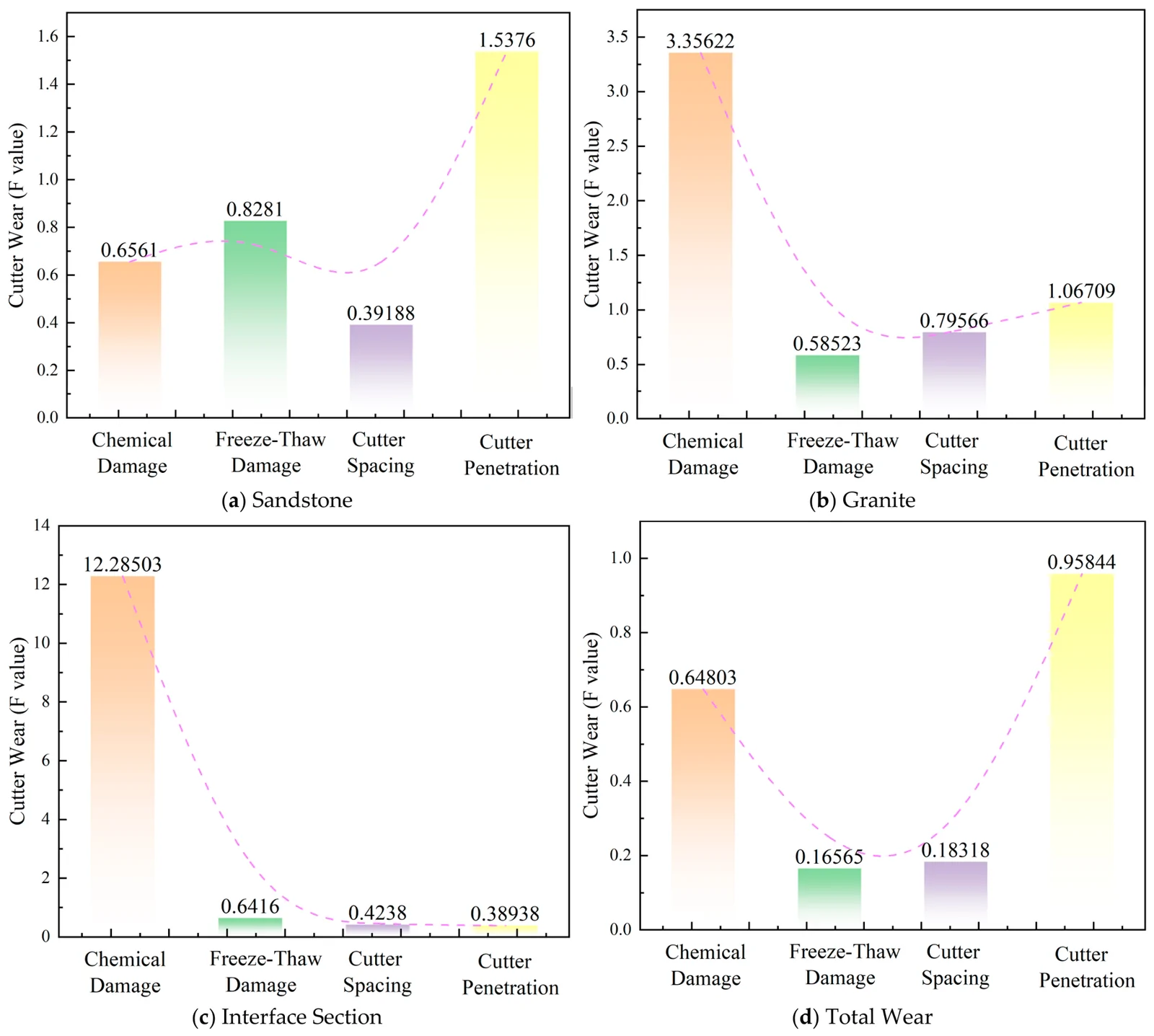
F-test significance of four factors on sandstone–granite–interface–total wear under double-cutter freeze–thaw–thermal–chemical coupling.

Based on Figure 40, under the double-cutter configuration, correlation analysis reflects the linear association direction and magnitude between factors and wear, while significance analysis reflects the explanatory weight of the factors on wear variation. In the sandstone segment, penetration achieves the largest absolute correlation coefficient at −0.32 among the four factors, and its significance proportion of 45.0% also ranks highest, demonstrating directional consistency: penetration serves as the primary controllable factor for sandstone wear. Chemical damage correlates at −0.22 and freeze–thaw damage at −0.24; in significance analysis, freeze–thaw damage at 24.3% marginally exceeds chemical damage at 19.2%, indicating that the physical destruction of sandstone cementing materials via freeze–thaw action possesses slightly stronger explanatory power than chemical dissolution.

In the granite segment, chemical damage achieves the highest correlation coefficient at 0.45 among all factors, and its significance proportion of 57.8% represents the largest contribution. Penetration correlates at 0.25 with a significance proportion of 18.4%, and cutter spacing correlates at 0.22 with 13.7%. The regulatory effect of cutter parameters remains weaker than chemical damage yet still functions as a secondary controllable factor.

At the interface segment, chemical damage achieves the highest correlation coefficient at 0.71 across all three segments, and its significance proportion of 89.4% also ranks highest. The low combined significance proportion of cutter parameters at 5.9% indicates that regulatory space is limited.

At the total wear level, penetration achieves the highest correlation coefficient at −0.27 among all factors, and its significance proportion of 49.0% also constitutes the primary factor; chemical damage correlates at 0.22 with a significance proportion of 33.1%, establishing a dual-primary pattern with consistent directionality.

**Figure 41:**
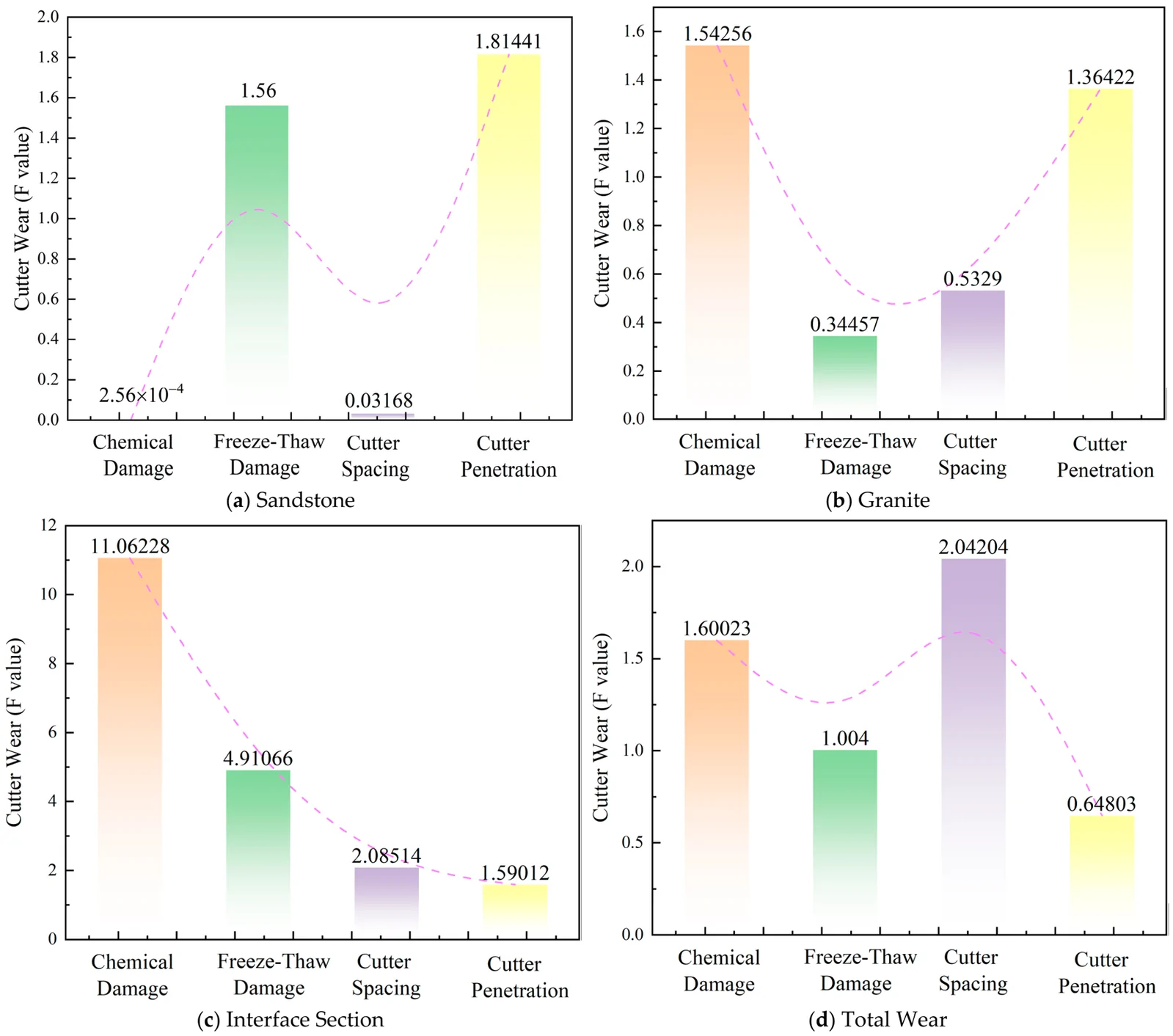
F-test significance of four factors on sandstone–granite–interface–total wear under three-cutter freeze–thaw–thermal–chemical coupling.

Based on Figure 41, under the three-cutter configuration, penetration exerts the most pronounced influence on sandstone wear with an F-value of 1.81, followed by freeze–thaw damage at 1.56, while chemical damage becomes nearly ineffective. Under multi-cutter synergy, penetration emerges as the core variable governing sandstone cutting efficiency; after 600 °C thermal softening reduces rock mass strength, the penetration depth directly determines the cutting volume per unit stroke and the cutter–rock contact stress.

In the granite segment, chemical damage achieves the highest F-value at 1.54, followed by penetration at 1.36, with freeze–thaw damage at 0.34 as the lowest. Under a dense three-cutter arrangement, the grain boundary dissolution effect of acidic environments on high-quartz granite is amplified, and chemical–thermal coupling damage emerges as a more influential wear mechanism than freeze–thaw action in hard rock.

At the interface segment, chemical damage reaches the highest F-value of 11.06 across all working conditions, followed by freeze–thaw damage at 4.91, while cutter spacing at 2.09 and penetration at 1.59 contribute only limitedly. Chemical dissolution preferentially weakens the cementing matrix on the sandstone side at the soft–hard rock interface; the overlapping cutting zone of three cutters forms an acidic solution retention band, causing interface shear strength to drop sharply and wear sensitivity to escalate dramatically.

At the total wear level, cutter spacing registers the highest F-value of 2.04, followed by chemical damage at 1.60, while freeze–thaw damage at 1.00 and penetration at 0.65 contribute weakly. In the synergistic total effect of multiple cutters, cutter spacing governs crack coalescence between adjacent cutters and rock ridge crushing efficiency, establishing itself as the critical geometric parameter for regulating overall wear in the composite formation.

### 5.4. Limitations and Future Validation Perspectives

The coupled thermal–environmental wear model in this study is subject to two interrelated limitations that jointly temper confidence in its predictions. The first concerns the validation strategy. The current framework is calibrated and cross-validated against published experimental data, including linear cutting machine tests, high-temperature hardness measurements, and mineral composition analyses from the literature. Independent laboratory experiments or field monitoring campaigns specifically designed to validate the coupled thermal, chemical, and freeze–thaw wear components under identical conditions have not yet been conducted. Consequently, the absolute wear rates and threshold predictions should be interpreted with caution until direct experimental validation is achieved.

The second limitation pertains to uncertainty quantification. The analysis relies primarily on deterministic sensitivity reasoning and linear error propagation rather than explicit statistical quantification. Although the wear coefficient formulation in Equation (17) intrinsically reduces parametric uncertainty by normalizing volumetric wear against the 100 °C baseline, the absolute bounds of the sub-microscopic mineral damage factor *D* remain qualitatively constrained by published mineral composition data and temperature–hardness fitting errors. Therefore, although the reported wear rankings and factors influence conclusions, while physically grounded, they should be regarded as indicative rather than probabilistically bounded.

These two limitations are inherently coupled. The absence of dedicated experimental data under identical coupled conditions precludes rigorous statistical uncertainty quantification, and the lack of probabilistic bounds, in turn, constrains the reliability of validation against literature data. Future work will therefore address both gaps in parallel. Temperature-controlled linear cutting machine tests will provide direct experimental evidence for model validation, while systematic field TBM data acquisition will enable the construction of empirical confidence intervals and the establishment of quantitative uncertainty bounds for the coupled wear predictions.

## 6. Conclusions

This study established a coupled FLAC3D-PFC3D numerical model and derived a relative wear calculation model, accounting for sub-microscopic mineral damage from Holm wear theory. This study systematically investigated the wear characteristics of disc cutters in sandstone–granite composite strata under multi-damage coupling conditions, focusing on the TBM cutter cutting temperature as the core engineering problem and incorporating environmental damage from chemical corrosion and freeze–thaw cycles as supplementary factors. The main conclusions are as follows:

(1) This study established a coupled FLAC3D-PFC3D model, achieving bidirectional force–velocity coupling between continuous and discontinuous media and deriving a relative wear model from Holm theory by incorporating sub-microscopic mineral damage factors. The coupled framework captures interlayer heterogeneity modulation on thermal–mechanical damage, and the segmented wear formulation resolves differential abrasiveness across sandstone–granite boundaries through a temperature-normalized wear coefficient. The model bridges mesoscopic mineral deterioration, including quartz-phase transition, feldspar chemical dissolution, and lattice thermal expansion, with macroscopic cutter wear evolution, offering a physically grounded alternative to purely data-driven wear prediction methods.

(2) Under temperature–chemical coupling, sandstone exhibits stronger synergistic damage than granite because the loose cementitious structure amplifies thermal–chemical deterioration. The wear coefficient of sandstone reached its maximum of 1.45 under 600 °C and pH 6, indicating that weakly acidic high-temperature conditions constitute the most unfavorable wear scenario for sandstone sections. In contrast, granite wear coefficients remained near or marginally above unity across all tested temperatures, indicating that dense crystalline structure and high mineral hardness effectively suppress temperature–chemical dual-field coupling damage. A double-cutter arrangement reduces wear under mild conditions through cooperative load sharing, whereas high-temperature acidic conditions intensify rock ridge secondary cutting. Large penetration combined with moderate cutter spacing reduces wear in both lithologies, with optimal geometric parameters differing across sections. These findings suggest that zoned temperature control strategies should be implemented, with strict control for sandstone, early warnings for the interface, and a relaxed standard for granite.

(3) Under temperature–freeze–thaw coupling, sandstone wear monotonically amplifies with temperature and freeze–thaw cycles, peaking at 1.75 under 600 °C and 14 freeze–thaw cycles. Granite exhibits non-monotonic fluctuation, with a local peak of 17% amplification at 400 °C and values below the baseline at 600 °C, as high-temperature densification causes cutting to be significantly influenced by brittle fracture. This divergence reveals fundamentally different responses between cemented and crystalline structures, rendering cutter life in sandstone sections more severely constrained by coupling damage. A triple-cutter dense arrangement suppresses synergistic deterioration through dual mechanisms of load reduction and heat dissipation, constituting the preferred cutter configuration among the tested arrangements for high-temperature freeze–thaw strata under the current simulation conditions.

(4) Under triple-factor coupling, the most influential wear factors shift conditionally with the cutter number and lithology. For single-cutter sandstone wear, freeze–thaw damage exhibits the highest correlation coefficient at 0.45, and environmental damage external factors collectively outweigh cutter configuration internal factors. For double-cutter sandstone wear, cutter penetration reaches the largest absolute correlation coefficient at −0.32, and internal and external factor groups exhibit comparable influence magnitudes. For triple-cutter sandstone wear, cutter penetration at 0.35 emerges as the most influential factor, while chemical damage becomes nearly irrelevant under multi-cutter synergy due to rock chip secondary crushing effects. In the granite section, the influential factor transitions from cutter spacing under a single-cutter configuration to chemical damage under double-cutter and triple-cutter configurations. At the interface section, chemical damage consistently exhibits a strong positive correlation, reaching 0.71 under the double-cutter configuration and 0.60 under the triple-cutter configuration, as acidic dissolution preferentially propagates along sandstone–granite cementation weak zones. At the total wear level, the influential factor evolves from freeze–thaw damage under the single-cutter configuration to the dual influence of penetration and chemical damage under the double-cutter configuration, and finally to cutter spacing under the triple-cutter configuration. These shifts reflect the conditional dependency of geometric parameter regulation on the cutter number and lithology rather than contradictions in the underlying physics. The corresponding engineering strategies shift from environmental damage control toward cutter configuration optimization as the cutter number increases, yet chemical environment control remains an essential auxiliary measure across the entire composite stratum.

## Figures and Tables

**Table 1:** Micro-mechanical and thermal parameters of sandstone and granite in the PFC3D model.

Rock Type	*E*_p_ (GPa)	*µ*_p_	*K*_p_	*σ*_p_ (GPa)	*C*_p_/*σ*_p_	*φ*_p_ (°)	*R*_sd_	*θ*_p_	*Thexp*
Sandstone	3.5	0.1	3.45	6.6	2.7	30	0.3	0.315	24 × 10^−6^
Granite	55	0.3	3.45	36.8	2.7	30	0.3	0.7	7.6 × 10^−6^

**Table 2:** Engineering geological parameter analogy between Xiangshan Tunnel [25] and Haojiagou [27].

Engineering Case Names	Strata Type Traversed	Uniaxial Compressive Strength	Rock Type	Burial Depth
ZK11B-6 Haojiagou	Composite Strata	40 MPa–120 MPa	Relatively Hard Rock–Hard Rock	133.98 m–212.68 m
Longxia Railway Xiangshan Tunnel	Composite Strata	58.7 MPa–99.7 MPa	Relatively Hard Rock–Hard Rock	160 m

**Table 3:** Comparison of errors between PFC3D linear cutting simulation values and test values of Cho et al. [1].

Cutter Cutting Calculation Parameters	Cutter Spacing /(mm)	Penetration /(mm/rev)	Laboratory Experiment	Numerical Simulation	Error (%)
Average Rolling Force	28.0	4.0	5.8	6.0	3.4
	40.0	4.0	7.5	7.3	2.7
	48.0	4.0	9.6	8.5	9.4
Rock-Breaking Specific Energy	28.0	4.0	56.2	57.5	2.3
	40.0	4.0	46.9	49.1	4.7
	48.0	4.0	54.7	55.2	0.9

**Table 4:** Full factorial test scheme of cutter penetration and spacing under 600 °C and pH 6 conditions.

Cutter Penetration (mm/rev)	Cutter Spacing (mm)	Cutter Penetration (mm/rev)	Cutter Spacing (mm)	Cutter Penetration (mm/rev)	Cutter Spacing (mm)
5	60	6	60	7	60
	70		70		70
	80		80		80
	90		90		90

**Table 5:** Single-cutter FT–T–C coupling four-factor four-level orthogonal test scheme and wear coefficients.

Chemical pH	Freeze–Thaw Cycles	Cutter Spacing (mm)	Cutter Penetration (mm/rev)	Wear Coefficient (Sandstone)	Wear Coefficient (Granite)	Wear Coefficient (Interface Section)
4	5	60	5	1.41	0.96	0.38
4	8	70	6	1.89	0.81	0.61
4	11	80	7	2.57	0.96	0.77
4	14	90	8	1.43	1.08	0.71
5	5	70	7	1.03	0.89	0.64
5	8	60	8	1.35	0.82	0.93
5	11	90	5	1.60	1.04	1.34
5	14	80	6	1.72	1.01	1.72
6	5	80	8	1.33	0.98	0.58
6	8	90	7	1.32	0.98	1.09
6	11	60	6	1.65	0.94	1.88
6	14	70	5	1.75	0.99	0.93
7	5	90	6	1.19	1.06	0.85
7	8	80	5	1.63	0.82	0.72
7	11	70	8	1.52	0.99	0.57
7	14	60	7	1.49	0.86	1.32

**Table 6:** Double-cutter FT–T–C coupling four-factor four-level orthogonal test scheme and wear coefficients.

Chemical pH	Freeze–Thaw Cycles	Cutter Spacing (mm)	Cutter Penetration (mm/rev)	Wear Coefficient (Sandstone)	Wear Coefficient (Granite)	Wear Coefficient (Interface Section)
4	5	60	5	1.40	0.783	0.736
4	8	70	6	1.85	0.752	0.823
4	11	80	7	1.52	0.695	0.882
4	14	90	8	1.21	0.895	0.632
5	5	70	7	2.17	0.842	0.797
5	8	60	8	1.62	0.758	1.525
5	11	90	5	1.77	0.798	1.092
5	14	80	6	1.32	0.893	1.471
6	5	80	8	1.38	0.814	1.281
6	8	90	7	1.57	0.791	0.939
6	11	60	6	1.80	0.690	1.129
6	14	70	5	1.91	0.814	1.420
7	5	90	6	1.56	0.926	1.556
7	8	80	5	1.34	0.794	1.552
7	11	70	8	1.11	0.960	1.087
7	14	60	7	1.34	0.920	1.699

**Table 7:** Triple-cutter FT–T–C coupling four-factor four-level orthogonal test scheme and wear coefficients.

Chemical pH	Freeze–Thaw Cycles	Cutter Spacing (mm)	Cutter Penetration (mm/rev)	Wear Coefficient (Sandstone)	Wear Coefficient (Granite)	Wear Coefficient (Interface Section)
4	5	60	5	1.59	0.882	0.408
4	8	70	6	1.26	0.834	1.117
4	11	80	7	1.38	0.731	1.620
4	14	90	8	1.44	0.891	1.506
5	5	70	7	1.50	0.694	0.845
5	8	60	8	1.36	0.723	0.892
5	11	90	5	1.44	0.966	1.544
5	14	80	6	1.19	0.736	1.885
6	5	80	8	1.52	0.822	1.216
6	8	90	7	1.53	0.908	0.736
6	11	60	6	1.29	0.886	1.032
6	14	70	5	1.19	0.839	2.159
7	5	90	6	1.38	0.718	2.357
7	8	80	5	1.25	0.717	2.880
7	11	70	8	1.40	0.672	1.880
7	14	60	7	1.62	0.784	2.285

## Data Availability

The original contributions presented in this study are included in the article material. Further inquiries can be directed to the corresponding author.
